# Diastereoselective synthesis of vicinal tertiary and *N*-substituted quaternary stereogenic centers by catalytic hydroalkylation of dienes†

**DOI:** 10.1039/c5sc04908c

**Published:** 2016-03-11

**Authors:** Matthew J. Goldfogel, Simon J. Meek

**Affiliations:** a Department of Chemistry, University of North Carolina at Chapel Hill Chapel Hill NC 27599-3290 USA sjmeek@unc.edu http://www.chem.unc.edu/people/faculty/meek/

## Abstract

An efficient and diastereoselective (CDC)–Rh-catalyzed hydroalkylation of dienes with 1,3-oxazol-5(4*H*)-ones is reported. Aryl and alkyl substituted dienes are converted to α,α-substituted oxazolones (24 examples) by the formation of *N*-substituted quaternary carbon stereogenic centers in good yields (up to 96%) and with high diastereoselectivity (>20 : 1 dr). The reaction is tolerant of a range of dienes and oxazolones bearing various functional groups. Utility of the oxazolone products is illustrated through hydrolysis to form α,β-substituted α-amino acid analogues and stereoselective epoxidation of the resultant alkene to create four contiguous stereocenters.

## Abstract 2

An efficient and diastereoselective (CDC)–Rh-catalyzed hydroalkylation of dienes with 1,3-oxazol-5(4*H*)-ones is reported.

## Graphical abstract

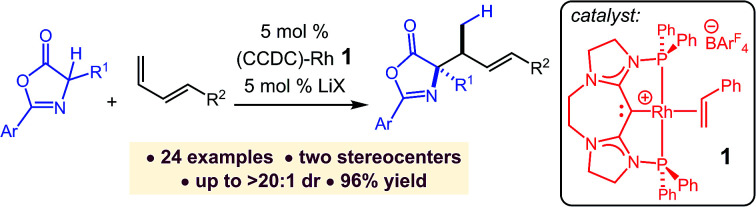

## Introduction

The catalytic hydroalkylation of alkenes is a valuable, atom-economical approach for the synthesis of C–C bonds from readily available starting materials.^1^ Pioneering studies have led to the development of intermolecular processes that employ styrenes,^2^ unactivated alkenes,^3^ allenes,^4^ and alkynes^5^ as effective substrates that can react with appropriate C-based nucleophiles. Despite recent advances in alkene hydroalkylation, intramolecular^6^ examples predominate and most transformations utilize nucleophiles that are malonate derived or trade atom-economy for reactivity.^7^ While this reaction is often applied to the transformation of alkenes, the intermolecular hydroalkylation of dienes remains relatively unexplored. The hydroalkylation of diene substrates is synthetically useful; such reactions convert readily available unsaturated hydrocarbons into versatile allyl-containing building blocks. Only a limited number of catalytic intermolecular hydroalkylations of dienes have been reported, with none able to effectively promote the diastereoselective addition of C-based nucleophiles to terminal dienes such as 3.

Catalytic intermolecular diene hydroalkylation was first accomplished with a Pd catalyst by Takahashi.^8^ Subsequent Pd catalyzed hydroalkylations have introduced a variety of enolizable nucleophiles.^9^ These reactions selectively generate linear products *via* 1,4-addition with modest to excellent site-selectivity. The reactions work well with 2,3-substituted dienes but for 1,4-substituted dienes reactions are limited to methyl-substituted or cyclic substrates (*e.g.*, cyclohexadiene).^8^

**Scheme 1:**
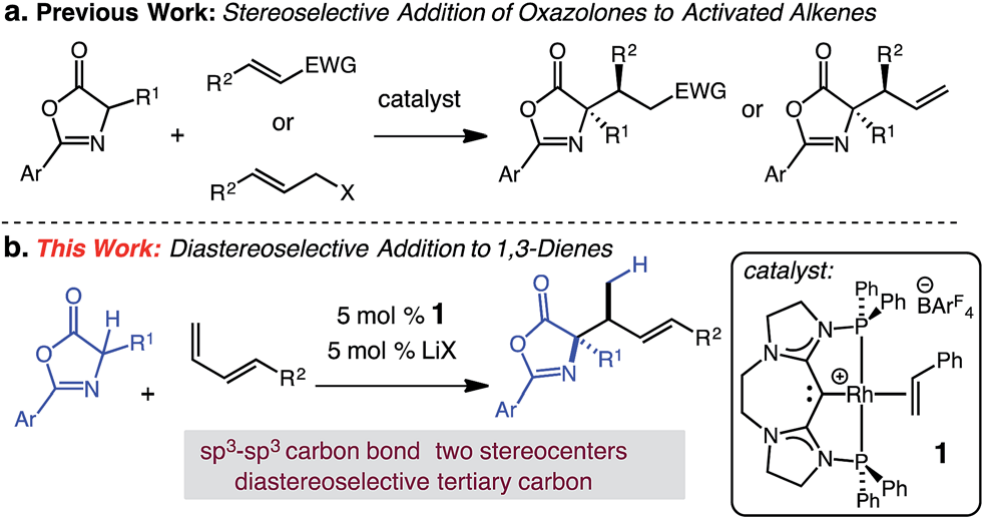
Diastereoselective (CDC)–Rh(i) catalyzed hydroalkylation of dienes with 1,3-oxazol-5(4*H*)-ones.

We postulated that the catalyst controlled γ-selective addition of a prochiral enol nucleophile to 1-substituted diene would enable C(sp^3^)–C(sp^3^) formation and generate vicinal strereogenic centers. Enantioselective Michael additions of 1,3-oxazol-5(4*H*)-ones to activated C–C π bonds^10^ has been demonstrated (aldehydes,^11^ ketones,^12^ amides,^13^ allenoates^14^), however, despite these notable advances, additions to unactivated alkenes or dienes substrates have not been reported. In this report, we describe a catalytic addition of substituted 1,3-oxazol-5(4*H*)-ones to 1-substituted dienes that is site- and diastereoselective (Scheme 1).^15^

We have previously developed carbodicarbene (CDC) Rh complexes for the catalytic hydroamination^16^ and hydroarylation^17^ of dienes under mild conditions in the presence of various polar functional groups.^18^ The reported carbodicarbene (CDC)-supported Rh catalyst 1 exhibits secondary activation through the reversible interaction of Lewis acids and the CDC ligand,^19^ which modulates electron donation to the Rh center (*i.e.*, increasing the electrophilicity of the catalyst).^17^ This reversible activation lessens inhibition by the nucleophile. We sought to extend the use of this catalyst to the formation of C(sp^3^)–C(sp^3^) carbon bonds and the diastereoselective generation of two contiguous stereogenic centers.

Herein, we present the first diastereoselective catalytic addition of oxazolones to 1-substituted dienes. The reactions generate α,α-disubstituted allylic amino acid products in up to 96% yield and >20 : 1 dr. Products contain two contiguous stereocenters, including an *N*-substituted quaternary carbon. The transformation is catalyzed by 5 mol% of the (CDC)–Rh catalyst in the presence of 5 mol% lithium salt at 50 °C and affords the *anti*-addition products.

## Results and discussion

We began our studies for an efficient catalytic hydroalkylation of dienes by the reaction of oxazolone 2 with phenylbutadiene 3 in the presence of 5 mol% (CDC)–Rh complex 1 and 5 mol% LiPF_6_ activator in toluene at 50 °C (Table 1, entry 1). We were encouraged to observe the formation of 4 in 17% yield and 10 : 1 dr (*anti*/*syn*). A brief survey of solvents provided slightly higher yields (up to 21%), but reduced diastereoselectivities (<6 : 1 dr) in all cases (see ESI†). As such, toluene was used for further optimization. A variety of Lewis acid activators were screened (entries 2–4, Table 1) and demonstrated that lithium salts were most effective; 5 mol% of AgCl and LiBF_4_ resulted <10% conversion to 4 most likely the due to poor solubility of the metal salt in toluene. Weaker coordinating counter anions lead to dramatically improved reactivity as shown by LiBAr^F^_4_, which furnishes 4 in 51% yield with 18 : 1 dr. Further increase in reaction efficiency can be achieved through the use of alcohol co-solvents (entries 5–8). While addition of i-PrOH led to no improvement in the LiBAr^F^_4_ promoted reaction (50% yield, 11 : 1 dr, entry 5), treatment of 3 and 4 with 5 mol% (CDC)–Rh and 5 mol% LiPF_6_ in toluene/i-PrOH 40 : 1 at 50 °C proved optimal, delivering 4 in 85% yield and 19 : 1 dr (entry 6). Screening various alcohol co-solvents results in both decreased conversion and selectivity (entries 7 and 8); MeOH and *t*-BuOH afforded 4 in 26% yield (3 : 1 dr), and 29% yield (5 : 1 dr), respectively. It should be noted that MeOH as a co-solvent leads to competitive oxazolone decomposition *via* ring-opening. The conditions reported in entry 6 were identified as optimal and employed in further reaction development, although LiBAr^F^_4_ was found to be optimal for certain substrates (*vide infra*). Additional control reactions run without LiPF_6_ (entry 9), or with 2.5 mol% [Rh(cod)Cl]_2_ and 5 mol NaBAr^F^_4_ in place of (CDC)–Rh 1 (entry 10) result in no reaction, highlighting the importance of cationic (CDC)–Rh complex 1, in combination with a Lewis acid co-catalyst, for reactivity.

**Table 1:** Survey of conditions for diastereo selective (CDC)–Rh-catalyzed hydroalkylation 1,3-diene 3 with 1,3-oxazol-5(4*H*)-one 2^a^

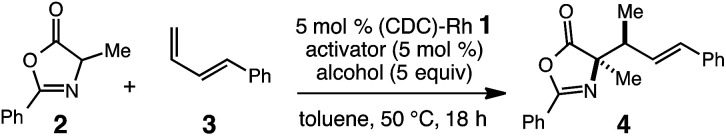			
Entry	Activator; mol%	Alcohol^b^	Yield^c^ (%); dr^d^
1	LiPF_6_; 5	—	17; 10 : 1
2	AgCl; 5	—	0; —
3	LiBF_4_; 5	—	8; 4 : 1
4	LiBAr^F^_4_; 5	—	51; 18 : 1
5	LiBAr^F^_4_; 5	i-PrOH	50; 11 : 1
**6**	**LiPF**_**6**_ **; 5**	**i-PrOH**	**85; 19 : 1**
7	LiPF_6_; 5	MeOH	26; 3 : 1
8	LiPF_6_; 5	*t*-BuOH	29; 5 : 1
9	—	i-PrOH	0; —
10^e^	LiPF_6_; 5	i-PrOH	0; —

a See ESI for experimental details; all reactions performed under N_2_ atm.

b A solvent ratio of 40 : 1 toluene/alcohol used.

c Yields of purified products are an average of two runs.

d Values determined by analysis of 400 or 600 MHz ^1^H NMR spectra of unpurified mixtures with hexamethyldisiloxane as an internal standard.

e Reaction run with 2.5 mol% [Rh(cod)Cl]_2_ and 5 mol% NaBAr^F^_4_ as catalyst.

Determining the role of the alcohol and its influence on reaction efficiency and diastereoselectivity cannot necessarily be decoupled, however, a number of observations can be made: (1) the lithium salt is required for the reaction to occur. (2) Considering the difference in reaction efficiency between LiPF_6_ and LiBAr^F^_4_ without i-PrOH, the alcohol is likely assisting in solubilizing the lithium salt. (3) Addition of i-PrOH changes the product diastereoselectivity, either suggesting hydrogen bonding of the alcohol with the nucleophile or alcohol solvation of the lithium salt. In the case of LiPF_6_ dr increases where as for LiBAr^F^_4_ the selectivity decreases. (4) Additional roles, such as formation of Brønsted acid, are disfavored by reactions run in the presence of 2,6-di*tert*-butyl pyridine, which show no deleterious effects (see ESI† for details). (5) Use of chiral alcohols and diols do not result in an enantioselective reaction (see ESI† for details).

With optimized conditions in hand, we sought to explore the diene scope of the hydroalkylation with oxazolone 2. For certain diene substrates LiBAr^F^_4_ proved to be the more effective lithium salt in order to obtain good yields and selectivities. As shown in Table 2, formation of the *N*-substituted quaternary carbon occurs readily with modest to excellent levels of selectivity with aryl (5–13) and alkyl dienes (14–16). Electronic modifications to the aryl ring were well tolerated by the reaction. Aryl rings bearing halogens or electron withdrawing groups react with only slight decreases to yield and diastereoselectivity; *p*-Cl-, *p*-F- and *p*-NO_2_-phenylbutadienes react to give 5 in 67% yield (19 : 1 dr), 6 in 70% yield (6 : 1 dr), and 7 in 48% yield (8 : 1 dr), respectively. Electron-rich arenes are also compatible, but result in reduced *anti*/*syn* diastereoselectivity; *p*-MeO-phenylbutadiene reacts to form 8 in 58% yield and 4 : 1 dr. Phenylbutadiene containing alkyl substitution at the *ortho*-, *meta*- and *para*-positions of the aryl ring are excellent substrates providing 9 in 59% yield (>20 : 1 dr), 10 in 66% yield (>20 : 1 dr), and 11 in 89% yield (6 : 1 dr). The high selectivity in the formation of 9 and 10 demonstrates the influence of sterics and its translation to increased diastereoselectivity in C–C bond formation with only slight decreases in yield. Dienes bearing oxygen heterocycles participate in the hydroalkylation reaction with 12 formed in 91% and in 9 : 1 dr; however, pyridyl groups appear to inhibit catalyst 1 as 13 does not form under the same reaction conditions.^20^ Alkyl-substituted dienes are effective substrates and react with oxazolone 2 to produce alkenyl products 14–16 in good yields and selectivities (Table 2). We anticipated that the decreased size of the alkyl chain, compared to an aryl ring, would result in diminished diastereoselectivity, however, the opposite was observed; 14 was formed in high diastereoselectivity (12 : 1 dr) in 66% yield. The increased α-branching in cyclohexylbutadiene results in lower reactivity and diastereoselectivity, providing 15 in 43% yield but in 3 : 1 dr. Additionally, the mild reaction conditions are tolerant of silyl ether functionality; for example homoallyl TBS ether 16 is delivered in 68% yield and 4 : 1 dr without silyl ether deprotection or elimination to form the conjugated diene. For some substrates lower conversions can be observed due to competitive ring-opening of the oxzalone by i-PrOH, however, yields can often be improved by increasing the equivalents of the nucleophile.

**Table 2:** (CDC)–Rh(i)-catalyzed hydroalkylation of substituted dienes^a^

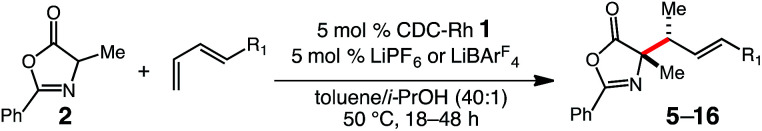
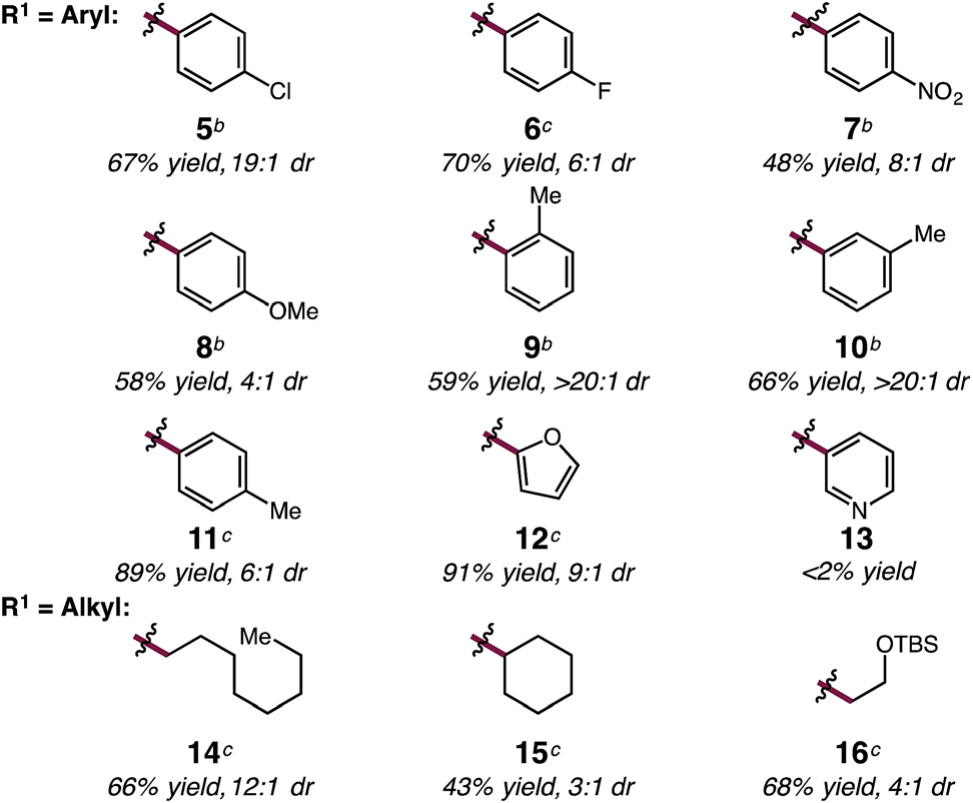

a See ESI for experimental details.

b 5 mol% of LiPF_6_.

c 5 mol% of LiBAr^F^_4_. All reactions performed under N_2_ atm. Yields of purified products are an average of two runs.

We next examined the scope of the oxazolone nucleophiles tolerated by (CDC)–Rh catalyst 1. To explore the interplay between the identity of the oxazolone substituent and diene, oxazolones were reacted with representative dienes bearing heterocyclic, alkyl and aryl motifs to afford 17–28 (Table 3). Extension from methyl- to *n*-propyl-substituted oxazolones (17–19) resulted in high conversions, similar to those obtained with 2, but with lower selectivity compared to 9, 12 and 14; 17 was produced in 89% yield with 6 : 1 dr, 18 was synthesized in 55% yield with 7 : 1 dr, and 19 was formed in 57% yield with 10 : 1 dr. The lower selectivity may be a consequence of increased sterics on the α-substituent influencing the orientation of the nucleophile as it approaches the activated diene. Reactions with *sec*-butyl-substituted oxazolone demonstrate that β-branched alkyl substituents work effectively as 20–22 are formed in good to high yields with varying selectivity; 20 is formed in 96% yield with 19 : 1 dr, while 21 is synthesized in 51% yield with 8 : 1 dr and 22 in 89% yield with 10 : 1 dr. To further demonstrate that increased substitution on the oxazolone is viable, phenethyl-oxazolone was reacted to give 23–25; 23 was formed in 57% yield with 5 : 1 dr, 24 in 21% yield with 7 : 1 dr and 25 in 50% yield with 10 : 1 dr. The (CDC)–Rh catalyst is compatible with alkenes as evidenced by the successful formation of 26 in 28% yield with 5 : 1 dr. The reduction in yield is from competitive isomerization of the allyl group to the internal alkene, which is not a competent nucleophile. Furthermore, formation of 27 (2 : 1 dr) demonstrates the subtle effect that the sterics of the diene play in obtaining a selective reaction (*cf.*, 22, generated in 20 : 1 dr). We were able to modify the aryl substituent of the oxazolone, as demonstrated by the formation of 28; *p*-Cl-phenyl-oxazolone reacted to provide 28 in 71% yield and 3 : 1 dr, however, the site-selectivity of the reaction decreased to give a 11 : 1 mixture of γ,δ- and α,δ-regioisomers.

**Table 3:** Oxazolone scope for (CDC)–Rh(i)-catalyzed hydroalkylation^a^

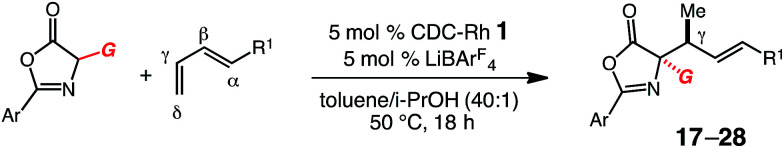
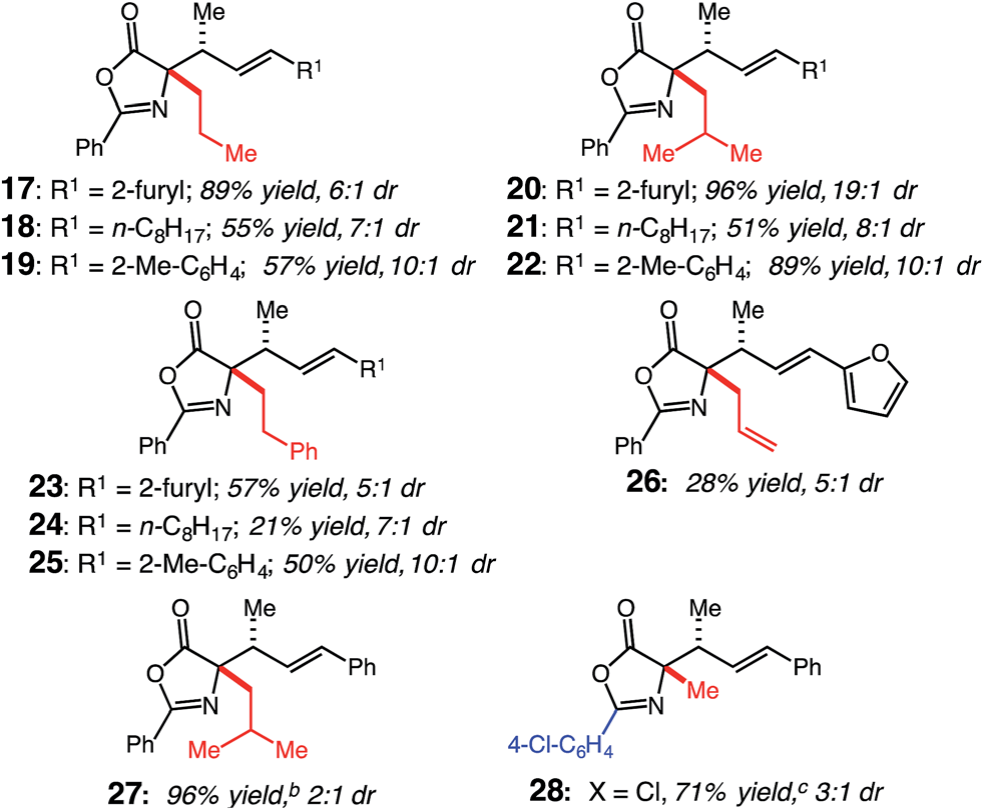

a See ESI for experimental details. All reactions performed under N_2_ atm. Yields of purified products are an average of two runs.

b Formed as a 20 : 1 mixture of the α- and γ-regioisomers.

c Formed as a 1 : 11 mixture of α- and γ-regioisomer.

**Scheme 2:**
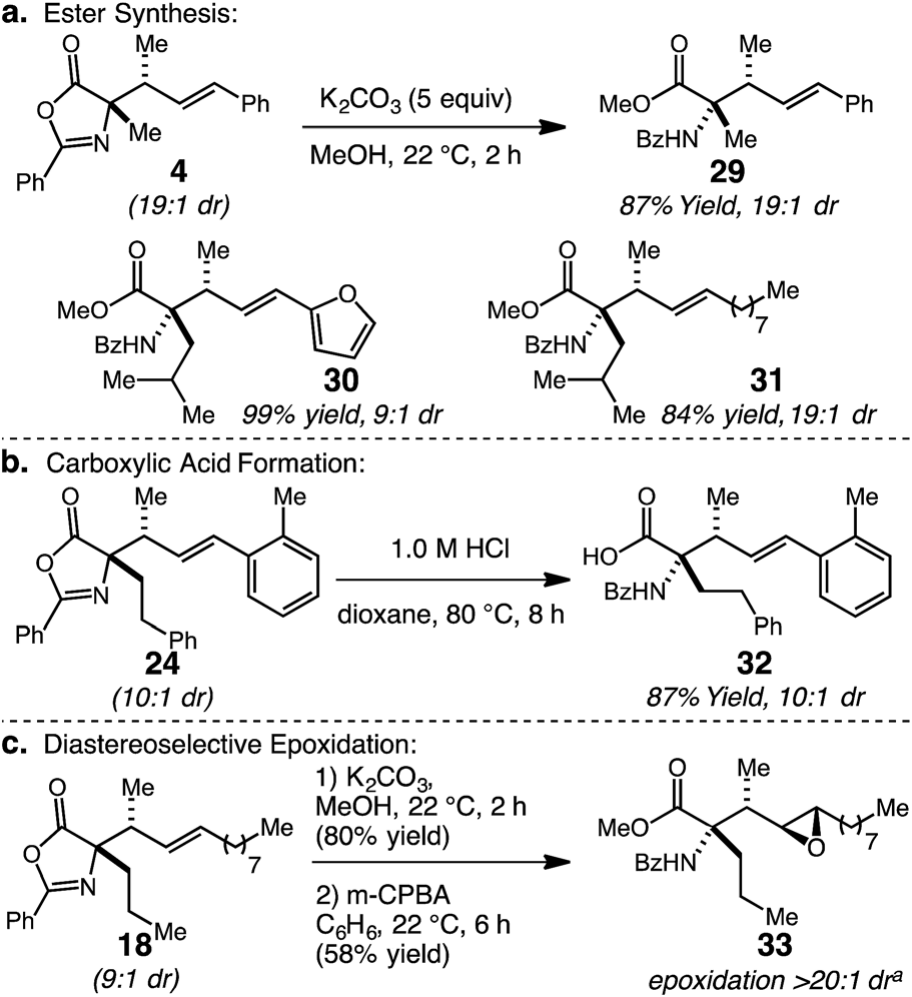
α-Allyl oxazol-5(4*H*)-one functionalizations. ^*a*^Determined by analysis of 600 MHz ^1^H NMR spectra of unpurified mixture.

The substituted oxazolone products generated through the catalytic stereoselective hydroalkylation protocol can be readily transformed to other useful molecules (Scheme 2). Firstly, ring-opening of allyl-substituted oxazolone 4 with MeOH and K_2_CO_3_ at 22 °C delivers methyl esters 29 in 87% yield; synthesis of 29 confirmed the *anti* diastereoisomer as the major product by comparison to previously reported data.^15*d*^ Two additional α,α-disubstituted products, 20 and 21, were converted to their corresponding methyl esters 30 and 31 in 99% and 84% yield, respectively. Second, conversion of the oxazolone moiety to benzoyl-protected amino acids can be accomplished by acid hydrolysis; the conversion of 24 to 32 with dilute HCl in 87% yield is representative. Finally, the vicinal tertiary allylic, and *N*-substituted quaternary stereocenters can be used to impart stereocontrol in further alkene functionalizations. In this regard, 18 (9 : 1 dr) was successively hydrolyzed to the methyl ester and subjected to *m*-CPBA epoxidation to form epoxide 33 in 58% yield over two steps with complete stereocontrol.^21^ The resulting product contains four contiguous stereocenters and a versatile epoxide ring, which could be opened stereoselectively to introduce a variety of nucleophiles.^22^

**Scheme 3:**
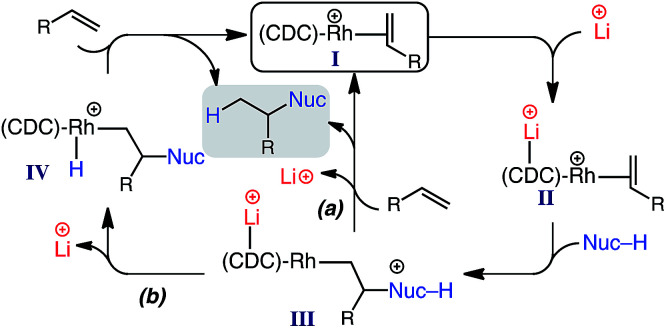
Proposed catalytic cycle.

According to our data, a possible catalytic cycle for the reported hydroalkylation is depicted in Scheme 3. While the specific role of the lithium salt is not yet determined, previous studies indicate that secondary binding to the CDC carbon (*e.g.*, II) decreases electron density at the Rh center, resulting in decreased π-back donation,^17^ and thus facilitating nucleophile addition (II → III).^23^ Product formation and regeneration of I could occur by two possible pathways: (a) direct Rh–alkyl protonation; (b) proton transfer to Rh and subsequent reductive elimination.^24^

## Conclusions

In summary, we have demonstrated the first diastereo- and siteselective hydroalkylation of 1-substituted 1,3-dienes with oxazolone nucleophiles promoted by a cationic (CDC)-Rh catalyst. The use of a catalytic lithium salt activator, and alcohol serve to provide optimal reactivity and good diastereoselectivity under mild conditions for a range of dienes with oxazolone nucleophiles. The resulting products contain two contiguous stereocenters and an *N*-substituted quaternary center, which can be deprotected to generate useful amino acid analogues, or exploited to impart acyclic stereocontrol in alkene epoxidation. Related studies are in progress to expand the scope of carbon-based nucleophiles and alkene electrophiles in hydroalkylation processes as well as development of enantioselective variants.

## Supplementary Material

SC-007-C5SC04908C-s001
