# Supplementary material for: Diastereoselective synthesis of vicinal tertiary and N-substituted quaternary stereogenic centers by catalytic hydroalkylation of dienes
Source: Chem Sci. 2016 Mar 11;7(7):4079–84. doi: 10.1039/c5sc04908c (PMC6013916; doi:10.1039/c5sc04908c)
Supplement: SC-007-C5SC04908C-s001 [file SC-007-C5SC04908C-s001.pdf]

Electronic Supplementary Material (ESI) for Chemical Science.
This journal is © The Royal Society of Chemistry 2016

Diastereoselective Synthesis of Vicinal Tertiary and N-Substituted Quaternary Stereogenic Centers by Catalytic Hydroalkylation of Dienes

Matthew J. Goldfogel and Simon J. Meek*

Department of Chemistry, The University of North Carolina at Chapel Hill, Chapel Hill, North Carolina 27599-3290.

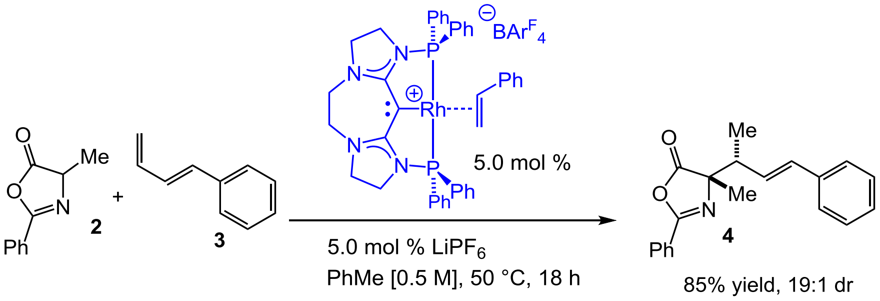

SUPPORTING INFORMATION

Table of Contents

General...……………………………………………………………………………….……...S1
Solvents ………………………………………………………………………………………S2
Reagents……………………………………………………………………………...………S2
General procedure for the hydroalkylation in Tables 1, 2 and 3…………….............… …...S3
Procedures and characterization of α,α-substituted oxazolone products………………..………S3
General procedure for hydrolysis of oxazolone products in Table 4…………….……….……..S21
Procedure and characterization of hydrolyzed oxazolone products in Table 4…………… …...S21
Procedure and characterization for the functionalization of oxazolone products in Table 5. …S23
Procedure for exploring the effect of the alcohol additive on hydroalkylation…......…………S24
Data table for the effect of the alcohol on diastereo- and enantioselectivity……… ………..….S25
Extended data table for reaction optimization (Table 1) ……………………………………..…S25
References…………………………………………………………………………………S25
NMR Spectra …………………………………………………………………….. ……S27

■ **General:** All reactions were carried out in flame or oven (140 ^oC) dried glassware that had been cooled under vacuum. Unless otherwise stated, all reactions were carried out under an inert N_2 atmosphere. All reagents were purged or sparged with N_2 for 20 min prior to distillation or use. All solid reagents were dried by azeotropic distillation with benzene twice prior to use. Mass spectra were obtained using a Thermo LTqFT mass spectrometer with electrospray ionization and external calibration. Proton and carbon magnetic resonance spectra (^1H NMR and ^{13}C NMR) were recorded on a Bruker model DRX 400, a Bruker model AVANCE III 500, or a

Bruker AVANCE III 600 CryoProbe (^{1}H NMR at 400 MHz, 500 MHz or 600 MHz, ^{13}C NMR at 100 or 151 MHz, ^{31}P NMR at 160 or 243 MHz and ^{19}F NMR at 376 or 564 MHz) spectrometer with solvent resonance as the internal standard (^{1}H NMR: $CDCl_3$ at 7.26 ppm, CD_2Cl_2 at 5.32 ppm, CD_3CN at 1.94 ppm; ^{13}C NMR: $CDCl_3$ at 77.16 ppm, CD_2Cl_2 at 53.84 ppm, CD_3CN at 1.32 ppm). NMR data are reported as follows: chemical shift, integration, multiplicity (s = singlet, d = doublet, t = triplet, dd = doublet of doublets, td = triplet of doublets, dt = doublet of triplets, ddd = doublet of doublet of doublets, m = multiplet, bs = broad singlet, bm = broad multiplet, etc.), and coupling constants (Hz).

The following substrates were prepared according to literature method or a modified literature method and matched reported characterization data: *(E)*-phenyl-1,3-butadiene,[1] *(E)/(Z)*-2-methyl-phenyl-1,3-butadiene,[2] *(E)/(Z)*-3-methyl-phenyl-1,3-butadiene,[2] *(E)/(Z)*-4-methyl-phenyl-1,3-butadiene,[2] *(E)/(Z)*-1-buta-1,3-dien-1-ylcylohexane,[2] *(E)*-4-methoxy-phenyl-1,3-butadiene,[2] *(E)/(Z)*-2-nitro-phenyl-1,3-butadiene,[2] *(E)/(Z)*-4-chloro-phenyl-1,3-butadiene,[3] *(E)/(Z)*-4-fluoro-phenyl-1,3-butadiene,[3] *(E)*-2-(buta-1,3-dien-1-yl)furan,[2] *(E)*-tert-butyl(hexa-3,5-dien-1-yloxy)dimethylsilane,[4] *(E)*-dodeca-1,3-diene,[5] 4-methyl-2-phenyloxazol-5(4H)-one,[6] 2-phenyl-4-propyloxazol-5(4H)-one,[7] 4-isobutyl-2-phenyloxazol-5(4H)-one,[8] 4-phenethyl-2-phenyloxazol-5(4H)-one,[7] 4-allyl-2-phenyloxazol-5(4H)-one,[9] 2-(4-chlorophenyl)-4-methyloxazol-5(4H)-one,[6] sodium tetrakis[3,5-*bis*(trifluoromethyl)phenyl]borate,[10] complex **34**,[11] complex **1**,[12] and (R,R)-TADDOL-P(O)OH.[13]

■ **Solvents:** Solvents were purged with argon and purified under a positive pressure of dry argon by a SG Waters purification system: dichloromethane (EMD Millipore), diethyl ether (EMD Millipore), hexanes (EMD Millipore), benzene (EMD Millipore), and THF (EMD Millipore) were passed through activated alumina columns. $CDCl_3$ and CD_2Cl_2 were purchased from Cambridge Isotope Labs, distilled over CaH_2 and stored in a dry box over activated 4 Å molecular sieves.

■ **Reagents:**

(R)-(+)-1,1'-Bi(2-napthol) was purchased from Chem Impex, dried by azeotropic distillation with benzene, stored in a dry box and used without further purification.

Chloro(1,5-cyclooctadiene)rhodium(I) dimer was purchased from Pressure Chemicals, stored in a dry box and used as received.

(S,S)-1,2-Diphenylethylenediamine was purchased from Ivy Chemicals, dried by azeotropic distillation with benzene, stored in a dry box and used without further purification.

Hexamethyldisiloxane was purchased from Sigma Aldrich, stored over 4Å molecular sieves, and used without further purification.

(S,S)-Hydrobenzoin was purchased from Sigma Aldrich, dried by azeotropic distillation with benzene, stored in a dry box and used without further purification.

Isopropanol was purchased from Fischer Scientific, distilled over CaH_2, stored in a flask over 4Å molecular sieves and sparged with N_2 before use.

Lithium tetrafluoroborate was purchased from Sigma Aldrich, stored in the dry box after overnight heating over P_2O_5 under vacuum and used without further purification.

Lithium hexafluorophosphate was purchased from Sigma Aldrich, stored in the dry box and used as received.

Lithium *tetrakis*(pentafluorophenyl)borate - ethyl ether complex was purchased from Boulder Scientific, stored in a dry box, and used as received.
***m*-Chloroperoxybenzoic acid** was purchased from Alfa-Aesar as 50-55% purity by weight and used as received without further pufication.
Methanol was purchased from Fischer Scientific, distilled over CaH_2, stored in a flask over 4Å molecular sieves and sparged with N_2 before use.
Menthol was purchased from Sigma Aldrich, dried by azeotropic distillation with benzene, stored in a dry box and used without further purification.
Potassium carbonate was purchased from Fischer Scientific and used as received.
Silver chloride was purchased from Strem, stored in a dry box, and used without further purification.
Silver tetrafluoroborate was purchased from Strem, stored in a dry box, and used without further purification.
Sodium methoxide was purchased from Strem, stored in a dry box, and used without further purification.
Styrene was purchased from Alfa Aesar, distilled over CaH_2, and stored at – 20 °C in a dry box.
***t*-Butanol** was purchased from Sigma Aldrich, distilled over CaH_2, stored in a flask over 4Å molecular sieves and melted before use.

■ General procedure for the (CDC)-Rh(I) catalyzed hydroalkylation of dienes with oxazalones in Tables 1, 2 and 3:

In a N_2 filled glove box, an 8 mL reaction vial with a stir bar was charged with (CDC)-Rh(I)styrene $BAr^F{}_4$, the appropriate additive and the listed diene. The appropriate solvent was added by syringe, the reaction vial capped with a Teflon® lined septum cap and the reaction allowed to stir at 22 °C for 10 minutes. The cap was removed and the nucleophile was added directly to the solution as a solid or as a liquid via syringe. The reaction was resealed with the septum cap, the lid secured with electrical tape to ensure a tight seal, and the reaction removed from the glove box. Outside the glove box, a vial of alcohol was sparged for 10 minutes with N_2 and added to the reaction via syringe under an atmosphere of N_2. The reaction was allowed to stir at the appropriate temperature for the listed time before being cooled to room temperature, unsealed, and 5 µL of hexamethyldisiloxane added as an internal standard. The solution was diluted with $CDCl_3$ and analyzed by NMR spectroscopy to determine the conversion and diastereoselectivity. The NMR sample was recombined with the reaction and the solvents removed *in vacuo* before being purified by SiO_2 gel chromatography. Products eluted with similar retention times in the following order: 1) the 1,4-addition products, 2) the *anti*-1,2-addition products, and 3) the *syn*-1,2-addition products.

■ Procedure and characterization for the (CDC)-Rh(I) catalyzed hydroalkylation of dienes with oxazalones in Tables 1, 2 and 3:

Synthesis of 4-methyl-2-phenyl-4-(*E*-4-phenylbut-3-en-2-yl)oxazol-5(4H)-one (4).

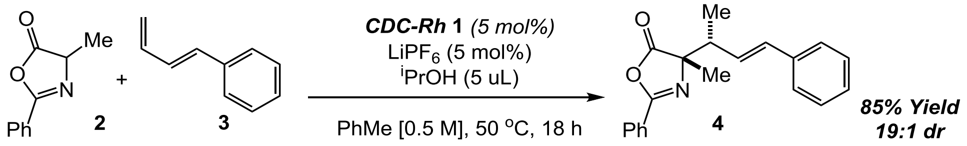

Following the general procedure for the Rh(I) catalyzed hydroalkylation of dienes with oxazalones, (CDC)-Rh(I)styrene $BAr^F{}_4$ **1** (8.1 mg, 0.005 mmol), $LiPF_6$ (0.8 mg, 0.005 mmol), and phenylbutadiene (13.0 mg, 0.100 mmol) were combined in the glove box, solvated with toluene (200 µL, 0.5 M) and allowed to stir at 22 °C for 10 minutes. To this solution, 4-methyl-2-phenyloxazol-5(4H)-one (26.3 mg, 0.150 mmol) was added. The reaction was sealed with a Teflon® septum cap and removed from the glove box. Outside the glove box, N_2 sparged isopropanol (5 µL) was added and the reaction allowed to stir at 50 °C for 18 h. The reaction was cooled to room temperature and 5 µL of hexamethyldisiloxane added as an internal standard. The reaction was diluted with $CDCl_3$ and analyzed by NMR spectroscopy as a 19:1 mixture of the anti:syn diastereomers. The NMR sample was recombined with the reaction and the solvents removed *in vacuo*. The resulting oil was purified by SiO_2 gel column chromatography (20:1 Hex/Et_2O) to afford **4** (26.0 mg, 0.085 mmol, 85% yield, >20:1 dr) as a colorless oil.
^{1}**H NMR** (600 MHz, $CDCl_3$) δ 8.06 – 8.02 (m, 2H), 7.61 – 7.55 (m, 1H), 7.51-7.48 (m, 2H), 7.40 (d, *J* = 7.3 Hz, 2H), 7.31 (t, *J* = 7.6 Hz, 2H), 7.23 (t, *J* = 7.3 Hz, 1H), 6.53 (d, *J* = 15.9 Hz, 1H), 6.25 (dd, *J* = 15.9, 9.3 Hz, 1H), 2.80 (dq, *J* = 13.7, 6.9 Hz, 1H), 1.50 (s, 3H), 1.05 (d, *J* = 6.8 Hz, 3H). ^{13}**C NMR** (151 MHz, $CDCl_3$) δ 181.0, 160.1, 137.0, 132.8, 132.7, 129.3, 128.8, 128.5, 128.0, 127.5, 126.4, 125.9, 72.5, 45.1, 22.7, 15.8. **IR** (ν/cm^{-1}): 3060 (w), 3028 (w), 2973 (m), 2930 (m), 2872 (w), 1821 (s), 1654 (s), 1494 (w), 1450 (m), 1320 (w), 1291 (m), 1173 (m), 1001 (s), 969 (w), 889 (m). **HRMS** (ES^+) $[M–H]^+$ calcd for $C_{20}H_{20}NO_2^+$ 306.1489, found: 306.1488.

Synthesis of 4-(*E*-4-(4-chlorophenyl)but-3-en-2-yl)-4-methyl-2-phenyloxazol-5(4H)-one (5).

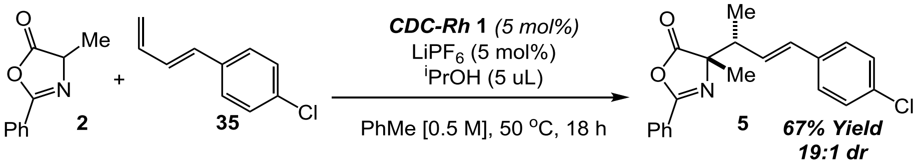

Following the general procedure for the Rh(I) catalyzed hydroalkylation of dienes with oxazalones, (CDC)-Rh(I)styrene $BAr^F{}_4$ **1** (8.1 mg, 0.005 mmol), $LiPF_6$ (0.8 mg, 0.005 mmol), and *p*-chloro-phenylbutadiene (16.5 mg, 0.100 mmol) were combined in the glove box, solvated with toluene (200 µL, 0.5 M) and allowed to stir at 22 °C for 10 minutes. To this solution, 4-methyl-2-phenyloxazol-5(4H)-one (26.3 mg, 0.150 mmol) was added. The reaction was sealed with a Teflon® septum cap and removed from the glove box. Outside the glove box, N_2 sparged isopropanol (5 µL) was added and the reaction allowed to stir at 50 °C for 18 h. The reaction was cooled to room temperature and 5 µL of hexamethyldisiloxane added as an internal standard. The reaction was diluted with $CDCl_3$ and analyzed by NMR spectroscopy as a 19:1 mixture of the anti:syn diastereomers. The NMR sample was recombined with the reaction and the solvents removed *in vacuo*. The resulting oil was purified by SiO_2 gel column chromatography (20:1 Hex/Et_2O) to afford **5** (22.8 mg, 0.067 mmol, 67% yield, 19:1 dr) as a colorless oil. The product was isolated with less than 5% of the inseperable 1,4-addition product.
^{1}**H NMR** (600 MHz, $CDCl_3$) δ 8.05 – 8.02 (m, 2H), 7.61 – 7.56 (m, 1H), 7.50 (t, *J* = 7.8 Hz, 2H), 7.32 (d, *J* = 8.6 Hz, 2H), 7.27 (d, *J* = 8.5 Hz, 2H), 6.49 (d, *J* = 15.9 Hz, 1H), 6.22 (dd, *J* =

15.9, 9.3 Hz, 1H), 2.79 (dq, J = 13.7, 6.8 Hz, 1H), 1.49 (s, 3H), 1.05 (d, J = 6.8 Hz, 3H). **^{13}C NMR** (151 MHz, $CDCl_3$) δ 180.9, 160.4, 135.6, 132.9, 131.7, 130.2, 129.0, 128.8, 128.2, 127.7, 125.9, 72.5, 45.1, 22.8, 15.9. **IR** (ν/cm^{-1}): 2972 (m), 2930 (m), 1820 (s), 1654 (s), 1492 (m), 1451 (m), 1320 (w), 1291 (m), 1173 (m), 1091 (m), 1001 (s), 971 (w), 890 (m). **HRMS** (ES$^+$) [M–H]$^+$ calcd for $C_{20}H_{19}ClNO_2^+$ 340.1099, found: 340.1099.

Synthesis of 4-(*E*-4-(4-fluorophenyl)but-3-en-2-yl)-4-methyl-2-phenyloxazol-5(4H)-one (6).

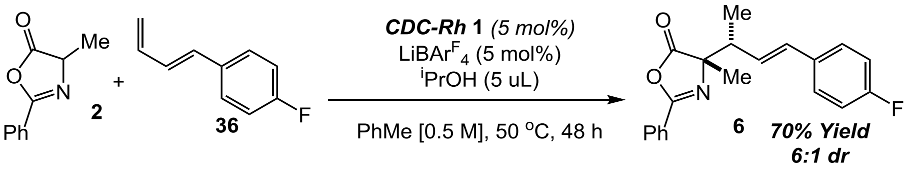

Following the general procedure for the Rh(I) catalyzed hydroalkylation of dienes with oxazalones, (CDC)-Rh(I)styrene BAr$^F_4$ **1** (8.1 mg, 0.005 mmol), LiBAr$^F_4$ (3.4 mg, 0.005 mmol), and p-fluoro-phenylbutadiene (29.6 mg, 0.200 mmol) were combined in the glove box, solvated with toluene (200 µL, 0.5 M) and allowed to stir at 22 °C for 10 minutes. To this solution, 4-methyl-2-phenyloxazol-5(4H)-one (17.5 mg, 0.100 mmol) was added. The reaction was sealed with a Teflon® septum cap and removed from the glove box. Outside the glove box, N_2 sparged isopropanol (5 µL) was added and the reaction allowed to stir at 50 °C for 48 h. The reaction was cooled to room temperature and 5 µL of hexamethyldisiloxane added as an internal standard. The reaction was diluted with $CDCl_3$ and analyzed by NMR spectroscopy as a 9:1 mixture of the anti:syn diastereomers. The NMR sample was recombined with the reaction and the solvents removed *in vacuo*. The resulting oil was purified by SiO_2 gel column chromatography (20:1 Hex/Et_2O) to afford **6** (22.6 mg, 0.070 mmol, 70% yield, 6:1 dr) as a colorless oil. The product was isolated with less than 5% of the inseperable 1,4-addition product.

^{1}H NMR (600 MHz, $CDCl_3$) δ 8.05 – 8.01 (m, 2H), 7.59 (t, J = 7.4 Hz, 1H), 7.50 (t, J = 7.7 Hz, 2H), 7.36 (dd, J = 8.6, 5.5 Hz, 2H), 7.00 (t, J = 8.6 Hz, 2H), 6.49 (d, J = 15.9 Hz, 1H), 6.16 (dd, J = 15.9, 9.3 Hz, 1H), 2.79 (td, J = 13.7, 6.8 Hz, 1H), 1.50 (s, 3H), 1.04 (d, J = 6.8 Hz, 3H). **^{13}C NMR** (151 MHz, $CDCl_3$) δ 180.9, 160.2, 132.7, 131.6 129.0, 128.8, 128.0, 127.9, 127.8, 126.4, 125.9, 115.4 (d, J = 21.5 Hz) 72.4, 45.0, 22.7, 15.8. **IR** (ν/cm^{-1}): 2974 (m), 2930 (m), 1821 (s), 1783 (m), 1654 (s), 1603 (w), 1508 (s), 1451 (m), 1291 (m), 1229 (m), 1158 (m), 1001 (s), 970 (w), 890 (m), 819 (m). **HRMS** (ES$^+$) [M–H]$^+$ calcd for $C_{20}H_{19}FNO_2^+$ 324.1394, found: 324.1395.

Synthesis of 4-(*E*-4-(4-nitrophenyl)but-3-en-2-yl)-4-methyl-2-phenyloxazol-5(4H)-one (7).

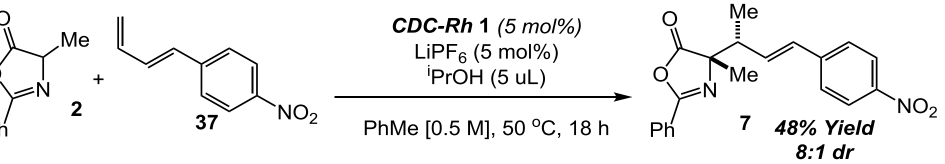

Following the general procedure for the Rh(I) catalyzed hydroalkylation of dienes with oxazalones, (CDC)-Rh(I)styrene BAr$^F_4$ **1** (8.1 mg, 0.005 mmol), $LiPF_6$ (0.8 mg, 0.005 mmol), and *p*-nitro-phenylbutadiene (17.5 mg, 0.100 mmol) were combined in the glove box, solvated

with toluene (200 µL, 0.5 M) and allowed to stir at 22 °C for 10 minutes. To this solution, 4-methyl-2-phenyloxazol-5(4H)-one (26.3 mg, 0.150 mmol) was added. The reaction was sealed with a Teflon® septum cap and removed from the glove box. Outside the glove box, N_2 sparged isopropanol (5 µL) was added and the reaction allowed to stir at 50 °C for 18 h. The reaction was cooled to room temperature and 5 µL of hexamethyldisiloxane added as an internal standard. The reaction was diluted with $CDCl_3$ and analyzed by NMR spectroscopy as a 8:1 mixture of the anti:syn diastereomers. The NMR sample was recombined with the reaction and the solvents removed *in vacuo*. The resulting oil was purified by SiO_2 gel column chromatography (20:1 Hex/Et_2O) to afford **7** (16.8 mg, 0.048 mmol, 48% yield, 8:1 dr) as a light yellow oil.
^{1}H NMR (500 MHz, $CDCl_3$) δ 8.18 (d, *J* = 8.8 Hz, 2H), 8.06 – 8.01 (m, 2H), 7.60 (t, *J* = 7.4 Hz, 1H), 7.54 – 7.48 (m, 4H), 6.61 (d, *J* = 15.9 Hz, 1H), 6.46 (dd, *J* = 15.9, 9.2 Hz, 1H), 2.86 (dq, *J* = 13.7, 6.8 Hz, 1H), 1.51 (s, 3H), 1.08 (d, *J* = 6.8 Hz, 3H). **^{13}C NMR** (151 MHz, $CDCl_3$) δ 180.6, 160.6, 147.1, 143.5, 134.7, 133.0, 131.1, 129.0, 128.2, 127.1, 125.9, 124.2, 72.3, 45.2, 22.8, 15.7. **IR** (ν/cm^{-1}): 3062 (w), 2975 (m), 2932 (m), 2851 (w), 1822 (s), 1653 (s), 1596 (m), 1519 (s), 1456 (m), 1342 (s), 1290 (w), 1174 (m), 1002 (m), 891 (m). **HRMS** (ES$^+$) $[M–H]^+$ calcd for $C_{20}H_{19}N_2O_4^+$ 351.1339, found: 351.1338.

Synthesis of 4-(*E*-4-(4-methoxyphenyl)but-3-en-2-yl)-4-methyl-2-phenyloxazol-5(4H)-one (8).

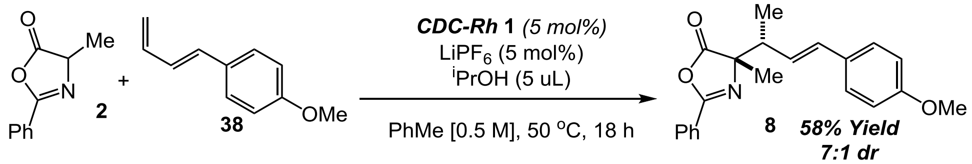

Following the general procedure for the Rh(I) catalyzed hydroalkylation of dienes with oxazalones, (CDC)-Rh(I)styrene BAr^F_4 **1** (8.1 mg, 0.005 mmol), $LiPF_6$ (0.8 mg, 0.005 mmol), and *p*-methoxy-phenylbutadiene (16.0 mg, 0.100 mmol) were combined in the glove box, solvated with toluene (200 µL, 0.5 M) and allowed to stir at 22 °C for 10 minutes. To this solution, 4-methyl-2-phenyloxazol-5(4H)-one (26.3 mg, 0.150 mmol) was added. The reaction was sealed with a Teflon® septum cap and removed from the glove box. Outside the glove box, N_2 sparged isopropanol (5 µL) was added and the reaction allowed to stir at 50 °C for 18 h. The reaction was cooled to room temperature and 5 µL of hexamethyldisiloxane added as an internal standard. The reaction was diluted with $CDCl_3$ and analyzed by NMR spectroscopy as a 4:1 mixture of the anti:syn diastereomers. The NMR sample was recombined with the reaction and the solvents removed *in vacuo*. The resulting oil was purified by SiO_2 gel column chromatography (20:1 Hex/Et_2O) to afford **8** (19.4 mg, 0.058 mmol, 58% yield, 7:1 dr) as a colorless oil.
***anti*-Diastereomer (major):** [**^{1}H NMR** (600 MHz, $CDCl_3$) δ 8.03 (d, *J* = 7.2 Hz, 2H), 7.58 (t, *J* = 7.4 Hz, 1H), 7.49 (t, *J* = 7.6 Hz, 2H), 7.33 (d, *J* = 8.5 Hz, 2H), 6.84 (d, *J* = 8.6 Hz, 2H), 6.47 (d, *J* = 15.8 Hz, 1H), 6.09 (dd, *J* = 15.8, 9.3 Hz, 1H), 3.80 (s, 3H), 2.82 – 2.72 (m, 1H), 1.49 (s, 3H), 1.03 (d, *J* = 6.8 Hz, 3H). **^{13}C NMR** (151 MHz, $CDCl_3$) δ 181.1, 160.1, 159.1, 132.7, 132.2,

129.8, 128.8, 128.0, 127.5, 127.0, 125.9, 113.9, 72.5, 55.3, 45.1, 22.7, 15.9.] ***syn*-Diastereomer (minor):** [**^{1}H NMR** (600 MHz, $CDCl_3$) δ 8.01 (d, *J* = 7.2 Hz, 2H), 7.59 – 7.53 (m, 1H), 7.38 (t, *J* = 7.2 Hz, 2H), 7.21 (d, *J* = 8.5 Hz, 2H), 6.82 (d, *J* = 8.7 Hz, 2H), 6.28 (d, *J* = 15.9 Hz, 1H), 5.73 (dd, *J* = 16.0, 8.4 Hz, 1H), 3.80 (s, *J* = 6.8 Hz, 3H), 3.12 – 3.07 (m, 1H), 2.22 (s, 3H), 1.07 (d, *J* = 6.9 Hz, 3H).] **IR** (ν/cm^{-1}): 3062 (w), 3033 (w), 2972 (m), 2933 (m), 2836 (m), 1820 (s), 1782 (m), 1654 (s), 1607 (m), 1511 (s), 1450 (m), 1297 (m), 1250 (s), 1175 (m), 1033 (m), 1001 (s), 969 (m), 889 (m). **HRMS** (ES^+) $[M–H]^+$ calcd for $C_{21}H_{22}NO_3^+$ 336.1594, found: 336.1593.

Synthesis of 4-methyl-2-phenyl-4-(*E*-4-(o-tolyl)but-3-en-2-yl)oxazol-5(4H)-one (9).

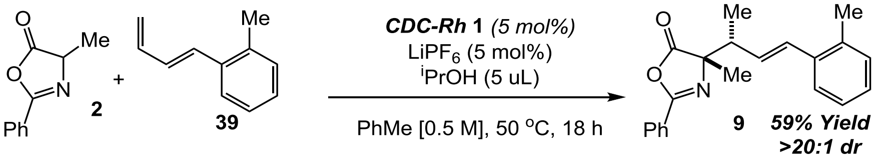

Following the general procedure for the Rh(I) catalyzed hydroalkylation of dienes with oxazalones, (CDC)-Rh(I)styrene BAr^F_4 **1** (8.1 mg, 0.005 mmol), $LiPF_6$ (0.8 mg, 0.005 mmol), and *o*-methyl-phenylbutadiene (14.4 mg, 0.100 mmol) were combined in the glove box, solvated with toluene (200 µL, 0.5 M) and allowed to stir at 22 °C for 10 minutes. To this solution, 4-methyl-2-phenyloxazol-5(4H)-one (26.3 mg, 0.150 mmol) was added. The reaction was sealed with a Teflon® septum cap and removed from the glove box. Outside the glove box, N_2 sparged isopropanol (5 µL) was added and the reaction allowed to stir at 50 °C for 18 h. The reaction was cooled to room temperature and 5 µL of hexamethyldisiloxane added as an internal standard. The reaction was diluted with $CDCl_3$ and analyzed by NMR spectroscopy as a >20:1 mixture of the anti:syn diastereomers. The NMR sample was recombined with the reaction and the solvents removed *in vacuo*. The resulting oil was purified by SiO_2 gel column chromatography (20:1 Hex/Et_2O) to afford **9** (18.8 mg, 0.059 mmol, 59% yield, >20:1 dr) as a colorless oil.

^{1}H NMR (400 MHz, $CDCl_3$) δ 8.04 – 8.02 (m, 2H), 7.62 – 7.53 (m, 1H), 7.51 – 7.47 (m, 2H), 7.46 – 7.39 (m, 1H), 7.20 – 7.04 (m, 3H), 6.74 (d, *J* = 15.7 Hz, 1H), 6.08 (dd, *J* = 15.7, 9.3 Hz, 1H), 2.83 (dq, *J* = 13.7, 6.9 Hz, 1H), 2.32 (s, 3H), 1.52 (s, 3H), 1.09 (d, *J* = 6.8 Hz, 3H). **^{13}C NMR** (100 MHz, $CDCl_3$) δ 181.0, 160.2, 136.4, 135.4, 132.8, 131.0, 130.9, 130.3, 128.9, 128.1, 127.6, 126.2, 126.1, 126.0, 72.5, 45.4, 22.8, 19.9, 15.9. **IR** (ν/cm^{-1}): 3062 (w), 3022 (w), 2973 (m), 2930 (m), 2872 (w), 1821 (s), 1653 (s), 1451 (m), 1320 (w), 1291 (m), 1173 (m), 1001 (s), 970 (w), 889 (m). **HRMS** (ES^+) $[M–H]^+$ calcd for $C_{21}H_{22}NO_2^+$ 320.1645, found: 320.1645.

Synthesis of 4-methyl-2-phenyl-4-(*E*-4-(m-tolyl)but-3-en-2-yl)oxazol-5(4H)-one (10).

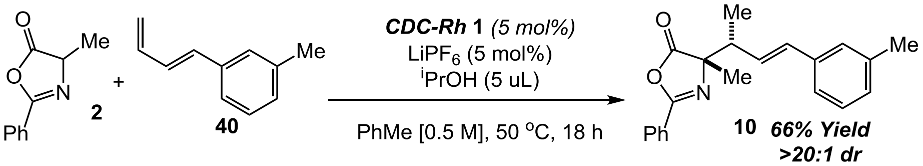

Following the general procedure for the Rh(I) catalyzed hydroalkylation of dienes with oxazalones, (CDC)-Rh(I)styrene BAr^F_4 **1** (8.1 mg, 0.005 mmol), $LiPF_6$ (0.8 mg, 0.005 mmol),

and *m*-methyl-phenylbutadiene (14.4 mg, 0.100 mmol) were combined in the glove box, solvated with toluene (200 µL, 0.5 M) and allowed to stir at 22 °C for 10 minutes. To this solution, 4-methyl-2-phenyloxazol-5(4H)-one (26.3 mg, 0.150 mmol) was added. The reaction was sealed with a Teflon® septum cap and removed from the glove box. Outside the glove box, N_2 sparged isopropanol (5 µL) was added and the reaction allowed to stir at 50 °C for 18 h. The reaction was cooled to room temperature and 5 µL of hexamethyldisiloxane added as an internal standard. The reaction was diluted with $CDCl_3$ and analyzed by NMR spectroscopy as a >20:1 mixture of the anti:syn diastereomers. The NMR sample was recombined with the reaction and the solvents removed *in vacuo*. The resulting oil was purified by SiO_2 gel column chromatography (20:1 Hex/Et_2O) to afford **10** (21.1 mg, 0.066 mmol, 66% yield, 20:1 dr) as a colorless oil.
^{1}H NMR (600 MHz, $CDCl_3$) δ 8.07 – 7.98 (m, 2H), 7.58 (t, *J* = 7.4 Hz, 1H), 7.50 (t, *J* = 7.7 Hz, 2H), 7.23-7.20 (m, 3H), 7.05 – 7.04 (m, 1H), 6.50 (d, *J* = 15.8 Hz, 1H), 6.23 (dd, *J* = 15.8, 9.3 Hz, 1H), 2.81 – 2.77 (m, 1H), 2.35 (s, 3H), 1.50 (s, 3H), 1.04 (d, *J* = 6.8 Hz, 3H). **^{13}C NMR** (151 MHz, $CDCl_3$) δ 181.0, 160.1, 138.1, 136.9, 132.9, 132.7, 129.1, 128.8, 128.4, 128.3, 128.0, 127.0, 125.9, 123.61, 72.5, 45.1, 22.7, 21.4, 15.9. **IR** (ν/cm^{-1}): 3060 (w), 3030 (m), 2974 (m), 2930 (m), 2872 (w), 1821 (s), 1653 (s), 1494 (m), 1451 (m), 1375 (m), 1292 (m), 1174 (m), 1093 (m), 1001 (s), 970 (w), 888 (s). **HRMS** (ES$^+$) $[M–H]^+$ calcd for $C_{21}H_{22}NO_2^+$ 320.1645, found: 320.1644.

Synthesis of 4-methyl-2-phenyl-4-(*E*-4-(p-tolyl)but-3-en-2-yl)oxazol-5(4H)-one (11).

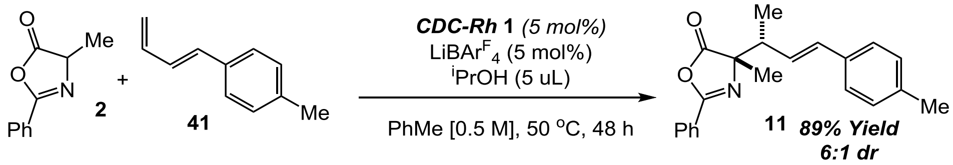

Following the general procedure for the Rh(I) catalyzed hydroalkylation of dienes with oxazalones, (CDC)-Rh(I)styrene BAr^F_4 **1** (8.1 mg, 0.005 mmol), $LiBAr^F_4$ (3.4 mg, 0.005 mmol), and *p*-methyl-phenylbutadiene (28.8 mg, 0.200 mmol) were combined in the glove box, solvated with toluene (200 µL, 0.5 M) and allowed to stir at 22 °C for 10 minutes. To this solution, 4-methyl-2-phenyloxazol-5(4H)-one (17.5 mg, 0.100 mmol) was added. The reaction was sealed with a Teflon® septum cap and removed from the glove box. Outside the glove box, N_2 sparged isopropanol (5 µL) was added and the reaction allowed to stir at 50 °C for 48 h. The reaction was cooled to room temperature and 5 µL of hexamethyldisiloxane added as an internal standard. The reaction was diluted with $CDCl_3$ and analyzed by NMR spectroscopy as a 6:1 mixture of the anti:syn diastereomers. The NMR sample was recombined with the reaction and the solvents removed *in vacuo*. The resulting oil was purified by SiO_2 gel column chromatography (20:1 Hex/Et_2O) to afford **11** (28.4 mg, 0.089 mmol, 89% yield, 6:1 dr) as a colorless oil. The product was isolated with 5% of the inseperable 1,4-addition product.
***anti*-Diastereomer (major):** [**^{1}H NMR** (500 MHz, $CDCl_3$) δ 8.05 – 8.02 (m, 2H), 7.59 – 7.56 (m, 1H), 7.51 – 7.48 (m, 2H), 7.29 (d, *J* = 8.0 Hz, 2H), 7.12 (d, *J* = 7.9 Hz, 2H), 6.50 (d, *J* = 15.8 Hz, 1H), 6.18 (dd, *J* = 15.8, 9.3 Hz, 1H), 2.83 – 2.72 (m, 1H), 2.33 (m, 3H), 1.49 (s, 3H), 1.03 (d,

J = 6.8 Hz, 3H).] ***syn*-Diastereomer (minor):** [^{1}**H NMR** (500 MHz, $CDCl_3$) δ 7.55 (d, J = 7.1 Hz, 2H), 7.41 – 7.32 (m, 3H), 7.17 (d, J = 8.0 Hz, 2H), 7.09 (d, J = 7.9 Hz, 2H), 6.30 (d, J = 15.9 Hz, 1H), 5.82 (dd, J = 15.9, 8.4 Hz, 1H), 3.15 – 3.06 (m, 1H), 2.32 (s, 3H), 2.22 (s, 3H), 1.07 (d, J = 6.9 Hz, 3H).] ^{13}**C NMR** (151 MHz, $CDCl_3$) δ 181.2, 165.6, 160.2, 159.9, 137.7, 137.6, 137.5, 134.3, 134.2, 134.0, 132.8, 132.8, 129.4, 129.4, 128.9, 128.8, 128.4, 128.3, 128.1, 126.5, 126.4, 126.3, 126.2, 126.1, 109.3, 72.6, 47.0, 45.3, 22.8, 21.3, 16.0, 15.3, 14.0. **IR** (ν/cm^{-1}): 3026 (w), 2974 (m), 2930 (m), 2873 (w), 1820 (s), 1783 (m), 1653 (s), 1513 (m), 1451 (m), 1291 (m), 1173 (m), 1001 (s), 971 (m), 889 (m). **HRMS** (ES^+) $[M–H]^+$ calcd for $C_{21}H_{22}NO_2^+$ 320.1645, found: 320.1646.

Synthesis of 4-(*E*-4-(furan-2-yl)but-3-en-2-yl)-4-methyl-2-phenyloxazol-5(4H)-one (12).

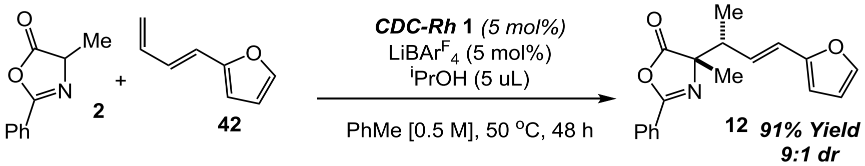

Following the general procedure for the Rh(I) catalyzed hydroalkylation of dienes with oxazalones, (CDC)-Rh(I)styrene BAr^F_4 **1** (8.1 mg, 0.005 mmol), $LiBAr^F_4$ (3.4 mg, 0.005 mmol), and 2-(buta-1,3-dien-1-yl)furan (24.0 mg, 0.200 mmol) were combined in the glove box, solvated with toluene (200 µL, 0.5 M) and allowed to stir at 22 °C for 10 minutes. To this solution, 4-methyl-2-phenyloxazol-5(4H)-one (17.5 mg, 0.100 mmol) was added. The reaction was sealed with a Teflon® septum cap and removed from the glove box. Outside the glove box, N_2 sparged isopropanol (5 µL) was added and the reaction allowed to stir at 50 °C for 48 h. The reaction was cooled to room temperature and 5 µL of hexamethyldisiloxane added as an internal standard. The reaction was diluted with $CDCl_3$ and analyzed by NMR spectroscopy as a 9:1 mixture of the anti:syn diastereomers. The NMR sample was recombined with the reaction and the solvents removed *in vacuo*. The resulting oil was purified by SiO_2 gel column chromatography (20:1 Hex/Et_2O) to afford **12** (26.9 mg, 0.091 mmol, 91% yield, 9:1 dr) as a colorless oil.

^{1}**H NMR** (600 MHz, $CDCl_3$) δ 8.07 – 8.01 (m, 2H), 7.61 – 7.56 (m, 1H), 7.53 – 7.46 (m, 2H), 7.38 – 7.32 (m, 1H), 6.39 – 6.33 (m, 2H), 6.23 (d, J = 3.3 Hz, 1H), 6.20 (dd, J = 16.0, 9.4 Hz, 1H), 2.77 – 2.72 (m, 1H), 1.50 (s, 3H), 1.03 (d, J = 6.8 Hz, 3H). ^{13}**C NMR** (151 MHz, $CDCl_3$) δ 181.1, 160.3, 152.6, 142.0, 132.8, 128.9, 128.2, 128.2, 126.0, 121.3, 111.4, 107.7, 72.6, 44.9, 22.8, 15.9. **IR** (ν/cm^{-1}): 3062 (w), 2976 (m), 2933 (w), 2874 (w), 1820 (s), 1655 (s), 1451 (m), 1291 (m), 1173 (m), 1002 (s), 887 (m). **HRMS** (ES^+) $[M–H]^+$ calcd for $C_{18}H_{19}NO_3^+$ 296.1287, found: 296.1282.

Synthesis of 4-(*E*-dodec-3-en-2-yl)-4-methyl-2-phenyloxazol-5(4H)-one (14).

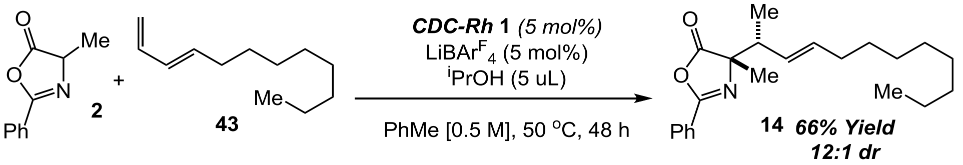

Following the general procedure for the Rh(I) catalyzed hydroalkylation of dienes with oxazalones, (CDC)-Rh(I)styrene $BAr^F{}_4$ **1** (8.1 mg, 0.005 mmol), $LiBAr^F{}_4$ (3.4 mg, 0.005 mmol), and 1,3-dodecadiene (33.3 mg, 0.200 mmol) were combined in the glove box, solvated with toluene (200 µL, 0.5 M) and allowed to stir at 22 °C for 10 minutes. To this solution, 4-methyl-2-phenyloxazol-5(4H)-one (17.5 mg, 0.100 mmol) was added. The reaction was sealed with a Teflon® septum cap and removed from the glove box. Outside the glove box, N_2 sparged isopropanol (5 µL) was added and the reaction allowed to stir at 50 °C for 48 h. The reaction was cooled to room temperature and 5 µL of hexamethyldisiloxane added as an internal standard. The reaction was diluted with $CDCl_3$ and analyzed by NMR spectroscopy as a 12:1 mixture of the anti:syn diastereomers. The NMR sample was recombined with the reaction and the solvents removed *in vacuo*. The resulting oil was purified by SiO_2 gel column chromatography (100% Hex to 20:1 Hex/Et_2O) to afford **14** (22.5 mg, 0.066 mmol, 66% yield, 12:1 dr) as a colorless oil. **^{1}H NMR** (600 MHz, $CDCl_3$) δ 8.05 – 8.00 (m, 2H), 7.61 – 7.54 (m, 1H), 7.50 – 7.47 (m, 2H), 5.58 (dt, *J* = 13.8, 7.9 Hz, 1H), 5.40 (dd, *J* = 15.3, 9.1 Hz, 1H), 2.58 (dq, *J* = 13.8, 6.9 Hz, 1H), 2.02 – 1.99 (m, 2H), 1.47 (s, 3H), 1.34 – 1.17 (m, 12H), 0.98 (d, *J* = 6.9 Hz, 3H), 0.88 (t, *J* = 7.1 Hz, 3H). **^{13}C NMR** (151 MHz, $CDCl_3$) δ 181.0, 159.8, 134.4, 132.6, 129.0, 128.7, 127.9, 126.0, 72.3, 44.6, 32.6, 31.9, 29.4, 29.4, 29.3, 29.1, 22.7, 22.4, 15.6, 14.1. **IR** (ν/cm^{-1}): 3063 (w), 3033 (w), 2957 (w), 2926 (s), 2854 (m), 1822 (s), 1654 (s), 1452 (m), 1321 (w), 1292 (m), 1175 (m), 1000 (s), 972 (w), 886 (m). **HRMS** (ES^+) $[M–H]^+$ calcd for $C_{22}H_{32}NO_2^+$ 342.2428, found: 342.2428.

Synthesis of 4-(*E*-4-cyclohexylbut-3-en-2-yl)-4-methyl-2-phenyloxazol-5(4H)-one (15).

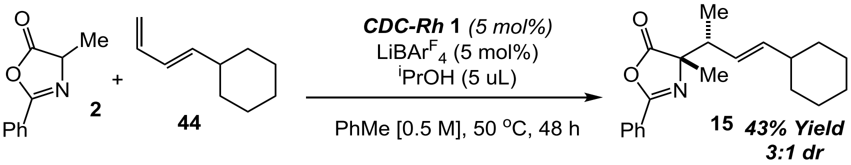

Following the general procedure for the Rh(I) catalyzed hydroalkylation of dienes with oxazalones, (CDC)-Rh(I)styrene $BAr^F{}_4$ **1** (8.1 mg, 0.005 mmol), $LiBAr^F{}_4$ (3.4 mg, 0.005 mmol), and cyclohexylbutadiene (27.2 mg, 0.200 mmol) were combined in the glove box, solvated with toluene (200 µL, 0.5 M) and allowed to stir at 22 °C for 10 minutes. To this solution, 4-methyl-2-phenyloxazol-5(4H)-one (17.5 mg, 0.100 mmol) was added. The reaction was sealed with a Teflon® septum cap and removed from the glove box. Outside the glove box, N_2 sparged isopropanol (5 µL) was added and the reaction allowed to stir at 50 °C for 48 h. The reaction was cooled to room temperature and 5 µL of hexamethyldisiloxane added as an internal standard. The reaction was diluted with $CDCl_3$ and analyzed by NMR spectroscopy as a 3:1 mixture of the anti:syn diastereomers. The NMR sample was recombined with the reaction and the solvents

removed *in vacuo*. The resulting oil was purified by SiO_2 gel column chromatography (100% Hex to 20:1 Hex/Et_2O) to afford **15** (13.3 mg, 0.043 mmol, 43% yield, 6:1 dr) as a colorless oil.

***anti*-Diastereomer (major):** [**^{1}H NMR** (600 MHz, $CDCl_3$) δ 8.00 – 7.98 (m, 2H), 7.58 – 7.54 (m, 1H), 7.50 – 7.45 (m, 2H), 5.51 (dd, *J* = 15.0, 6.9 Hz, 1H), 5.33 (ddd, *J* = 15.4, 9.1, 1.1 Hz, 1H), 2.57 – 2.51 (m, 1H), 1.95 – 1.90 (m, 1H), 1.67 – 1.56 (m, 6H), 1.45 (s, 3H), 1.28 – 1.13 (m, 2H), 1.07 – 0.98 (m, 2H), 0.96 (d, *J* = 6.8 Hz, 3H). **^{13}C NMR** (151 MHz, $CDCl_3$) δ 181.2, 159.9, 140.4, 132.7, 128.9, 128.1, 128.0, 126.6, 126.2, 72.5, 44.8, 40.8, 33.2, 33.1, 26.3, 26.1, 22.4, 15.8.] ***syn*-Diastereomer (minor):** [**^{1}H NMR** (600 MHz, $CDCl_3$) δ 8.01 – 7.98 (m, 2H), 7.58 – 7.54 (m, 1H), 7.50 – 7.46 (m, 2H), 5.48 (dd, *J* = 14.5, 6.9 Hz, 1H), 5.23 (ddd, *J* = 15.4, 9.0, 1.1 Hz, 1H), 2.57 – 2.51 (m, 1H), 1.88 – 1.81 (m, 1H), 1.69 – 1.54 (m, 6H), 1.47 (s, 3H), 1.27 – 1.13 (m, 2H), 1.11 (d, *J* = 6.9 Hz, 3H), 1.09 – 0.98 (m, 2H). **^{13}C NMR** (151 MHz, $CDCl_3$) δ 180.7, 159.9, 140.1, 132.7, 128.9, 128.1, 128.0, 126.4, 126.2, 73.1, 44.5, 40.6, 33.2, 33.0, 26.2, 26.0, 22.1, 15.1.] **IR** (ν/cm^{-1}): 2973 (w), 2925 (s), 2851 (m), 1822 (s), 1653 (s), 1508 (s), 1457 (m), 1291 (m), 1228 (m), 1158 (m), 1001 (s), 890 (m). **HRMS** (ES^+) $[M–H]^+$ calcd for $C_{20}H_{25}NO_2^+$ 312.1964, found: 312.1958.

Synthesis of 4-(*E*-6-((tert-butyldimethylsilyl)oxy)hex-3-en-2-yl)-4-methyl-2-phenyloxazol-5(4H)-one (16).

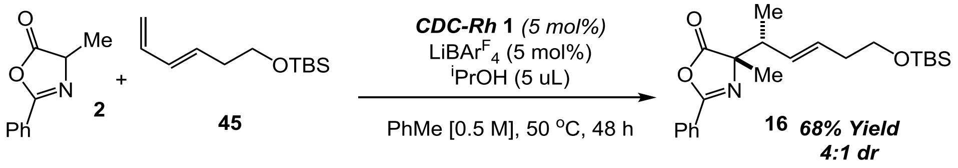

Following the general procedure for the Rh(I) catalyzed hydroalkylation of dienes with oxazalones, (CDC)-Rh(I)styrene BAr$^F_4$ **1** (8.1 mg, 0.005 mmol), LiBAr$^F_4$ (3.4 mg, 0.005 mmol), and *tert*-butyl(hexa-3,5-dien-1-yloxy)dimethylsilane (42.5 mg, 0.200 mmol) were combined in the glove box, solvated with toluene (200 µL, 0.5 M) and allowed to stir at 22 °C for 10 minutes. To this solution, 4-methyl-2-phenyloxazol-5(4H)-one (17.5 mg, 0.100 mmol) was added. The reaction was sealed with a Teflon® septum cap and removed from the glove box. Outside the glove box, N_2 sparged isopropanol (5 µL) was added and the reaction allowed to stir at 50 °C for 48 h. The reaction was cooled to room temperature and 5 µL of hexamethyldisiloxane added as an internal standard. The reaction was diluted with $CDCl_3$ and analyzed by NMR spectroscopy as a 4:1 mixture of the anti:syn diastereomers. The NMR sample was recombined with the reaction and the solvents removed *in vacuo*. The resulting oil was purified by SiO_2 gel column chromatography (100% Hex to 20:1 Hex/Et_2O) to afford **16** (26.4 mg, 0.068 mmol, 68% yield, 4:1 dr) as a colorless oil.

***anti*-Diastereomer (major):** [**^{1}H NMR** (600 MHz, $CDCl_3$) δ 8.02 – 7.99 (m, 2H), 7.60 – 7.53 (m, 1H), 7.51 – 7.46 (m, 2H), 5.64 – 5.56 (m, 1H), 5.48 (dd, *J* = 15.4, 9.0 Hz, 1H), 3.58 (t, *J* = 6.8 Hz, 2H), 2.68 – 2.50 (m, 1H), 2.30 – 2.20 (m, 2H), 1.46 (s, 3H), 0.96 (d, *J* = 6.9 Hz, 3H), 0.88 (s, 9H), 0.03 (s, 6H). **^{13}C NMR** (151 MHz, $CDCl_3$) δ 181.2, 160.0, 132.8, 131.2, 130.6, 128.9, 128.1, 126.1, 72.4, 63.1, 44.8, 36.3, 26.1, 22.6, 18.5, 15.8, -5.1.] ***syn*-Diastereomer**

(minor): [^{1}**H NMR** (600 MHz, $CDCl_3$) δ 8.01 – 7.98 (m, 2H), 7.60 – 7.53 (m, 1H), 7.51 – 7.46 (m, 2H), 5.61 – 5.53 (m, 1H), 5.38 (dd, *J* = 15.4, 9.0 Hz, 1H), 3.56 – 3.52 (m, 2H), 2.68 – 2.50 (m, 1H), 2.19 – 2.15 (m, 2H), 1.48 (s, 3H), 1.11 (d, *J* = 6.9 Hz, 3H), 0.85 (s, 9H), 0.00 (s, 3H), 0.00 (s, 3H).] **IR** (ν/cm^{-1}): 2954 (m), 2929 (s), 2857 (m), 1823 (s), 1653 (s), 1452 (m), 1292 (m), 1255 (m), 1174 (m), 1099 (s), 1002 (s), 886 (m). **HRMS** (ES$^+$) [M–H]$^+$ calcd for $C_{22}H_{34}NO_{3Si}^+$ 388.2308, found: 388.2302.

Synthesis of 4-(*E*-4-(furan-2-yl)but-3-en-2-yl)-2-phenyl-4-propyloxazol-5(4H)-one (17).

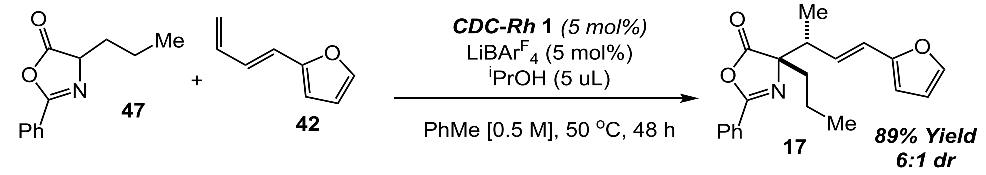

Following the general procedure for the Rh(I) catalyzed hydroalkylation of dienes with oxazalones, (CDC)-Rh(I)styrene BAr$^F_4$ **1** (8.1 mg, 0.005 mmol), LiBAr$^F_4$ (3.4 mg, 0.005 mmol), and 2-(buta-1,3-dien-1-yl)furan (12.0 mg, 0.100 mmol) were combined in the glove box, solvated with toluene (200 µL, 0.5 M) and allowed to stir at 22 °C for 10 minutes. To this solution, 2-phenyl-4-propyloxazol-5(4H)-one (30.5 mg, 0.150 mmol) was added. The reaction was sealed with a Teflon® septum cap and removed from the glove box. Outside the glove box, N_2 sparged isopropanol (5 µL) was added and the reaction allowed to stir at 50 °C for 48 h. The reaction was cooled to room temperature and 5 µL of hexamethyldisiloxane added as an internal standard. The reaction was diluted with $CDCl_3$ and analyzed by NMR spectroscopy as a 6:1 mixture of the anti:syn diastereomers. The NMR sample was recombined with the reaction and the solvents removed *in vacuo*. The resulting oil was purified by SiO_2 gel column chromatography (40:1 Hex/Et_2O) to afford **17** (28.8 mg, 0.089 mmol, 89% yield, 6:1 dr) as a colorless oil.

^{1}**H NMR** (600 MHz, $CDCl_3$) δ 8.05 – 8.03 (m, 2H), 7.60 – 7.56 (m, 1H), 7.52 – 7.48 (m, 2H), 7.34 (d, *J* = 1.5 Hz, 1H), 6.37 (dd, *J* = 3.3, 1.8 Hz, 1H), 6.34 (d, *J* = 15.9 Hz, 1H), 6.24 – 6.18 (m, 2H), 2.76 (dq, *J* = 9.1, 6.8 Hz, 1H), 2.00 – 1.92 (m, 1H), 1.84 – 1.76 (m, 1H), 1.27 – 1.16 (m, 1H), 1.16 – 1.07 (m, 1H), 1.01 (d, *J* = 6.8 Hz, 3H), 0.86 (t, *J* = 7.3 Hz, 3H). ^{13}**C NMR** (151 MHz, $CDCl_3$) δ 180.7, 160.4, 152.6, 141.9, 132.8, 128.9, 128.5, 128.2, 125.9, 120.9, 111.4, 107.6, 76.9, 44.7, 38.3, 17.4, 16.0, 14.0. **IR** (ν/cm^{-1}): 3446 (br, w), 2964 (s), 2933 (m), 2874 (m), 1811 (s), 1653 (s), 1493 (m), 1452 (m), 1320 (m), 1293 (m), 1163 (m), 1020 (m), 944 (m), 883 (m). **HRMS** (ES$^+$) [M–H]$^+$ calcd for $C_{20}H_{22}NO_3^+$ 324.1600, found: 324.1610.

Synthesis of 4-(*E*-dodec-3-en-2-yl)-2-phenyl-4-propyloxazol-5(4H)-one (18).

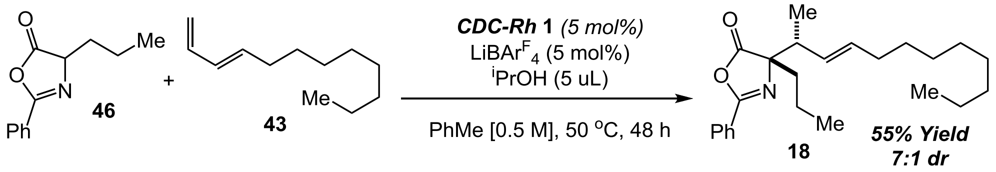

Following the general procedure for the Rh(I) catalyzed hydroalkylation of dienes with oxazalones, (CDC)-Rh(I)styrene BAr^F_4 **1** (8.1 mg, 0.005 mmol), $LiBAr^F_4$ (3.4 mg, 0.005 mmol), and 1,3-dodecadiene (16.6 mg, 0.100 mmol) were combined in the glove box, solvated with toluene (200 µL, 0.5 M) and allowed to stir at 22 °C for 10 minutes. To this solution, 2-phenyl-4-propyloxazol-5(4H)-one (30.5 mg, 0.150 mmol) was added. The reaction was sealed with a Teflon® septum cap and removed from the glove box. Outside the glove box, N_2 sparged isopropanol (5 µL) was added and the reaction allowed to stir at 50 °C for 48 h. The reaction was cooled to room temperature and 5 µL of hexamethyldisiloxane added as an internal standard. The reaction was diluted with $CDCl_3$ and analyzed by NMR spectroscopy as a 7:1 mixture of the anti:syn diastereomers. The NMR sample was recombined with the reaction and the solvents removed *in vacuo*. The resulting oil was purified by SiO_2 gel column chromatography (100% Hex to 40:1 Hex/Et_2O) to afford **18** (20.3 mg, 0.055 mmol, 55% yield, 7:1 dr) as a colorless oil.
^{1}H NMR (600 MHz, $CDCl_3$) δ 8.04 – 7.98 (m, 2H), 7.61 – 7.53 (m, 1H), 7.50 – 7.47 (m, *J* = 7.5, 4.1, 2.5 Hz, 2H), 5.59 – 5.50 (m, 1H), 5.42 – 5.37 (m, 1H), 2.65 – 2.53 (m, 1H), 2.02 – 1.98 (m, 2H), 1.95 – 1.88 (m, 1H), 1.78 (ddd, *J* = 13.7, 12.1, 4.8 Hz, 1H), 1.35 – 1.16 (m, 13H), 1.17 – 1.05 (m, 1H), 0.95 (d, *J* = 6.9 Hz, 3H), 0.87 (t, *J* = 7.2 Hz, 6H). **^{13}C NMR** (151 MHz, $CDCl_3$) δ 180.8, 160.1, 134.1, 132.7, 129.4, 128.9, 128.1, 126.1, 76.7, 44.5, 38.1, 32.7, 32.0, 29.6, 29.5, 29.4, 29.3, 22.8, 17.5, 15.9, 14.3, 14.1. **IR** (ν/cm^{-1}): 2960 (m), 2926 (s), 2873 (w), 2854 (m), 1812 (s), 1654 (s), 1452 (m), 1321 (m), 1293 (m), 1165 (w), 1040 (m), 1020 (m), 942 (m), 881 (m). **HRMS** (ES$^+$) [M–H]$^+$ calcd for $C_{24}H_{36}NO_2^+$ 370.2746, found: 370.2751.

Synthesis of 2-phenyl-4-propyl-4-(*E*-4-(o-tolyl)but-3-en-2-yl)oxazol-5(4H)-one (19).

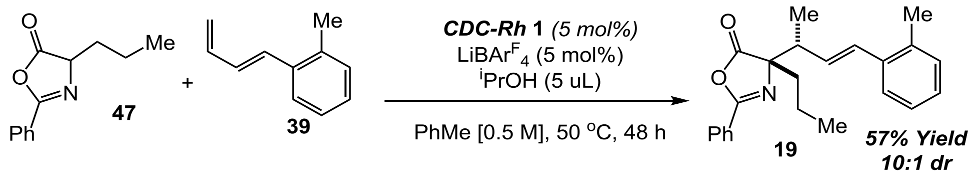

Following the general procedure for the Rh(I) catalyzed hydroalkylation of dienes with oxazalones, (CDC)-Rh(I)styrene BAr^F_4 **1** (8.1 mg, 0.005 mmol), $LiBAr^F_4$ (3.4 mg, 0.005 mmol), and *o*-methyl-phenylbutadiene (14.4 mg, 0.100 mmol) were combined in the glove box, solvated with toluene (200 µL, 0.5 M) and allowed to stir at 22 °C for 10 minutes. To this solution, 2-phenyl-4-propyloxazol-5(4H)-one (30.5 mg, 0.150 mmol) was added. The reaction was sealed with a Teflon® septum cap and removed from the glove box. Outside the glove box, N_2 sparged isopropanol (5 µL) was added and the reaction allowed to stir at 50 °C for 48 h. The reaction was cooled to room temperature and 5 µL of hexamethyldisiloxane added as an internal standard. The reaction was diluted with $CDCl_3$ and analyzed by NMR spectroscopy as a 10:1 mixture of the anti:syn diastereomers. The NMR sample was recombined with the reaction and the solvents removed *in vacuo*. The resulting oil was purified by SiO_2 gel column chromatography (40:1 Hex/Et_2O) to afford **19** (19.8 mg, 0.057 mmol, 57% yield, 10:1 dr) as a colorless oil. The product was isolated with less than 5% of the inseperable 1,4-addition product.

^{1}H NMR (500 MHz, $CDCl_3$) δ 8.06 – 8.02 (m, 2H), 7.61 – 7.55 (m, 1H), 7.52 – 7.47 (m, 2H), 7.48 – 7.43 (m, 1H), 7.20 – 7.12 (m, 3H), 6.72 (d, *J* = 15.7 Hz, 1H), 6.10 (dd, *J* = 15.7, 9.4 Hz, 1H), 2.85 (dq, *J* = 9.1, 6.8 Hz, 1H), 2.61 – 2.47 (m, 1H), 2.32 (s, 3H), 1.96 (ddd, *J* = 13.5, 12.4, 4.6 Hz, 1H), 1.85 (ddd, *J* = 13.7, 12.1, 4.9 Hz, 1H), 1.77 – 1.63 (m, 1H), 1.29 – 1.10 (m, 2H), 1.06 (d, *J* = 6.8 Hz, 3H), 0.87 (t, *J* = 7.3 Hz, 3H). **^{13}C NMR** (151 MHz, $CDCl_3$) δ 180.71, 160.31, 136.32, 135.38, 132.78, 131.14, 130.50, 130.34, 128.92, 128.12, 127.52, 126.19, 125.95, 125.93, 76.75, 45.16, 38.34, 19.94, 17.44, 16.00, 14.04. **IR** (ν/cm^{-1}): 3062 (w), 3022 (w), 2963 (s), 2932 (w), 2874 (m), 1812 (s), 1782 (m), 1653 (s), 1456 (m), 1292 (m), 1162 (m), 1020 (m), 944 (m), 882 (m). **HRMS** (ES^+) $[M–H]^+$ calcd for $C_{23}H_{26}NO_2^+$ 348.1964, found: 348.1971.

Synthesis of 4-(*E*-4-(furan-2-yl)but-3-en-2-yl)-4-isobutyl-2-phenyloxazol-5(4H)-one (20).

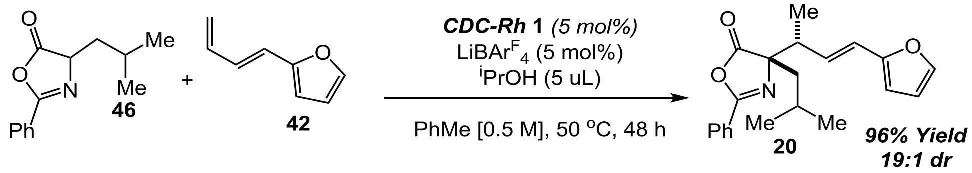

Following the general procedure for the Rh(I) catalyzed hydroalkylation of dienes with oxazalones, (CDC)-Rh(I)styrene BAr^F_4 **1** (8.1 mg, 0.005 mmol), $LiBAr^F_4$ (3.4 mg, 0.005 mmol), and 2-(buta-1,3-dien-1-yl)furan (12.0 mg, 0.100 mmol) were combined in the glove box, solvated with toluene (200 µL, 0.5 M) and allowed to stir at 22 °C for 10 minutes. To this solution, 4-isobutyl-2-phenyloxazol-5(4H)-one (32.6 mg, 0.150 mmol) was added. The reaction was sealed with a Teflon® septum cap and removed from the glove box. Outside the glove box, N_2 sparged isopropanol (5 µL) was added and the reaction allowed to stir at 50 °C for 48 h. The reaction was cooled to room temperature and 5 µL of hexamethyldisiloxane added as an internal standard. The reaction was diluted with $CDCl_3$ and analyzed by NMR spectroscopy as a 19:1 mixture of the anti:syn diastereomers. The NMR sample was recombined with the reaction and the solvents removed *in vacuo*. The resulting oil was purified by SiO_2 gel column chromatography (40:1 Hex/Et_2O) to afford **20** (32.4 mg, 0.096 mmol, 96% yield, 19:1 dr) as a colorless oil.

^{1}H NMR (600 MHz, $CDCl_3$) δ 8.06 – 8.02 (m, 2H), 7.61 – 7.56 (m, 1H), 7.50 (t, *J* = 7.7 Hz, 2H), 7.34 (d, *J* = 1.5 Hz, 1H), 6.36 (dd, *J* = 3.2, 1.8 Hz, 1H), 6.31 (d, *J* = 15.9 Hz, 1H), 6.22 (d, *J* = 3.2 Hz, 1H), 6.16 (dd, *J* = 15.9, 9.3 Hz, 1H), 2.71 (dq, *J* = 9.1, 6.8 Hz, 1H), 2.05 (dd, *J* = 14.2, 5.8 Hz, 1H), 1.76 (dd, *J* = 14.2, 6.9 Hz, 1H), 1.56 – 1.47 (m, 1H), 1.01 (d, *J* = 6.8 Hz, 3H), 0.84 (d, *J* = 6.7 Hz, 3H), 0.83 (d, *J* = 6.6 Hz, 3H). **^{13}C NMR** (151 MHz, $CDCl_3$) δ 181.2, 160.1, 152.6, 141.9, 132.8, 128.9, 128.5, 128.1, 126.0, 121.1, 111.4, 107.6, 76.1, 45.9, 44.8, 25.2, 24.2, 23.4, 15.6. **IR** (ν/cm^{-1}): 2961 (s), 2934 (w), 2908 (w), 2873 (m), 1812 (s), 1653 (s), 1456 (m), 1319 (w), 1291 (m), 1153 (m), 1022 (m), 961 (m), 882 (m). **HRMS** (ES^+) $[M–H]^+$ calcd for $C_{21}H_{24}NO_3^+$ 338.1756, found: 338.1763.

Synthesis of 4-(*E*-dodec-3-en-2-yl)-4-isobutyl-2-phenyloxazol-5(4H)-one (21).

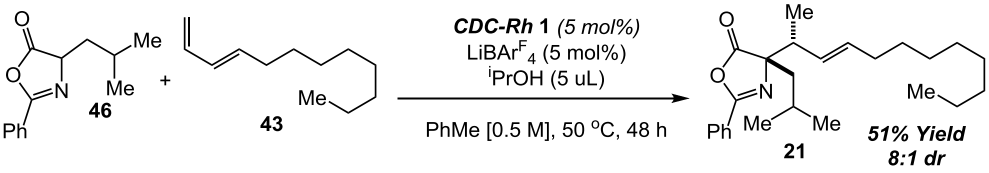

Following the general procedure for the Rh(I) catalyzed hydroalkylation of dienes with oxazalones, (CDC)-Rh(I)styrene $BAr^F{}_4$ **1** (8.1 mg, 0.005 mmol), $LiBAr^F{}_4$ (3.4 mg, 0.005 mmol), and 1,3-dodecadiene (16.6 mg, 0.100 mmol) were combined in the glove box, solvated with toluene (200 µL, 0.5 M) and allowed to stir at 22 °C for 10 minutes. To this solution, 4-isobutyl-2-phenyloxazol-5(4H)-one (32.6 mg, 0.150 mmol) was added. The reaction was sealed with a Teflon® septum cap and removed from the glove box. Outside the glove box, N_2 sparged isopropanol (5 µL) was added and the reaction allowed to stir at 50 °C for 48 h. The reaction was cooled to room temperature and 5 µL of hexamethyldisiloxane added as an internal standard. The reaction was diluted with $CDCl_3$ and analyzed by NMR spectroscopy as a 8:1 mixture of the anti:syn diastereomers. The NMR sample was recombined with the reaction and the solvents removed *in vacuo*. The resulting oil was purified by SiO_2 gel column chromatography (100% Hex to 40:1 Hex/Et_2O) to afford **21** (19.6 mg, 0.051 mmol, 51% yield, 8:1 dr) as a colorless oil.
^{1}H NMR (600 MHz, $CDCl_3$) δ 8.04 – 7.98 (m, 2H), 7.60 – 7.55 (m, 1H), 7.51 – 7.48 (m, 2H), 5.57 – 5.46 (m, 1H), 5.37 – 5.29 (m, 1H), 2.58 – 2.53 (m, 1H), 2.07 – 1.99 (m, 1H), 1.99 – 1.92 (m, 2H), 1.80 – 1.64 (m, 1H), 1.61 – 1.46 (m, 1H), 1.33 – 1.13 (m, 13H), 0.97 (d, J = 6.8 Hz, 2H), 0.89 – 0.83 (m, 6H), 0.82 (d, J = 6.6 Hz, 3H). **^{13}C NMR** (151 MHz, $CDCl_3$) δ 181.3, 159.7, 134.5, 132.6, 129.3, 128.9, 128.0, 126.2, 75.9, 45.7, 44.6, 32.7, 32.0, 29.6, 29.5, 29.4, 29.2, 25.2, 24.3, 23.2, 22.8, 15.4, 14.3. **IR** (ν/cm^{-1}): 2957 (m), 2925 (s), 2871 (w), 2854 (m), 1813 (s), 1654 (s), 1452 (m), 1320 (w), 1292 (m), 1160 (w), 1023 (m), 954 (m), 881 (m). **HRMS** (ES$^+$) $[M–H]^+$ calcd for $C_{25}H_{38}NO_2^+$ 384.2903, found: 384.2905.

Synthesis of 4-isobutyl-2-phenyl-4-(*E*-4-(o-tolyl)but-3-en-2-yl)oxazol-5(4H)-one (22).

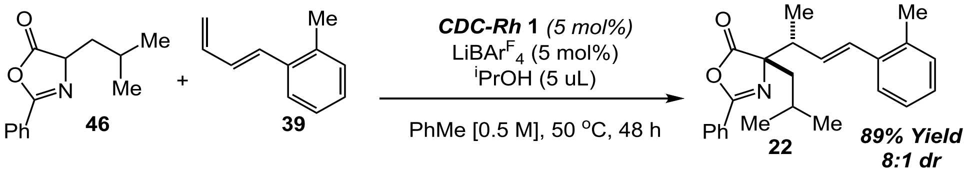

Following the general procedure for the Rh(I) catalyzed hydroalkylation of dienes with oxazalones, (CDC)-Rh(I)styrene $BAr^F{}_4$ **1** (8.1 mg, 0.005 mmol), $LiBAr^F{}_4$ (3.4 mg, 0.005 mmol), and *o*-methyl-phenylbutadiene (14.4 mg, 0.100 mmol) were combined in the glove box, solvated with toluene (200 µL, 0.5 M) and allowed to stir at 22 °C for 10 minutes. To this solution, 4-isobutyl-2-phenyloxazol-5(4H)-one (32.6 mg, 0.150 mmol) was added. The reaction was sealed with a Teflon® septum cap and removed from the glove box. Outside the glove box, N_2 sparged isopropanol (5 µL) was added and the reaction allowed to stir at 50 °C for 48 h. The reaction was cooled to room temperature and 5 µL of hexamethyldisiloxane added as an internal standard. The reaction was diluted with $CDCl_3$ and analyzed by NMR spectroscopy as a 10:1 mixture of the anti:syn diastereomers. The NMR sample was recombined with the reaction and the solvents

removed *in vacuo*. The resulting oil was purified by SiO_2 gel column chromatography (40:1 Hex/Et_2O) to afford **22** (32.2 mg, 0.089 mmol, 89% yield, 8:1 dr) as a colorless oil. The product was isolated with less than 5% of the inseperable 1,4-addition product.

^{1}H NMR (600 MHz, $CDCl_3$) δ 8.05 – 8.01 (m, 2H), 7.60 – 7.55 (m, 1H), 7.51 – 7.48 (m, 2H), 7.45 – 7.40 (m, 1H), 7.18 – 7.10 (m, 3H), 6.69 (d, *J* = 15.7 Hz, 1H), 6.05 (dd, *J* = 15.7, 9.4 Hz, 1H), 2.80 (dq, *J* = 13.6, 6.8 Hz, 1H), 2.30 (s, 3H), 2.07 (dd, *J* = 14.2, 5.7 Hz, 1H), 1.81 (dd, *J* = 14.2, 7.0 Hz, 1H), 1.58 – 1.51 (m, 1H), 1.07 (d, *J* = 6.8 Hz, 3H), 0.86 (d, *J* = 6.6 Hz, 3H), 0.84 (d, *J* = 6.6 Hz, 3H). **^{13}C NMR** (151 MHz, $CDCl_3$) δ 181.2, 160.0, 136.4, 135.4, 132.7, 131.2, 130.8, 130.3, 128.9, 128.1, 127.5, 126.2, 126.1, 126.0, 76.0, 46.3, 44.8, 25.2, 24.2, 23.4, 19.9, 15.6. **IR** (ν/cm^{-1}): 3062 (w), 3021 (w), 2960 (s), 2872 (m), 1812 (s), 1781 (m), 1653 (s), 1452 (m), 1292 (m), 1159 (m), 1023 (m), 956 (m), 882 (m). **HRMS** (ES$^+$) [M–H]$^+$ calcd for $C_{24}H_{28}NO_2^+$ 362.2120, found: 362.2123.

Synthesis of 4-(*E*-4-(furan-2-yl)but-3-en-2-yl)-4-phenethyl-2-phenyloxazol-5(4H)-one (23).

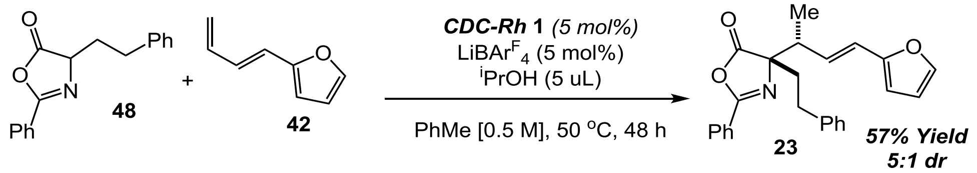

Following the general procedure for the Rh(I) catalyzed hydroalkylation of dienes with oxazalones, (CDC)-Rh(I)styrene BAr$^F_4$ **1** (8.1 mg, 0.005 mmol), LiBAr$^F_4$ (3.4 mg, 0.005 mmol), and 2-(buta-1,3-dien-1-yl)furan (12.0 mg, 0.100 mmol) were combined in the glove box, solvated with toluene (200 µL, 0.5 M) and allowed to stir at 22 °C for 10 minutes. To this solution, 4-phenethyl-2-phenyloxazol-5(4H)-one (39.8 mg, 0.150 mmol) was added. The reaction was sealed with a Teflon® septum cap and removed from the glove box. Outside the glove box, N_2 sparged isopropanol (5 µL) was added and the reaction allowed to stir at 50 °C for 48 h. The reaction was cooled to room temperature and 5 µL of hexamethyldisiloxane added as an internal standard. The reaction was diluted with $CDCl_3$ and analyzed by NMR spectroscopy as a 5:1 mixture of the anti:syn diastereomers. The NMR sample was recombined with the reaction and the solvents removed *in vacuo*. The resulting oil was purified by SiO_2 gel column chromatography (40:1 Hex/Et_2O) to afford **23** (22.0 mg, 0.057 mmol, 57% yield, >20:1 dr) as a colorless oil.

^{1}H NMR (600 MHz, $CDCl_3$) δ 8.10 – 8.04 (m, 2H), 7.60 (t, *J* = 7.4 Hz, 1H), 7.52 (t, *J* = 7.7 Hz, 2H), 7.33 (d, *J* = 1.3 Hz, 1H), 7.22 (t, *J* = 7.5 Hz, 2H), 7.15 (t, *J* = 7.4 Hz, 1H), 7.12 (d, *J* = 7.1 Hz, 2H), 6.35 (dd, *J* = 3.2, 1.8 Hz, 1H), 6.31 (d, *J* = 15.9 Hz, 1H), 6.24 – 6.17 (m, 2H), 2.80 (dq, *J* = 13.6, 6.8 Hz, 1H), 2.53 – 2.48 (m, 1H), 2.42 – 2.37 (m, 1H), 2.32 – 2.27 (m, 1H), 2.16 – 2.11 (m, 1H), 1.04 (d, *J* = 6.8 Hz, 3H). **^{13}C NMR** (151 MHz, $CDCl_3$) δ 180.5, 160.8, 152.5, 141.9, 140.6, 132.9, 129.0, 128.6, 128.6, 128.2, 128.2, 126.3, 125.8, 121.1, 111.4, 107.8, 76.6, 44.9, 38.1, 30.6, 16.0. **IR** (ν/cm^{-1}): 3063 (w), 3029 (m), 2966 (m), 2929 (m), 2873 (w), 1816 (s), 1653 (s), 1455 (m), 1320 (w), 1292 (m), 1059 (w), 997 (m), 877 (m). **HRMS** (ES$^+$) [M–H]$^+$ calcd for $C_{25}H_{24}NO_3^+$ 386.1756, found: 386.1761.

Synthesis of 4-(*E*-dodec-3-en-2-yl)-4-phenethyl-2-phenyloxazol-5(4H)-one (24).

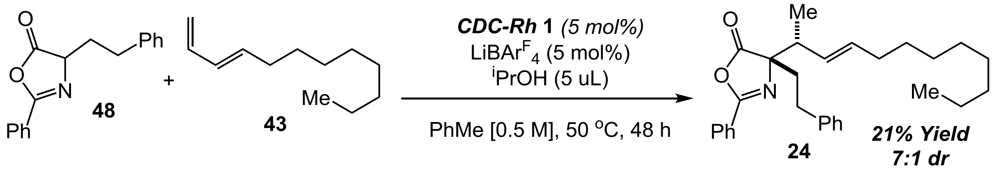

Following the general procedure for the Rh(I) catalyzed hydroalkylation of dienes with oxazalones, (CDC)-Rh(I)styrene BAr^F_4 **1** (8.1 mg, 0.005 mmol), $LiBAr^F_4$ (3.4 mg, 0.005 mmol), and 1,3-dodecadiene (16.6 mg, 0.100 mmol) were combined in the glove box, solvated with toluene (200 µL, 0.5 M) and allowed to stir at 22 °C for 10 minutes. To this solution, 4-phenethyl-2-phenyloxazol-5(4H)-one (39.8 mg, 0.150 mmol) was added. The reaction was sealed with a Teflon® septum cap and removed from the glove box. Outside the glove box, N_2 sparged isopropanol (5 µL) was added and the reaction allowed to stir at 50 °C for 48 h. The reaction was cooled to room temperature and 5 µL of hexamethyldisiloxane added as an internal standard. The reaction was diluted with $CDCl_3$ and analyzed by NMR spectroscopy as a 7:1 mixture of the anti:syn diastereomers. The NMR sample was recombined with the reaction and the solvents removed *in vacuo*. The resulting oil was purified by SiO_2 gel column chromatography (100% Hex to 40:1 Hex/Et_2O) to afford **24** (9.1 mg, 0.021 mmol, 21% yield, 8:1 dr) as a colorless oil.

^{1}H NMR (500 MHz, $CDCl_3$) δ 8.05 – 8.01 (m, 2H), 7.59 (t, *J* = 7.4 Hz, 1H), 7.51 (t, *J* = 7.6 Hz, 2H), 7.23 (d, *J* = 7.5 Hz, 2H), 7.16 (t, *J* = 7.4 Hz, 1H), 7.13 (d, *J* = 7.1 Hz, 2H), 5.59 – 5.50 (m, 1H), 5.40 (dd, *J* = 15.3, 9.1 Hz, 1H), 2.65 (dq, *J* = 13.8, 6.9 Hz, 1H), 2.56 – 2.50 (m, 1H), 2.43 – 2.37 (m, 1H), 2.31 – 2.22 (m, 1H), 2.16 – 2.09 (m, 1H), 1.98 (q, *J* = 6.8 Hz, 2H), 1.33 – 1.14 (m, 12H), 0.98 (d, *J* = 6.8 Hz, 3H), 0.87 (t, *J* = 7.1 Hz, 3H). **^{13}C NMR** (151 MHz, $CDCl_3$) δ 180.5, 160.4, 140.9, 134.4, 132.8, 129.6, 129.1, 128.9, 128.6, 128.1, 126.3, 126.0, 76.4, 44.5, 37.8, 32.7, 32.1, 30.5, 29.6, 29.5, 29.4, 29.3, 22.8, 15.8, 14.3. **IR** (ν/cm^{-1}): 3437 (br, m), 2957 (w), 2925 (s), 2854 (m), 1818 (s), 1653 (s), 1455 (m), 1321 (w), 1292 (m), 1059 (m), 995 (m), 877 (m). **HRMS** (ES$^+$) [M–H]$^+$ calcd for $C_{29}H_{38}NO_2^+$ 432.2903, found: 432.2906.

Synthesis of 4-phenethyl-2-phenyl-4-(*E*-4-(o-tolyl)but-3-en-2-yl)oxazol-5(4H)-one (25).

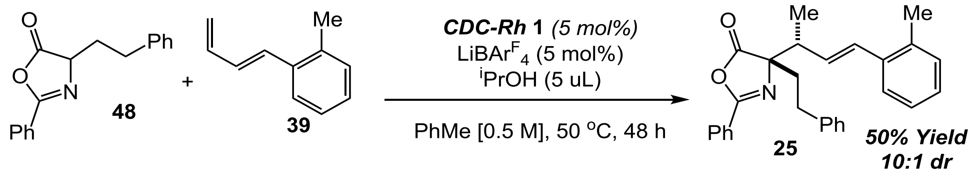

Following the general procedure for the Rh(I) catalyzed hydroalkylation of dienes with oxazalones, (CDC)-Rh(I)styrene BAr^F_4 **1** (8.1 mg, 0.005 mmol), $LiBAr^F_4$ (3.4 mg, 0.005 mmol), and *o*-methyl-phenylbutadiene (14.4 mg, 0.100 mmol) were combined in the glove box, solvated with toluene (200 µL, 0.5 M) and allowed to stir at 22 °C for 10 minutes. To this solution, 4-phenethyl-2-phenyloxazol-5(4H)-one (39.8 mg, 0.150 mmol) was added. The reaction was sealed with a Teflon® septum cap and removed from the glove box. Outside the glove box, N_2

sparged isopropanol (5 µL) was added and the reaction allowed to stir at 50 °C for 48 h. The reaction was cooled to room temperature and 5 µL of hexamethyldisiloxane added as an internal standard. The reaction was diluted with $CDCl_3$ and analyzed by NMR spectroscopy as a 10:1 mixture of the anti:syn diastereomers. The NMR sample was recombined with the reaction and the solvents removed *in vacuo*. The resulting oil was purified by SiO_2 gel column chromatography (40:1 Hex/Et_2O) to afford **25** (20.5 mg, 0.050 mmol, 50% yield, 10:1 dr) as a colorless oil.

^{1}H NMR (600 MHz, $CDCl_3$) δ 8.08 – 8.05 (m, 2H), 7.62 – 7.58 (m, 1H), 7.53 – 7.50 (m, 2H), 7.47 – 7.43 (m, 1H), 7.24 – 7.21 (m, 2H), 7.18 – 7.09 (m, 6H), 6.71 (d, *J* = 15.7 Hz, 1H), 6.11 (dd, *J* = 15.7, 9.4 Hz, 1H), 2.94 – 2.85 (m, 1H), 2.58 – 2.50 (m, 1H), 2.46 – 7.41 (m, 1H), 2.34 – 2.29 (m, 1H), 2.28 (s, 3H), 2.23 – 2.15 (m, 1H), 1.09 (d, *J* = 6.8 Hz, 3H). **^{13}C NMR** (151 MHz, $CDCl_3$) δ 180.4, 160.7, 140.6, 136.2, 135.4, 132.9, 130.8, 130.7, 130.4, 129.0, 128.6, 128.5, 128.2, 127.6, 126.3, 126.2, 125.9, 125.9, 76.4, 45.2, 38.0, 30.5, 19.9, 16.0. **IR** (ν/cm^{-1}): 3062 (w), 3027 (m), 2967 (m), 2929 (m), 2866 (w), 1816 (s), 1653 (s), 1496 (w), 1456 (m), 1292 (m), 1118 (m), 1058 (m), 996 (s), 877 (m). **HRMS** (ES^+) $[M–H]^+$ calcd for $C_{28}H_{28}NO_2^+$ 410.2121, found: 410.2124.

Synthesis of 4-(*E*-4-(furan-2-yl)but-3-en-2-yl)-4-phenethyl-2-phenyloxazol-5(4H)-one (26).

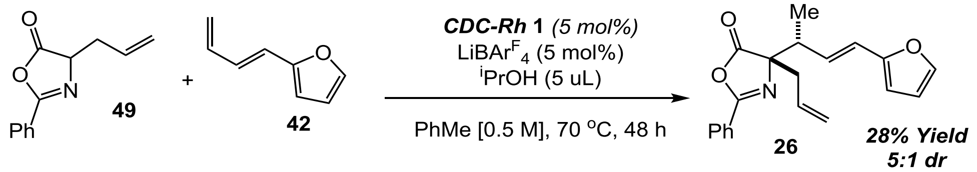

Following the general procedure for the Rh(I) catalyzed hydroalkylation of dienes with oxazalones, (CDC)-Rh(I)styrene $BAr^F{}_4$ **1** (8.1 mg, 0.005 mmol), $LiBAr^F{}_4$ (3.4 mg, 0.005 mmol), and 2-(buta-1,3-dien-1-yl)furan (12.0 mg, 0.100 mmol) were combined in the glove box, solvated with toluene (200 µL, 0.5 M) and allowed to stir at 22 °C for 10 minutes. To this solution, 4-allyl-2-phenyloxazol-5(4H)-one (30.2 mg, 0.150 mmol) was added. The reaction was sealed with a Teflon® septum cap and removed from the glove box. Outside the glove box, N_2 sparged isopropanol (5 µL) was added and the reaction allowed to stir at 70 °C for 48 h. The reaction was cooled to room temperature and 5 µL of hexamethyldisiloxane added as an internal standard. The reaction was diluted with $CDCl_3$ and analyzed by NMR spectroscopy as a 5:1 mixture of the anti:syn diastereomers. The NMR sample was recombined with the reaction and the solvents removed *in vacuo*. The resulting oil was purified by SiO_2 gel column chromatography (40:1 Hex/Et_2O) to afford **26** (9.0 mg, 0.028 mmol, 28% yield, 9:1 dr) as a colorless oil.

^{1}H NMR (600 MHz, $CDCl_3$) δ 8.04 – 8.03 (m, 2H), 7.62 – 7.57 (m, 1H), 7.51 – 7.48 (m, 2H), 7.35 (d, *J* = 1.5 Hz, 1H), 6.39 – 6.32 (m, 2H), 6.25 – 6.19 (m, 2H), 5.60 – 5.53 (m, 1H), 5.14 (d, *J* = 17.0, 1H), 5.06 (d, *J* = 10.2 Hz, 1H), 2.80 (dq, *J* = 9.2, 6.9 Hz, 1H), 2.75 (dd, *J* = 13.7, 6.4 Hz, 1H), 2.53 (dd, *J* = 13.8, 8.3 Hz, 1H), 1.02 (d, *J* = 6.8 Hz, 3H). **^{13}C NMR** (151 MHz, $CDCl_3$) δ 179.9, 160.5, 152.5, 142.0, 132.8, 131.0, 128.9, 128.2, 128.2, 125.9, 121.2, 120.7, 111.4, 107.8,

44.3, 40.5, 16.0. **IR** (ν/cm^{-1}): 2968 (m), 2927 (m), 1815 (s), 1654 (s), 1451 (m), 1322 (m), 1292 (m), 1152 (w), 1055 (m), 998 (m), 964 (m), 927 (m). **HRMS** (ES^+) $[M–H]^+$ calcd for $C_{20}H_{20}NO_3^+$ 322.1443, found: 322.1438.

Synthesis of 4-isobutyl-2-phenyl-4-(*E*-4-phenylbut-3-en-2-yl)oxazol-5(4H)-one (27).

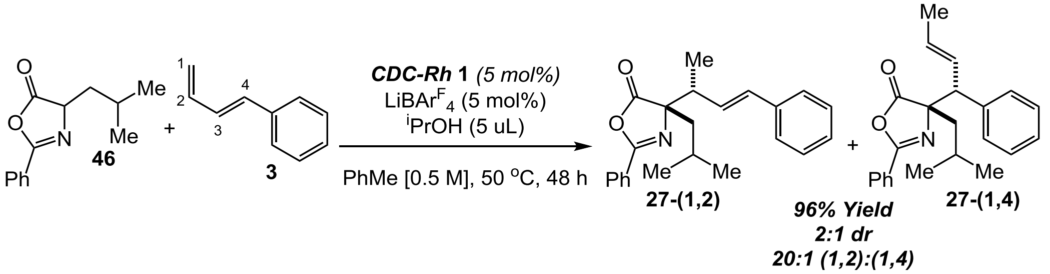

Following the general procedure for the Rh(I) catalyzed hydroalkylation of dienes with oxazalones, (CDC)-Rh(I)styrene BAr^F_4 **1** (8.1 mg, 0.005 mmol), $LiBAr^F_4$ (3.4 mg, 0.005 mmol), and phenylbutadiene (13.0 mg, 0.100 mmol) were combined in the glove box, solvated with toluene (200 µL, 0.5 M) and allowed to stir at 22 °C for 10 minutes. To this solution, 4-isobutyl-2-phenyloxazol-5(4H)-one (32.6 mg, 0.150 mmol) was added. The reaction was sealed with a Teflon® septum cap and removed from the glove box. Outside the glove box, N_2 sparged isopropanol (5 µL) was added and the reaction allowed to stir at 50 °C for 48 h. The reaction was cooled to room temperature and 5 µL of hexamethyldisiloxane added as an internal standard. The reaction was diluted with $CDCl_3$ and analyzed by NMR spectroscopy as a 2:1 mixture of the anti:syn diastereomers and a 20:1 mixture of the (1,2):(1,4) regioisomers. The NMR sample was recombined with the reaction and the solvents removed *in vacuo*. The resulting oil was purified by SiO_2 gel column chromatography (20:1 Hex/Et_2O) to afford **27** (33.4 mg, 0.0960 mmol, 96% yield, 2:1 dr, 19:1 (1,2):(1,4)) as a colorless oil.

***anti*-Diastereomer (major):** [^{1}**H NMR** (600 MHz, $CDCl_3$) δ 8.05 – 8.04 (m, 2H), 7.60 – 7.56 (m, 1H), 7.52 – 7.49 (m, 2H), 7.39 – 7.37 (m, 2H), 7.32 – 7.30 (m, 2H), 7.25 – 7.21 (m, 1H), 6.49 (d, *J* = 15.9 Hz, 1H), 6.21 (dd, *J* = 15.9, 9.3 Hz, 1H), 2.77 (dq, *J* = 9.1, 6.8 Hz, 1H), 2.43 (dd, *J* = 7.0, 4.3 Hz, 1H), 2.05 (dd, *J* = 14.2, 5.8 Hz, 1H), 1.78 (dd, *J* = 14.2, 6.9 Hz, 1H), 1.55 – 1.49 (m, 1H), 1.03 (d, *J* = 6.8 Hz, 3H), 0.83 (t, *J* = 7.0 Hz, 6H).] ***syn*-Diastereomer (minor):** [^{1}**H NMR** (600 MHz, $CDCl_3$) δ 8.05 – 8.03 (m, 2H), 7.60 – 7.57 (m, 1H), 7.51 – 7.49 (m, 2H), 7.39 – 7.36 (m, 2H), 7.28 – 7.23 (m, 2H), 7.23 – 7.18 (m, 1H), 6.35 (d, *J* = 15.9 Hz, 1H), 5.91 (dd, *J* = 15.9, 8.5 Hz, 1H), 3.18 – 3.11 (m, 1H), 2.43 (dd, *J* = 7.0, 4.3 Hz, 1H), 2.15 – 2.08 (m, 1H), 2.04 (dd, *J* = 14.2, 5.8 Hz, 1H), 1.77 (dd, *J* = 14.3, 6.8 Hz, 1H), 1.09 (d, *J* = 6.9 Hz, 3H), 0.90 (d, *J* = 6.7 Hz, 3H), 0.88 (d, *J* = 6.7 Hz, 3H).] **(1,4)-Regioisomer (minor):** [^{1}**H NMR** (600 MHz, $CDCl_3$) δ 7.99 – 7.96 (m, 2H), 7.56 – 7.54 (m, 1H), 7.50 – 7.46 (m, 2H), 7.38 – 7.32 (m, 2H), 7.30 – 7.26 (m, 2H), 7.23 – 7.18 (m, 1H), 5.69 – 5.58 (m, 2H), 3.61 (d, *J* = 8.9 Hz, 1H), 2.49 (dd, *J* = 7.0, 2.0 Hz, 1H), 2.24 – 2.16 (m, 1H), 1.68 (dd, *J* = 14.3, 5.9 Hz, 1H), 1.60 (d, *J* = 5.1 Hz, 3H), 0.78 (d, *J* = 6.6 Hz, 3H), 0.76 (d, *J* = 6.6 Hz, 3H).] ^{13}**C NMR** (151 MHz, $CDCl_3$) δ 181.1, 165.5, 162.2, 159.9, 138.0, 137.0, 136.8, 133.8, 132.6, 129.7, 129.1, 128.8, 128.7, 128.5, 128.5, 128.3, 128.0, 127.6, 127.5, 127.3, 126.5, 126.4, 126.3, 125.9, 109.2, 76.0, 47.0, 46.0, 44.7, 36.5,

26.3, 25.1, 24.1, 23.3, 22.6, 22.4, 15.5, 15.2. **IR** (ν/cm^{-1}): 3028 (w), 2960 (s), 2934 (w), 2872 (m), 1812 (s), 1781 (m), 1653 (s), 1495 (m), 1450 (m), 1320 (w), 1292 (m), 1158 (m), 1023 (w), 960 (s), 882 (m). **HRMS** (ES$^+$) [M–H]$^+$ calcd for $C_{23}H_{26}NO_2^+$ 348.1964, found: 348.1958.

Synthesis of 2-(4-chlorophenyl)-4-methyl-4-(*E*-4-phenylbut-3-en-2-yl)oxazol-5(4H)-one (28).

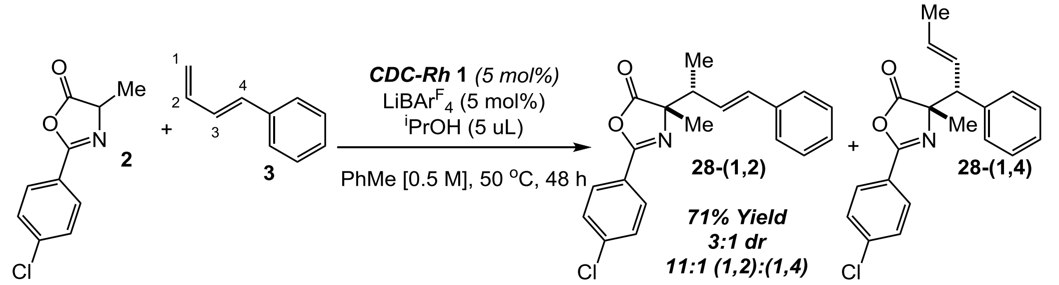

Following the general procedure for the Rh(I) catalyzed hydroalkylation of dienes with oxazalones, (CDC)-Rh(I)styrene BAr^F_4 **1** (8.1 mg, 0.005 mmol), $LiBAr^F_4$ (3.4 mg, 0.005 mmol), and phenylbutadiene (13.0 mg, 0.100 mmol) were combined in the glove box, solvated with toluene (200 µL, 0.5 M) and allowed to stir at 22 °C for 10 minutes. To this solution, 2-(4-chlorophenyl)-4-methyloxazol-5(4H)-one (31.4 mg, 0.150 mmol) was added. The reaction was sealed with a Teflon® septum cap and removed from the glove box. Outside the glove box, N_2 sparged isopropanol (5 µL) was added and the reaction allowed to stir at 50 °C for 48 h. The reaction was cooled to room temperature and 5 µL of hexamethyldisiloxane added as an internal standard. The reaction was diluted with $CDCl_3$ and analyzed by NMR spectroscopy as a 3:1 mixture of the anti:syn diastereomers and a 11:1 mixture of the (1,2):(1,4) regioisomers. The NMR sample was recombined with the reaction and the solvents removed *in vacuo*. The resulting oil was purified by SiO_2 gel column chromatography (20:1 Hex/Et_2O to 10:1 Hex/Et_2O) to afford **28** (24.1 mg, 0.0710 mmol, 71% yield, 3:1 dr, 11:1 (1,2):(1,4)) as a colorless oil.
***anti*-Diastereomer (major):** [^{1}**H NMR** (400 MHz, $CDCl_3$) δ 7.99 – 7.95 (m, 2H), 7.51 – 7.44 (m, 2H), 7.42 – 7.37 (m, 2H), 7.33 – 7.30 (m, 2H), 7.29 – 7.22 (m, 1H), 6.53 (d, *J* = 15.9 Hz, 1H), 6.22 (dd, *J* = 15.9, 9.3 Hz, 1H), 2.80 (dq, *J* = 13.7, 6.9 Hz, 1H), 1.50 (s, 3H), 1.04 (d, *J* = 6.8 Hz, 3H).] ***syn*-Diastereomer (minor):** [^{1}**H NMR** (400 MHz, $CDCl_3$) δ 7.99 – 7.95 (m, 2H), 7.51 – 7.44 (m, 2H), 7.42 – 7.37 (m, 2H), 7.34 – 7.30 (m, 2H), 7.26 – 7.20 (m, 1H), 6.33 (d, *J* = 16.0 Hz, 1H), 5.88 (dd, *J* = 16.0, 8.4 Hz, 1H), 3.12 – 2.98 (m, 1H), 1.59 (s, 3H), 1.07 (d, *J* = 6.9 Hz, 3H).] **(1,4)-Regioisomer (minor): [^{1}H NMR** (400 MHz, $CDCl_3$) δ 7.96 – 7.90 (m, 2H), 7.84 – 7.77 (m, 2H), 7.44 – 7.41 (m, 2H), 7.34 - 7.27 (m, 1H), 7.14 - 7.12 (m, 2H), 5.78 – 5.57 (m, 2H), 3.66 (d, *J* = 9.8 Hz, 1H), 2.24 (s, 3H), 1.76 (dd, *J* = 6.4, 1.5 Hz, 3H).] ^{13}**C NMR** (151 MHz, $CDCl_3$) δ 181.0, 165.5, 163.2, 161.6, 160.3, 160.0, 137.6, 133.2, 133.1, 132.9, 132.8, 131.7, 129.1, 129.1, 128.9, 128.9, 128.5, 128.1, 127.9 (dd, *J* = 20.7, 8.0 Hz), 127.1, 127.1, 126.6, 126.0, 115.6 (dd, *J* = 21.6, 10.7 Hz), 109.2, 72.5, 47.0, 45.1, 22.8, 15.9, 15.1, 14.0. **IR** (ν/cm^{-1}): 3028 (m), 2976 (m), 2933 (m), 2873 (w), 1823 (m), 1783 (s), 1653 (m), 1490 (m), 1403 (w), 1311 (m), 1171 (m), 1092 (m), 1000 (m), 967 (m), 840 (m). **HRMS** (ES$^+$) [M–H]$^+$ calcd for $C_{20}H_{19}ClNO_2^+$ 340.1104, found: 340.1099.

■ **General procedure for hydrolysis of oxazolone products in Table 4 (29-31):**
An 8 mL reaction vial with a stir bar was charged with the oxazolone and potassium carbonate with no effort to exclude oxygen or water. The reaction was solvated with wet methanol and the headspace purged with N_2 for 5 minutes. The reaction was sealed and allowed to stir at room temperature for a minimum of 2 hours before being concentrated by rotary evaporation to remove the solvent. The resulting powder was purified by SiO_2 gel chromatography to yield the hydrolyzed product.

■ **Procedure and characterization for hydrolysis of oxazolone products in Table 4:**
Synthesis of methyl (*E*)-2-benzamido-2,3-dimethyl-5-phenylpent-4-enoate (29).

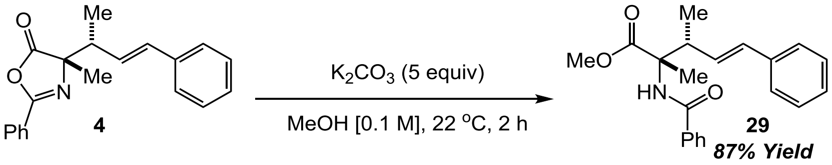

Following the general procedure for hydrolysis, 4-methyl-2-phenyl-4-((*E*)-4-phenylbut-3-en-2-yl)oxazol-5(4H)-one (12.1 mg, 0.0396 mmol, 19:1 dr) and K_2CO_3 (27.4 mg, 0.198 mmol, 5 equiv) were solvated in methanol (400 µL) and allowed to stir at 22 °C for 2 h. The solution was concentrated to an off white solid which was purified by SiO_2 gel column chromatography (10:1 Hex/Et_2O) to afford **29** (11.1 mg, 0.0344 mmol, 87% yield, 20:1 dr) as a colorless oil.
^{1}H NMR (600 MHz, $CDCl_3$) δ 7.73 – 7.70 (m, 2H), 7.48 – 7.45 (m, 1H), 7.39 – 7.37 (m, 2H), 7.37 – 7.33 (m, 2H), 7.31 (t, *J* = 7.6 Hz, 2H), 7.24 (t, *J* = 7.3 Hz, 1H), 6.84 (s, 1H), 6.52 (d, *J* = 15.8 Hz, 1H), 6.17 (dd, *J* = 15.8, 9.3 Hz, 1H), 3.79 (s, 3H), 3.00 (dq, *J* = 14.0, 7.0 Hz, 1H), 1.80 (s, 3H), 1.22 (d, *J* = 7.0 Hz, 3H). **^{13}C NMR** (151 MHz, $CDCl_3$) δ 173.5, 166.9, 136.9, 134.8, 132.6, 131.7, 130.3, 128.7, 128.7, 127.8, 127.0, 126.5, 62.8, 52.6, 45.3, 20.8, 15.9. **IR** (ν/cm^{-1}): 3410 (br, m), 3334 (br, m), 3027 (m), 2975 (m), 2949 (m), 1739 (s), 1653 (s), 1521 (s), 1488 (m), 1373 (m), 1263 (m), 1127 (m), 970 (m). **HRMS** (ES^+) $[M–H]^+$ calcd for $C_{21}H_{24}NO_3^+$ 338.1756, found: 338.1750.

Synthesis of methyl *(E)*-2-benzamido-5-(furan-2-yl)-2-isobutyl-3-methylpent-4-enoate (30).

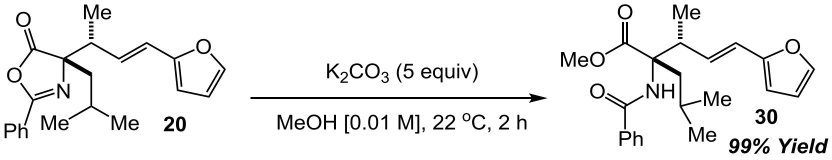

Following the general procedure for hydrolysis, 4-*((E)*-4-(furan-2-yl)but-3-en-2-yl)-4-isobutyl-2-phenyloxazol-5(4H)-one (8.5 mg, 0.025 mmol, 9:1 dr) and K_2CO_3 (17.4 mg, 0.126 mmol, 5 equiv) were solvated in methanol (2 mL) and allowed to stir at 22 °C for 2 h. The solution was concentrated to an off white solid which was purified by SiO_2 gel column chromatography (10:1 Hex/Et_2O) to afford **30** (8.3 mg, 0.0248 mmol, 89% yield, >20:1 dr) as a colorless oil.
^{1}H NMR (600 MHz, $CDCl_3$) δ 7.78 – 7.74 (m, 2H), 7.49 (t, *J* = 7.4 Hz, 1H), 7.42 (t, *J* = 7.6 Hz, 2H), 7.37 (s, 1H), 7.28 (d, *J* = 1.2 Hz, 1H), 6.30 (dd, *J* = 3.2, 1.8 Hz, 1H), 6.18 (d, *J* = 15.7 Hz, 1H), 6.07 (d, *J* = 3.2 Hz, 1H), 5.94 (dd, *J* = 15.7, 9.1 Hz, 1H), 3.84 (s, 3H), 3.57 – 3.49 (m, 1H), 2.73 (dd, *J* = 14.1, 4.3 Hz, 1H), 1.95 (dd, *J* = 14.0, 9.0 Hz, 1H), 1.68 – 1.55 (m, 1H), 1.21 (d, *J* =

7.0 Hz, 3H), 0.93 (d, J = 6.7 Hz, 3H), 0.77 (d, J = 6.6 Hz, 3H). **^{13}C NMR** (151 MHz, $CDCl_3$) δ 174.5, 166.6, 152.7, 141.8, 135.6, 131.5, 129.3, 128.7, 127.0, 120.4, 111.2, 107.4, 67.2, 52.7, 43.1, 41.0, 25.2, 24.3, 22.0, 15.8. **IR** (ν/cm^{-1}): 3413 (w), 2962 (s), 2923 (m), 2866 (w), 1731 (m), 1669 (s), 1508 (m), 1488 (w), 1260 (s), 1095 (br, s), 1021 (br, s), 799 (s). **HRMS** (ES^+) $[M–H]^+$ calcd for $C_{22}H_{28}NO_4^+$ 370.2018, found: 370.2013.

Synthesis of methyl methyl (*E*)-2-benzamido-3-methyl-2-propyltridec-4-enoate (A).

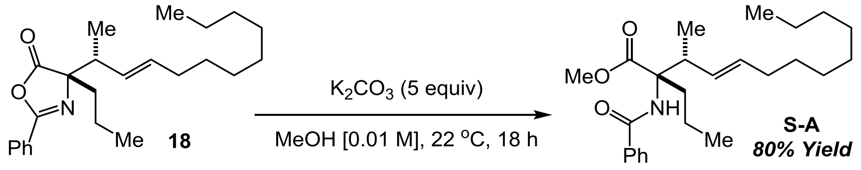

Following the general procedure for hydrolysis, 4-((*E*)-dodec-3-en-2-yl)-2-phenyl-4-propyloxazol-5(4H)-one (17.1 mg, 0.0463 mmol, 9:1 dr) and K_2CO_3 (320 mg, 2.31 mmol, 5 equiv) were solvated in methanol (4 mL) and allowed to stir at 22 °C for 18 h. The solution was concentrated to an off white solid which was purified by SiO_2 gel column chromatography (5:1 Hex/Et_2O) to afford **S-A** (14.9 mg, 0.0371 mmol, 80% yield, 9:1 dr) as a colorless oil.
^{1}H NMR (600 MHz, $CDCl_3$) δ 7.77 – 7.75 (m, 2H), 7.50 – 7.47 (m, 1H), 7.44 – 7.41 (m, 2H), 7.09 (s, 1H), 5.51 – 5.43 (m, 1H), 5.26 (dd, J = 15.2, 9.1 Hz, 1H), 3.79 (s, 3H), 3.12 (dq, J = 14.2, 7.1 Hz, 1H), 2.54 (ddd, J = 13.8, 12.1, 4.6 Hz, 1H), 2.10 (ddd, J = 13.8, 12.1, 4.5 Hz, 1H), 1.96 – 1.92 (m, 2H), 1.38 – 1.14 (m, 14H), 1.10 (d, J = 7.0 Hz, 3H), 0.90 (t, J = 7.3 Hz, 3H), 0.87 (t, J = 7.1 Hz, 3H). **^{13}C NMR** (151 MHz, $CDCl_3$) δ 174.02, 166.20, 135.41, 133.46, 131.48, 130.64, 128.66, 126.95, 67.31, 52.61, 42.29, 34.06, 32.68, 32.03, 29.68, 29.59, 29.39, 29.24, 22.81, 17.96, 16.05, 14.27, 14.25. **IR** (ν/cm^{-1}): 3415 (m), 2956 (m), 2925 (s), 2854 (m), 1730 (s), 1669 (s), 1515 (m), 1486 (w), 1232 (m), 971 (w). **HRMS** (ES^+) $[M–H]^+$ calcd for $C_{25}H_{39}NO_3^+$ 402.3008, found: 402.3006.

Synthesis of methyl *(E)*-2-benzamido-2-isobutyl-3-methyltridec-4-enoate (31).

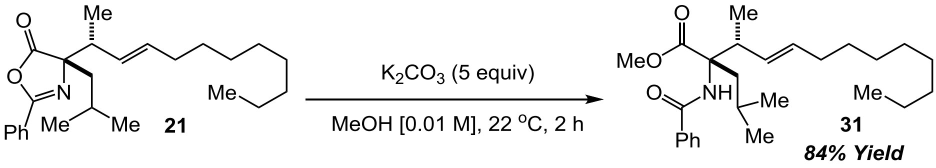

Following the general procedure for hydrolysis, 4-(*(E)*-dodec-3-en-2-yl)-4-isobutyl-2-phenyloxazol-5(4H)-one (9.2 mg, 0.024 mmol, 11:1 dr) and K_2CO_3 (16.6 mg, 0.120 mmol, 5 equiv) were solvated in methanol (2 mL) and allowed to stir at 22 °C for 2 h. The solution was concentrated to an off white solid which was purified by SiO_2 gel column chromatography (20:1 Hex/Et_2O) to afford **31** (8.4 mg, 0.020 mmol, 84% yield, 10:1 dr) as a colorless oil.
^{1}H NMR (600 MHz, $CDCl_3$) δ 7.78 – 7.75 (m, 2H), 7.49 (t, J = 7.4 Hz, 1H), 7.43 (t, J = 7.5 Hz, 2H), 7.29 (s, 1H), 5.45 – 5.39 (m, 1H), 5.17 (dd, J = 15.2, 9.0 Hz, 1H), 3.80 (s, 3H), 3.31 – 3.25 (m, 1H), 2.63 (dd, J = 14.1, 4.2 Hz, 1H), 1.96 (dd, J = 14.1, 9.0 Hz, 1H), 1.92 – 1.89 (m, 2H), 1.64-1.59 (m, 1H), 1.28 – 1.13 (br m, 12H), 1.10 (d, J = 7.0 Hz, 3H), 0.92 (d, J = 6.7 Hz, 3H),

0.87 (t, J = 7.2 Hz, 3H), 0.76 (d, J = 6.6 Hz, 3H). **^{13}C NMR** (151 MHz, $CDCl_3$) δ 174.7, 166.3, 135.7, 133.4, 131.4, 130.4, 128.7, 126.9, 66.9, 52.5, 42.7, 40.8, 32.7, 32.0, 29.6, 29.6, 29.4, 29.2, 25.1, 24.4, 22.8, 22.1, 15.9, 14.3. **IR** (ν/cm^{-1}): 3416 (br, s), 2955 (m), 2925 (s), 2854 (m), 1726 (m), 1669 (s), 1514 (m), 1485 (m), 1366 (m), 1235 (m), 970 (w). **HRMS** (ES$^+$) [M–H]$^+$ calcd for $C_{26}H_{42}NO_3^+$ 416.3165, found: 416.3160.

■ **Procedure and characterization for the functionalization of oxazolone products in Table 5 (32-33):**

Synthesis of (*E*)-2-benzamido-3-methyl-2-phenethyl-5-(o-tolyl)pent-4-enoic acid (32).

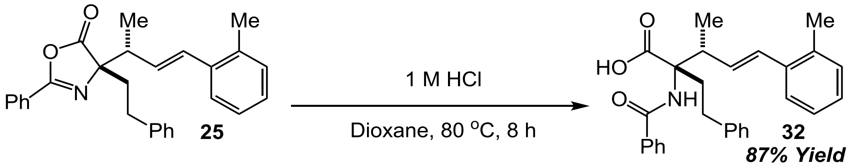

To an 8 mL vial was added 4-phenethyl-2-phenyl-4-(*E*-4-(o-tolyl)but-3-en-2-yl)oxazol-5(4H)-one (6.4 mg, 0.016 mmol, 10:1 dr), dioxane (1 mL) and 1M HCl (1 mL). The reaction was sealed with a septum cap and the headspace flushed with N_2 before being heated to 80 °C. The reaction was allowed to stir at 80°C for 8 h before being cooled to room temperature and extracted three times with EtOAc (1 mL). The organic layers were dried with Na_2SO_4 and filtered before being concentrated. The resulting oil was dried by rotoray evaporation with additional chloroform to remove residual dioxane to yield **32** as a clear film (5.9 mg, 0.014 mmol, 87% yield, >20:1 dr). The product required no further purification.

^{1}H NMR (600 MHz, $CDCl_3$) δ 7.77 – 7.75 (m, 2H), 7.51 (t, J = 7.4 Hz, 1H), 7.42 (t, J = 7.7 Hz, 2H), 7.39 – 7.38 (m, 1H), 7.24 (d, J = 7.4 Hz, 2H), 7.20 (d, J = 7.0 Hz, 2H), 7.18 – 7.14 (m, 2H), 7.13 – 7.09 (m, 2H), 7.08 – 7.06 (m, 1H), 6.66 (d, J = 15.5 Hz, 1H), 6.00 (dd, J = 15.5, 9.3 Hz, 1H), 3.51 (dq, J = 14.0, 7.0 Hz, 1H), 3.06 – 2.97 (m, 1H), 2.75 – 2.67 (m, 1H), 2.56 – 2.47 (m, 2H), 2.16 (s, 3H), 1.29 (d, J = 7.0 Hz, 3H). **^{13}C NMR** (151 MHz, $CDCl_3$) δ 176.1, 167.3, 141.3, 136.3, 135.4, 134.7, 132.0, 131.6, 130.6, 130.3, 128.9, 128.8, 128.6, 127.6, 127.0, 126.2, 126.2, 126.1, 67.8, 43.2, 34.3, 31.2, 19.8, 15.9. **IR** (ν/cm^{-1}): 3384 (br, m), 3220 (br, m), 3062 (w), 3027 (m), 2972 (m), 2930 (m), 2561 (br, m), 1715 (s), 1625 (s), 1523 (s), 1488 (m), 1455 (w), 1231 (m), 1192 (m), 1122 (w), 967 (m), 909 (m). **HRMS** (ES$^+$) [M–H]$^+$ calcd for $C_{25}H_{40}NO_4^+$ 428.2226, found: 428.2237.

Synthesis of methyl 2-benzamido-2-(1-(3-octyloxiran-2-yl)ethyl)pentanoate (33).

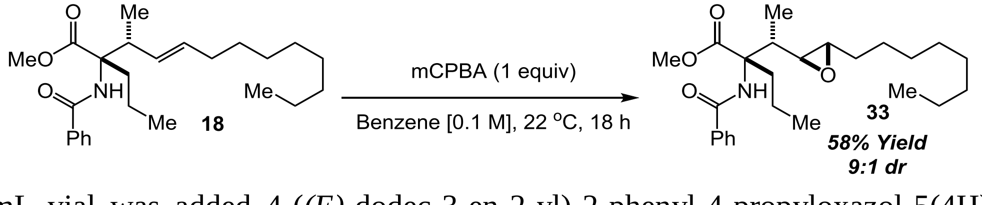

To an 8 mL vial was added 4-(*(E)*-dodec-3-en-2-yl)-2-phenyl-4-propyloxazol-5(4H)-one (8.9 mg, 0.022 mmol, 9:1 dr) and *meta*-chloroperoxybenzoic acid 50-55% by weight (7.6 mg, 0.22 mmol, 1 equiv). The headspace was flushed with N_2 and the reaction solvated with dry benzene

(500 µL), the reaction sealed and allowed to stir at 22 °C for 18 h. The solution was concentrated to an oily solid which was purified by SiO_2 gel column chromatography (8:1 Hex/EtOAc to 4:1 Hex/EtOAc) to afford **33** (5.4 mg, 0.013 mmol, 58% yield, 9:1 dr) as a colorless oil.

^{1}H NMR (600 MHz, $CDCl_3$) δ 7.82 – 7.79 (m, 2H), 7.52 (t, *J* = 7.4 Hz, 1H), 7.46 (t, *J* = 7.6 Hz, 2H), 7.42 (s, 1H), 3.85 (s, 3H), 2.86 (ddd, *J* = 13.5, 12.0, 4.6 Hz, 1H), 2.82 – 2.80 (m, 1H), 2.72 (dd, *J* = 7.8, 2.2 Hz, 1H), 2.39 (dt, *J* = 14.5, 7.1 Hz, 1H), 1.93 – 1.86 (m, 1H), 1.55 – 1.50 (m, 1H), 1.47 – 1.38 (m, 1H), 1.38 – 1.15 (m, 14H), 1.07 (d, *J* = 7.0 Hz, 3H), 1.00 – 0.94 (m, 1H), 0.90 (t, *J* = 7.2 Hz, 3H), 0.87 (t, *J* = 7.1 Hz, 3H). **^{13}C NMR** (151 MHz, $CDCl_3$) δ 174.6, 166.2, 135.1, 131.8, 128.8, 127.0, 67.2, 60.0, 59.8, 53.3, 42.8, 34.3, 32.2, 32.0, 29.6, 29.6, 29.3, 26.0, 22.8, 18.0, 14.3, 14.1, 12.6. **IR** (ν/cm^{-1}): 3410 (br, m), 2960 (w), 2928 (s), 2855 (m), 1732 (s), 1671 (s), 1518 (s), 1487 (m), 1271 (w), 1234 (m). **HRMS** (ES$^+$) $[M–H]^+$ calcd for $C_{25}H_{40}NO_4^+$ 418.2957, found: 418.2951.

■ General procedure for exploring the effect of the alcohol additive on hydroalkylation Table 6:

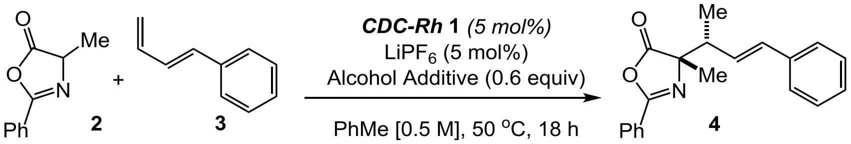

Following the general procedure for the Rh(I) catalyzed hydroalkylation of dienes with oxazalones, (CDC)-Rh(I)styrene BAr^F_4 **1** (8.1 mg, 0.005 mmol), $LiPF_6$ (0.8 mg, 0.005 mmol), and phenylbutadiene (13.0 mg, 0.100 mmol) were combined in the glove box, solvated with toluene (200 µL, 0.5 M) and allowed to stir at 22 °C for 10 minutes. To this solution, 4-methyl-2-phenyloxazol-5(4H)-one (26.3 mg, 0.150 mmol) was added. The reaction was sealed with a Teflon® septum cap and removed from the glove box. To this reaction the appropriate alcohol additive was added either: 1) Inside the glove [eg: menthol (9.4 mg, 0.06 mmol), (R)-BINOL (17.0 mg, 0.060 mmol), TADDOL-P(O)OH (31.7 mg, 0.060 mmol), (S,S)-hydrobenzoin (12.9 mg, 0.060 mmol) or (S,S)-diphenylethylenediamine (12.7 mg, 0.060 mmol)], or 2) Outside the glove box via syringe after sparging the alcohol with N_2 [eg: methanol (2.4 µL, 0.060 mmol), isopropanol (4.6 µL, 0.060 mmol), *tert*-butanol (5.7 µL, 0.060 mmol)]. The reaction were allowed to stir at 50 °C for 18 h. The reaction was cooled to room temperature and 5 µL of hexamethyldisiloxane added as an internal standard. The reaction was diluted with $CDCl_3$ and both the conversion and diastereoselectivity analyzed by NMR spectroscopy. Reactions with a chiral additive were purified by by SiO_2 gel column chromatography (20:1 Hex/Et2O) before being assayed on an Agilent 1220 LC System with a Daicel ChiralPak IA column (99:1 Hexanes/Isopropanol, 1 mL/min, 210 nm).

Table 6. Exploring the Influence of the Alcohol on Reaction Efficiency and Selectivity.

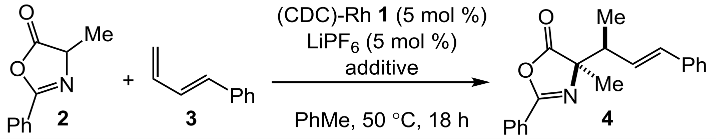

entry	additive; mol %	NMR Yield (%)[a]; dr[b]	% ee
1	MeOH; 60	68; 7:1	-
2	iPrOH; 60	84; 19:1	-
3	tBuOH; 60	87; 12:1	-
4	Menthol; 60	62; >20:1	0
5	*(R)*-BINOL; 60	56; 15:1	0
6	*(S,S)*-hydrobenzoin; 60	53; 10:1	0
7	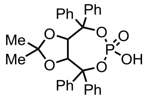 ; 60	13; 2:1	0
8	(*S,S)*-diphenylethylenediamine; 10	27; 18:1	0

[a]Values determined by analysis of 500 or 600 MHz ^{1}H NMR spectra of unpurified mixtures with trimethylsilyl ether as an internal standard. [b]NMR Yield reported for conversion to the *cis*- and *trans*-**4** products.

Table 1. Survey of Conditions for (CDC)-Rh-Catalyzed Diastereo- and Siteselective Hydroalkylation of 1,3 Diene **3.**[a]

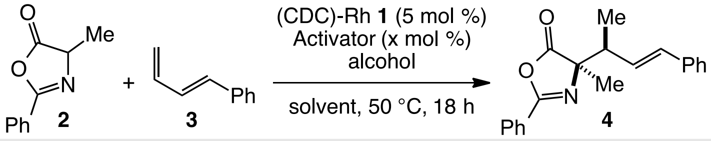

entry	activator; mol %	solvent	alcohol[d]	yield (%)[c]; dr[b]
1	AgCl; 5	PhMe	-	0; -
2	$LiBF_4$; 5	PhMe	-	8; 4:1
3	$LiPF_6$; 5	PhMe	-	17; 10:1
4	$LiPF_6$; 5	PhCl	-	21; 6:1
5	$LiPF_6$; 5	THF	-	21; 4:1
6	$LiPF_6$; 5	DCM	-	20; 3:1
7	$LiPF_6$; 5	PhMe	MeOH	26; 3:1
8	***$LiPF_6$; 5***	***PhMe***	***^{i}PrOH***	***85; 19:1***
9	$LiPF_6$; 5	PhMe	^{t}BuOH	29; 5:1
10[e]	$LiPF_6$; 5	PhMe	^{i}PrOH	0; -

[a]All reactions performed under N_2 atm. [b]Values determined by analysis of 400 or 600 MHz ^{1}H NMR spectra of unpurified mixtures with trimethylsilyl ether as an internal standard. [c]Yields of purified products are an average of two runs. [d]A solvent ratio of 40:1 PhMe:alcohol used. [e]Reaction run with $[Rh(cod)Cl]_2$ as catalyst with $NaBAr^F{}_4$ additive.

References:

(1) Lishchynskyi, A.; Muñiz, K. *Chem. – Eur. J.* **2012**, *18* (8), 2212.
(2) Preuß, T.; Saak, W.; Doye, S. *Chem. – Eur. J.* **2013**, *19* (12), 3833.

(3) Macreadie, I. G.; Avery, T. D.; Robinson, T. V.; Macreadie, P.; Barraclough, M.; Taylor, D. K.; Tiekink, E. R. T. *Tetrahedron* **2008**, *64* (7), 1225.
(4) Galvani, G.; Lett, R.; Kouklovsky, C. *Chem. – Eur. J.* **2013**, *19* (46), 15604.
(5) Townsend, E. M.; Schrock, R. R.; Hoveyda, A. H. *J. Am. Chem. Soc.* **2012**, *134* (28), 11334.
(6) Weber, M.; Jautze, S.; Frey, W.; Peters, R. *J. Am. Chem. Soc.* **2010**, *132* (35), 12222.
(7) Esteban, F.; Alfaro, R.; Yuste, F.; Parra, A.; Ruano, J. L. G.; Alemán, J. *Eur. J. Org. Chem.* **2014**, *2014* (7), 1395.
(8) Metrano, A. J.; Miller, S. J. *J. Org. Chem.* **2014**, *79* (4), 1542.
(9) Melhado, A. D.; Luparia, M.; Toste, F. D. *J. Am. Chem. Soc.* **2007**, *129* (42), 12638.
(10) Yakelis, N. A.; Bergman, R. G. *Organometallics* **2005**, *24* (14), 3579.
(11) Goldfogel, M. J.; Roberts, C. C.; Meek, S. J. *J. Am. Chem. Soc.* **2014**, *136* (17), 6227.
(12) Roberts, C. C.; Matías, D. M.; Goldfogel, M. J.; Meek, S. J. *J. Am. Chem. Soc.* **2015**, *137* (20), 6488.
(13) Voituriez, A.; Charette, A. B. *Adv. Synth. Catal.* **2006**, *348* (16-17), 2363.

^{1}H NMR (600 MHz, $CDCl_3$)

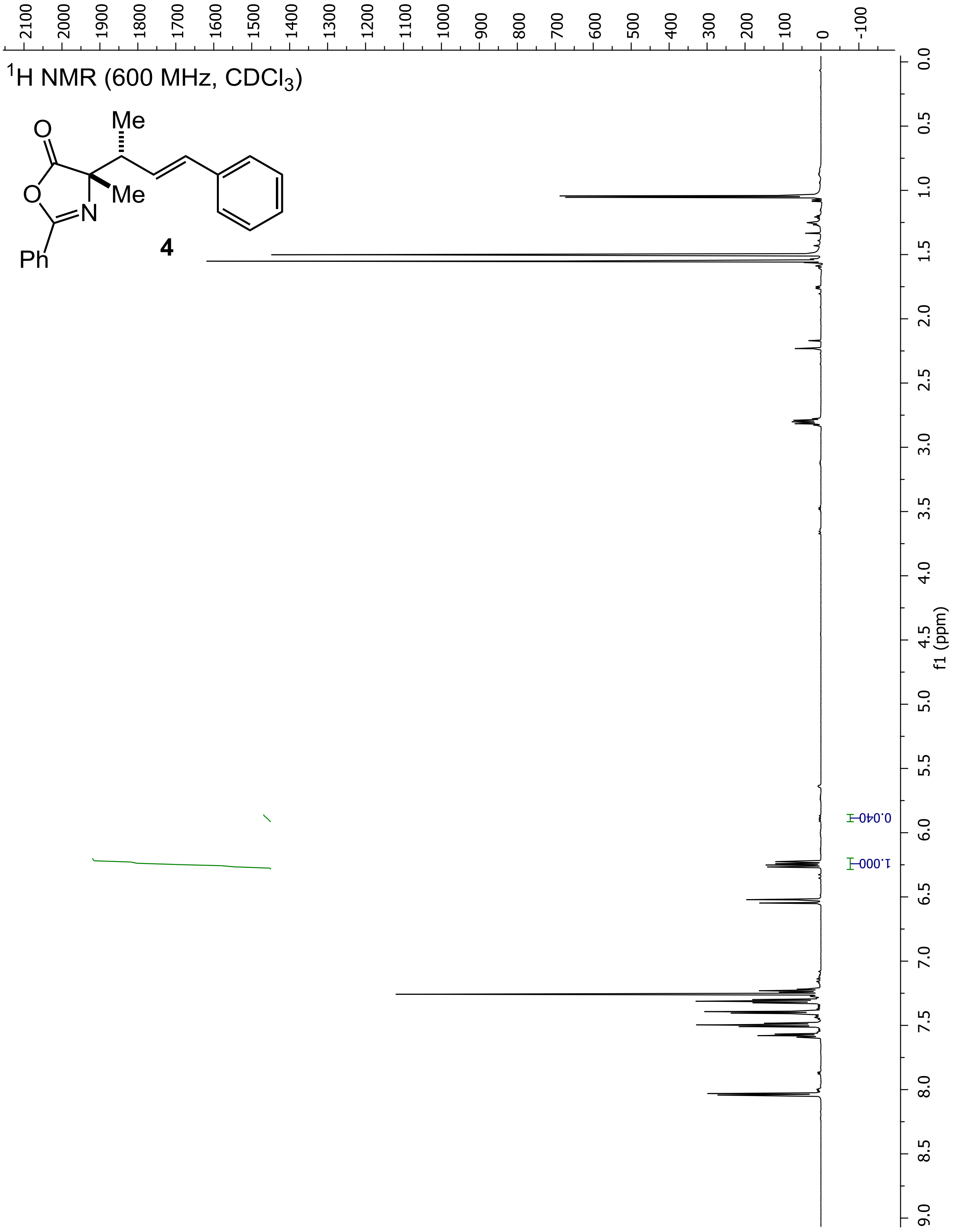

4

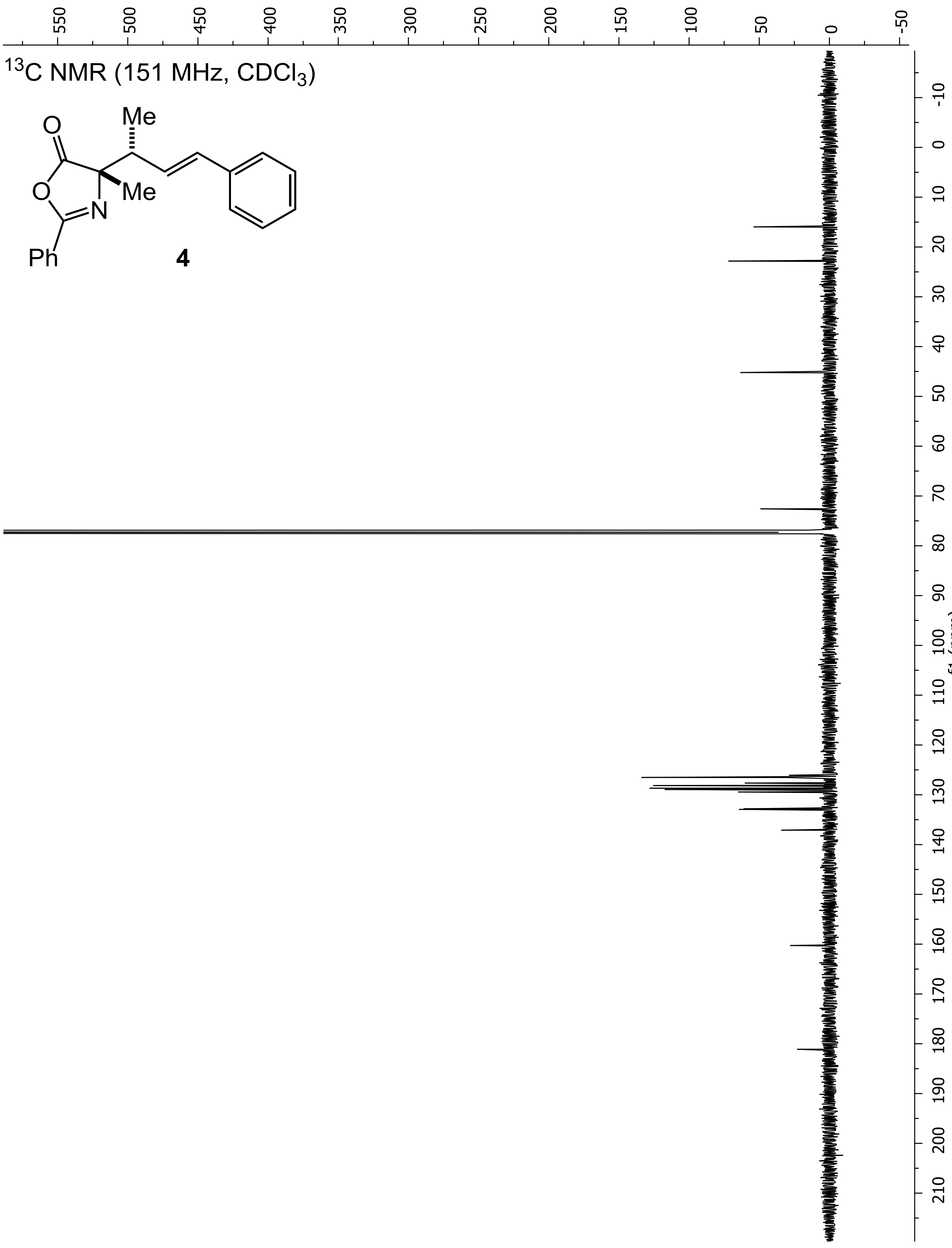

13C NMR (151 MHz, CDCl3)
Me
O
O
N
Me
Ph
4
f1 (ppm)

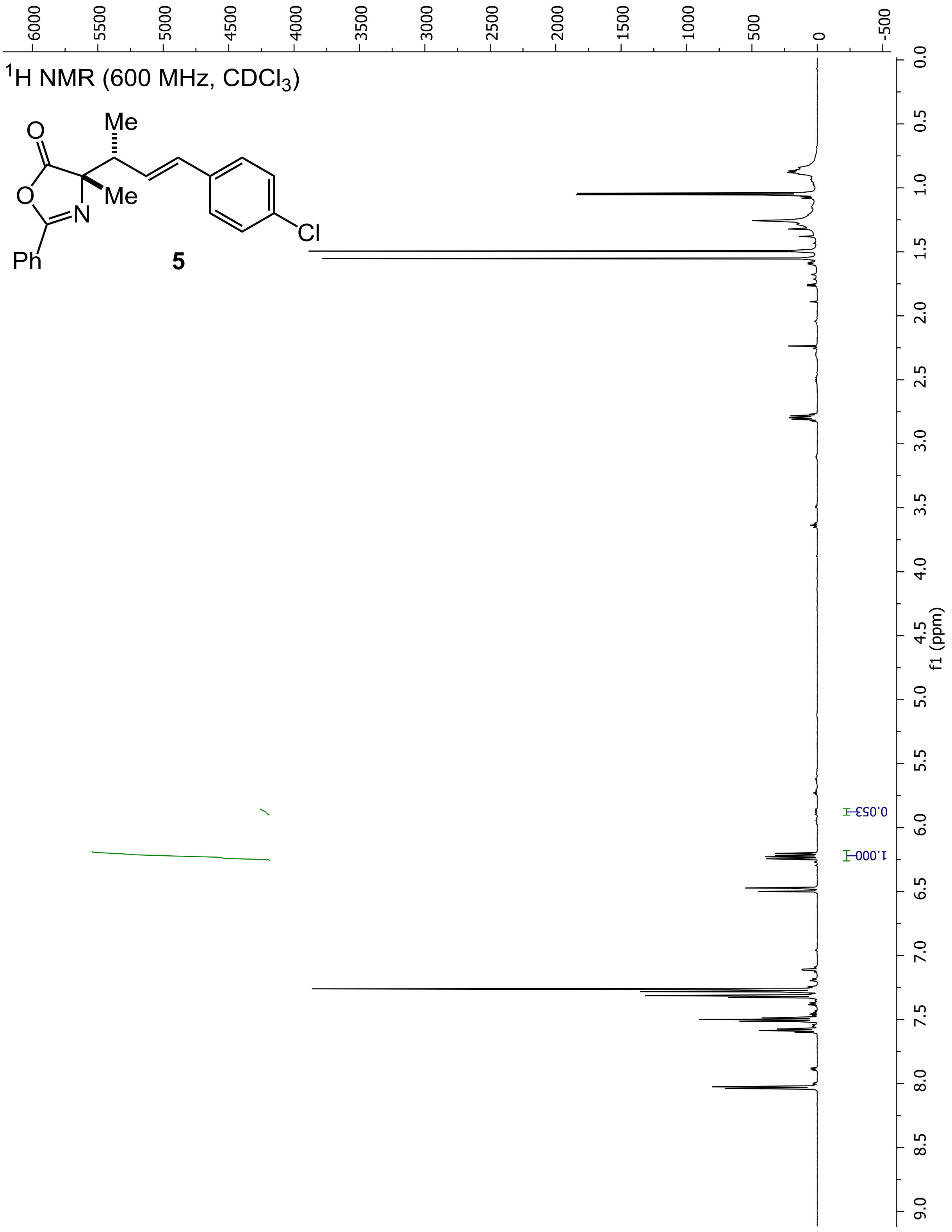

1H NMR (600 MHz, CDCl3)
Me
O
O
Me
N
Cl
Ph
5
f1 (ppm)
0.053
1.000

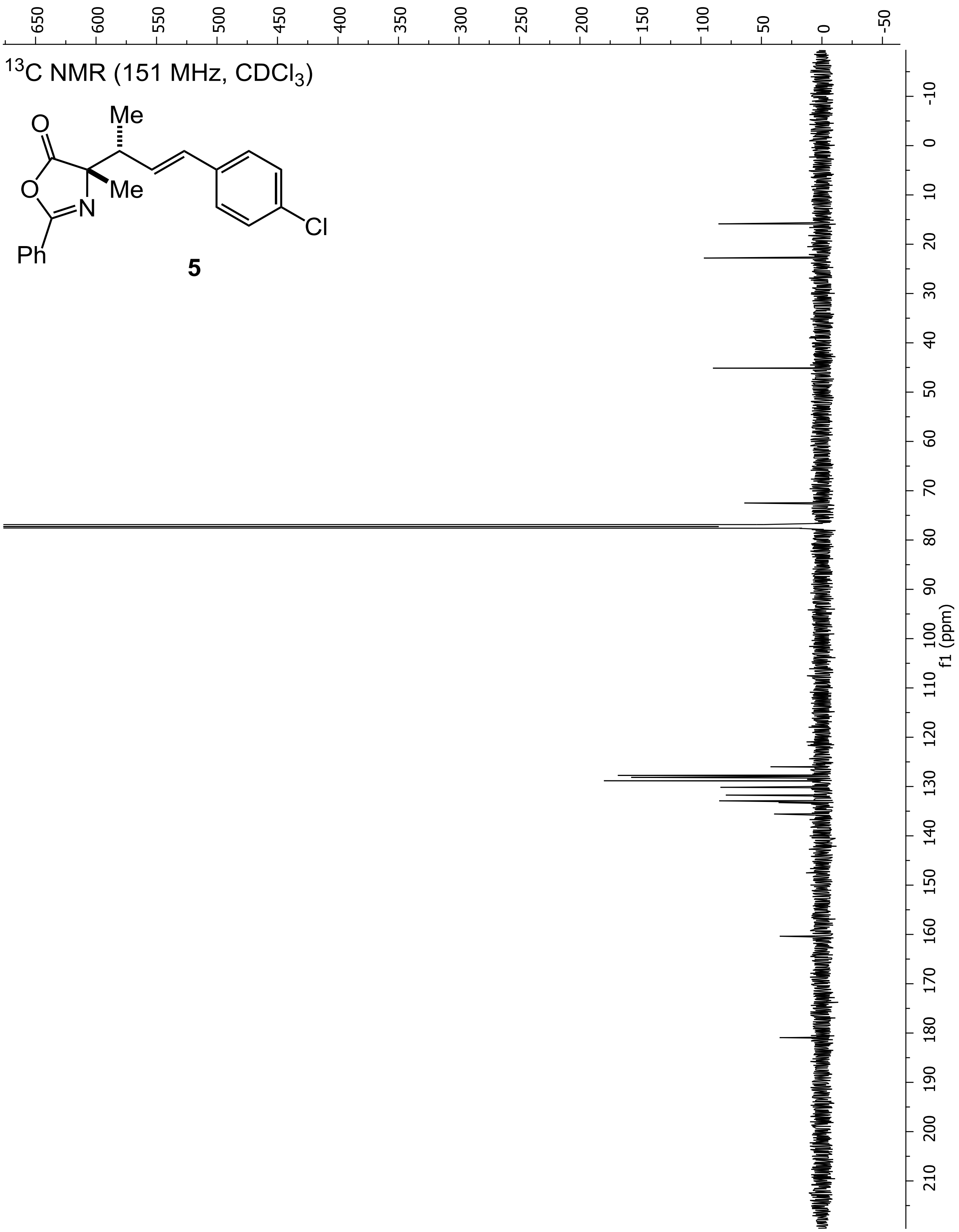
13C NMR (151 MHz, CDCl3)
Me
O
O
Me
N
Cl
Ph
5
f1 (ppm)

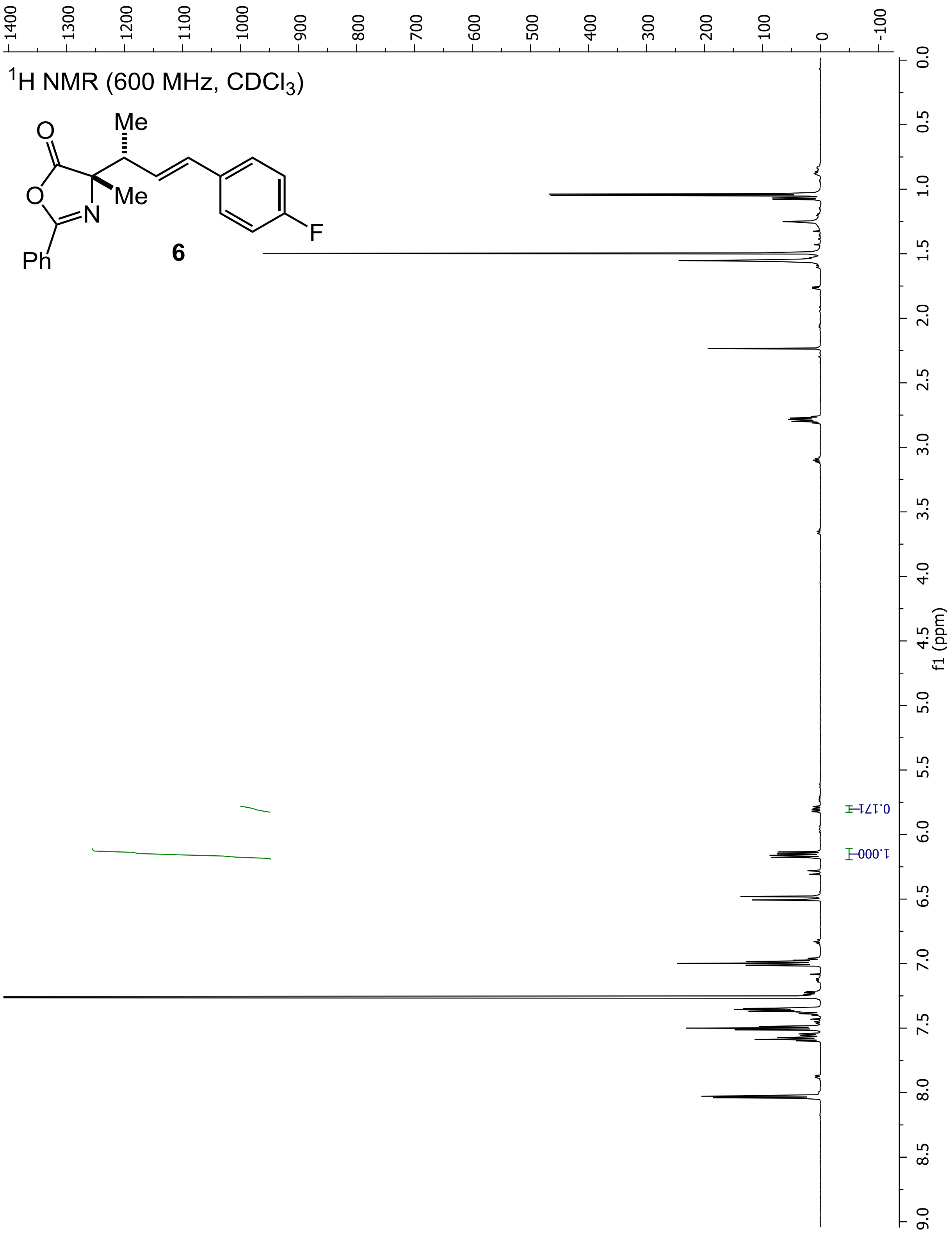
^{1}H NMR (600 MHz, CDCl$_3$)
6
f1 (ppm)
0.171
1.000

^{13}C NMR (151 MHz, $CDCl_3$)

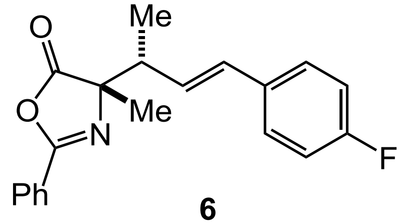

6

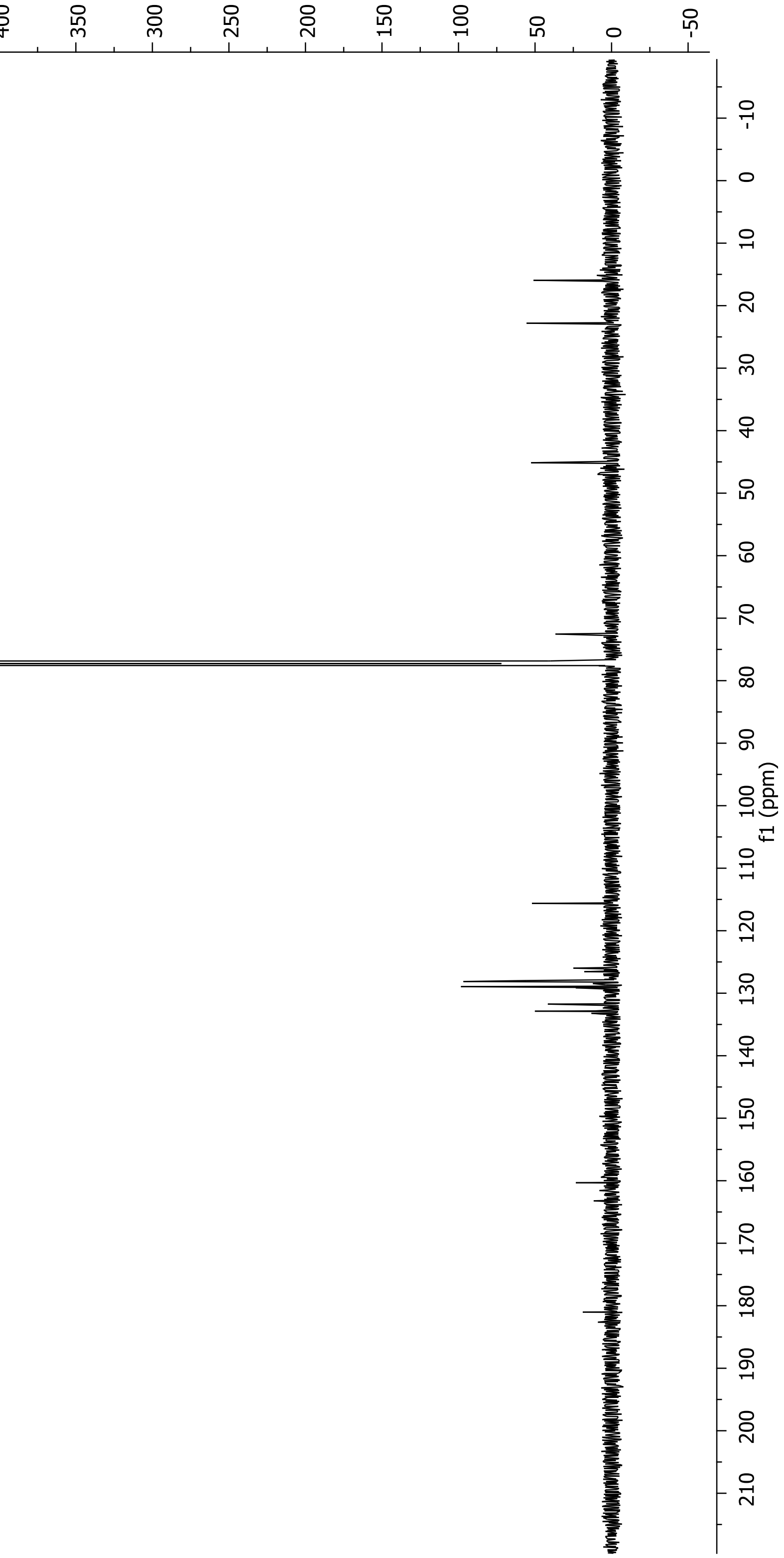

^{1}H NMR (500 MHz, $CDCl_3$)

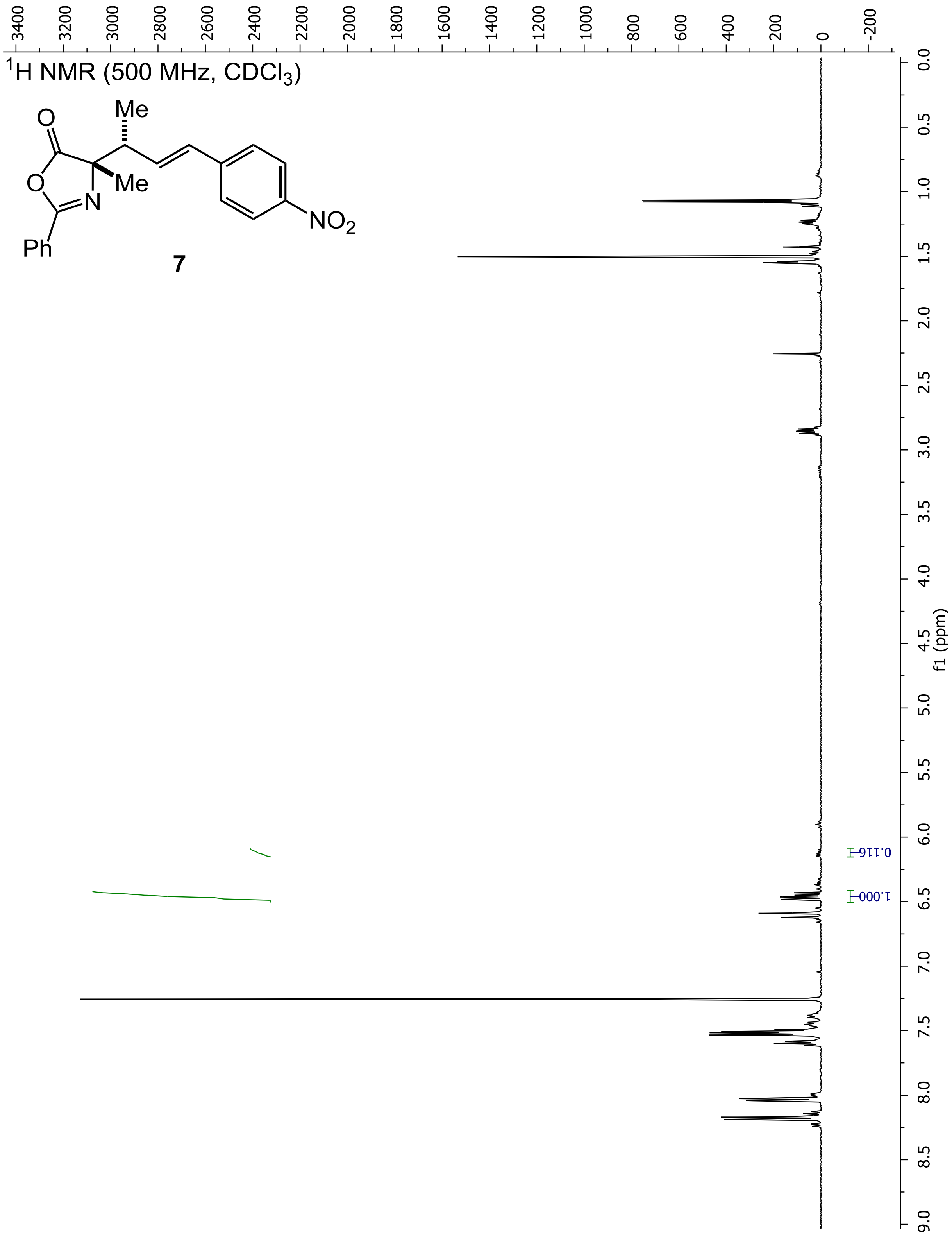

7

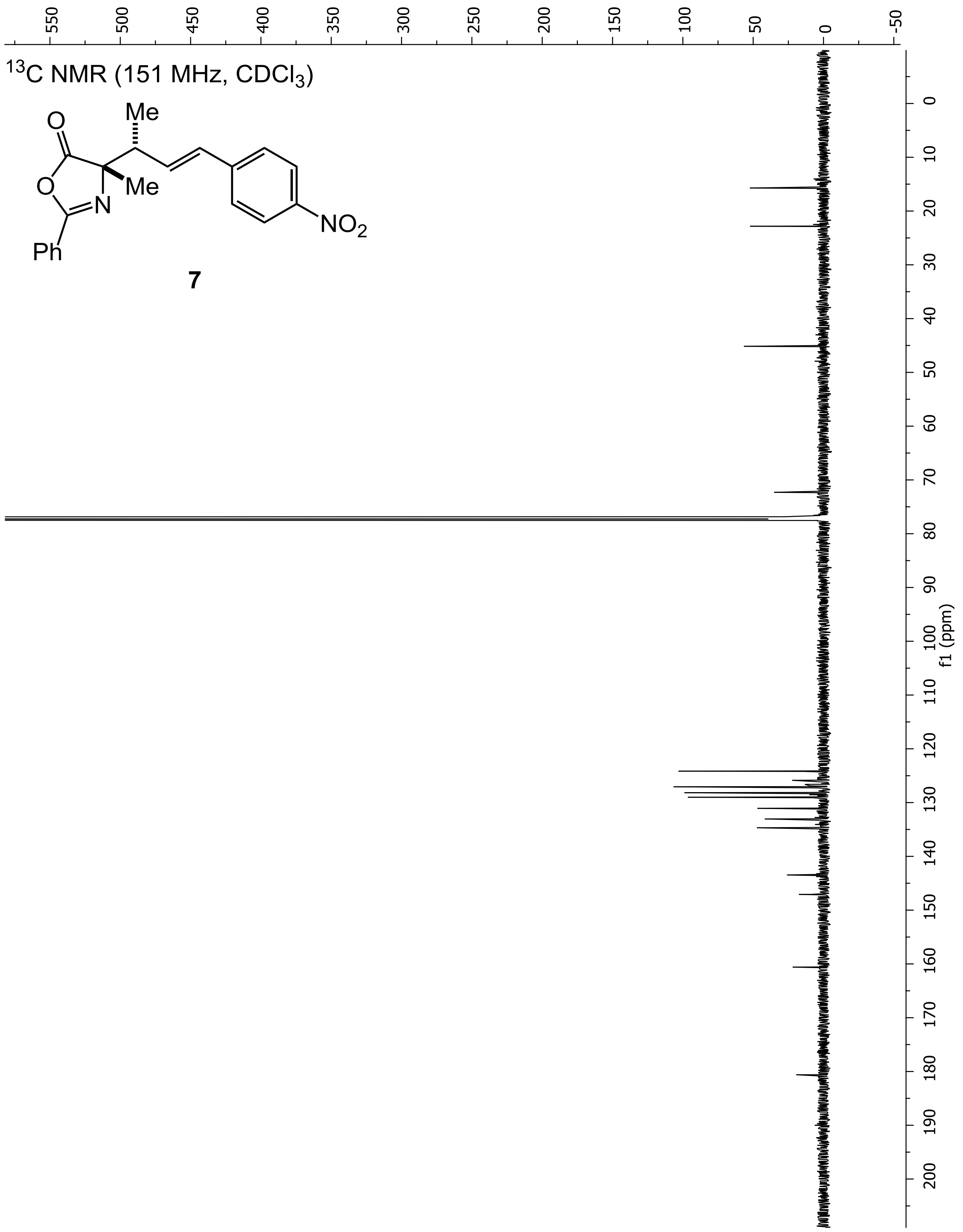

^{13}C NMR (151 MHz, CDCl$_3$)
Me
O
O
Me
N
NO$_2$
Ph
7
f1 (ppm)

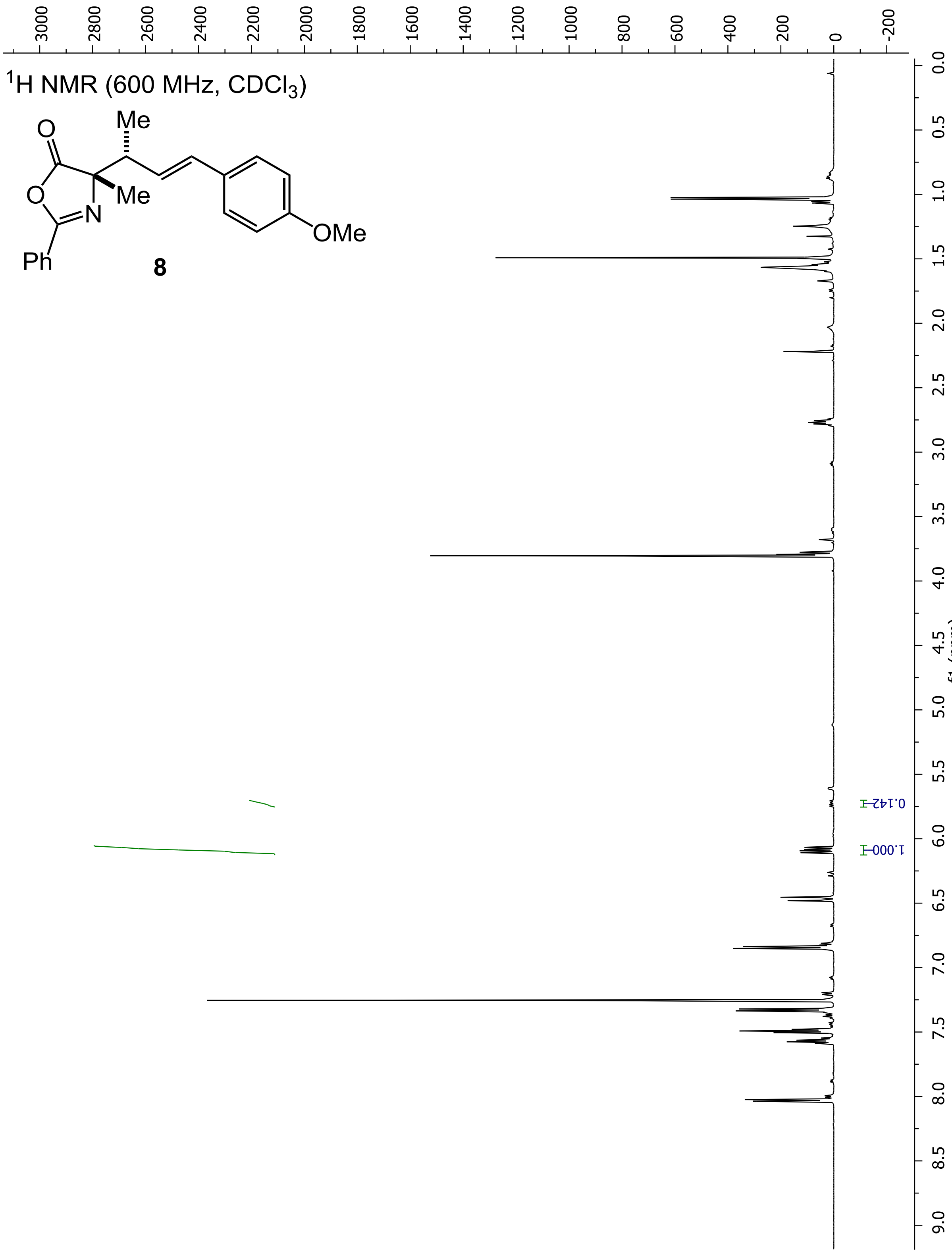

^{1}H NMR (600 MHz, $CDCl_3$)
Me
O
O
Me
N
Ph
OMe
8
f1 (ppm)
0.142
1.000

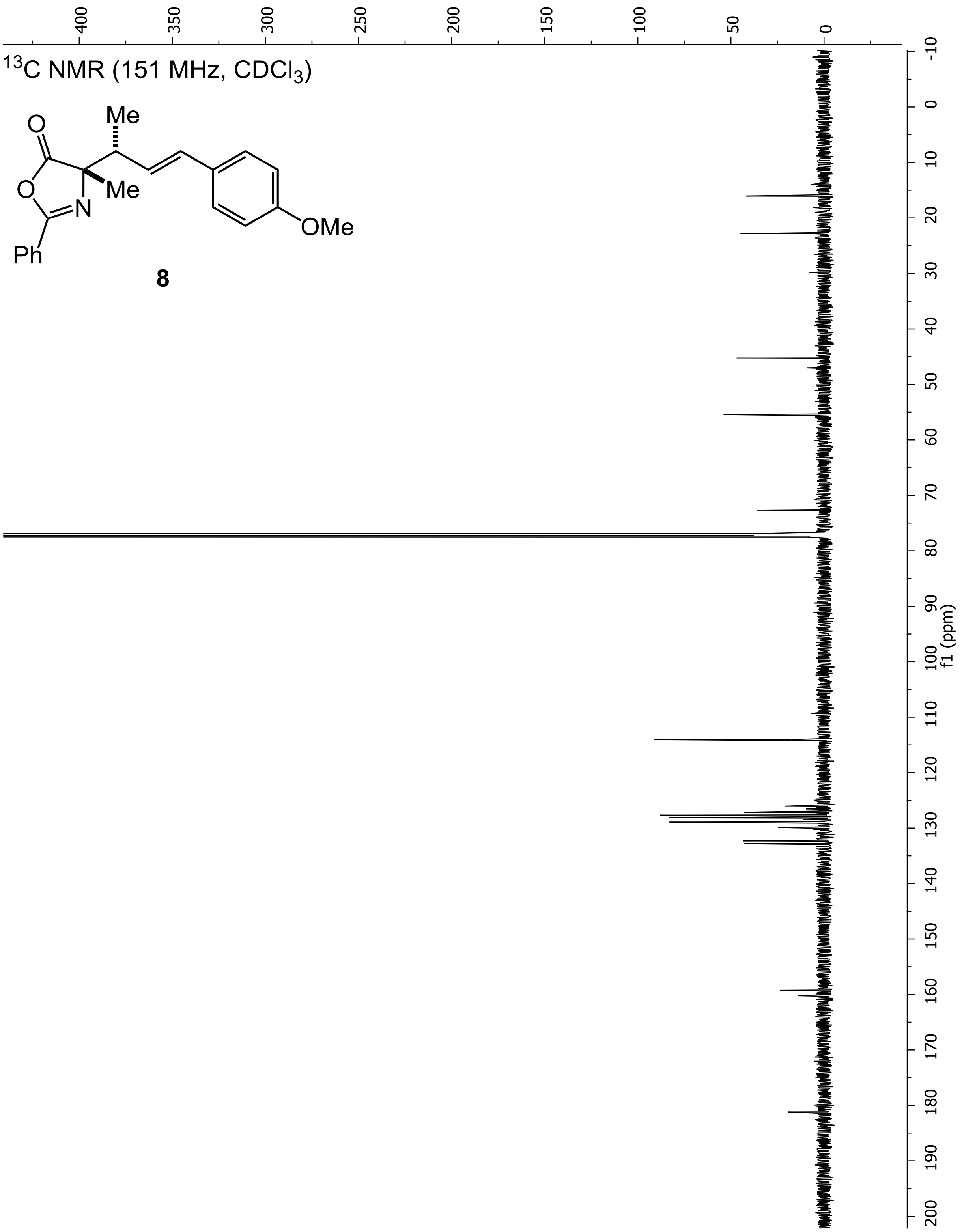
^{13}C NMR (151 MHz, $CDCl_3$)
O
Me
O
Me
N
OMe
Ph
8
f1 (ppm)

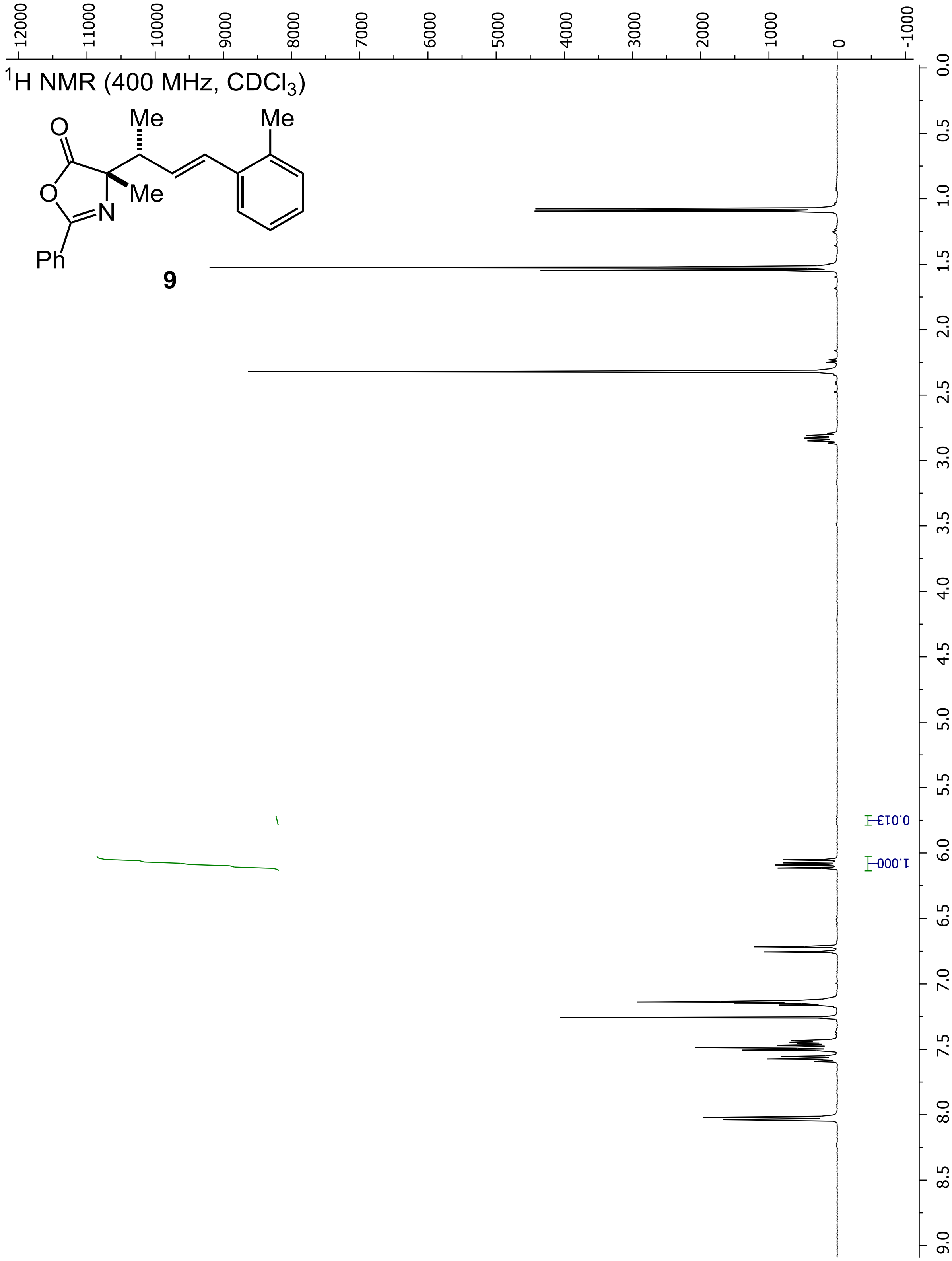

^1H NMR (400 MHz, $CDCl_3$)
O
Me
Me
O
Me
N
Ph
9
f1 (ppm)

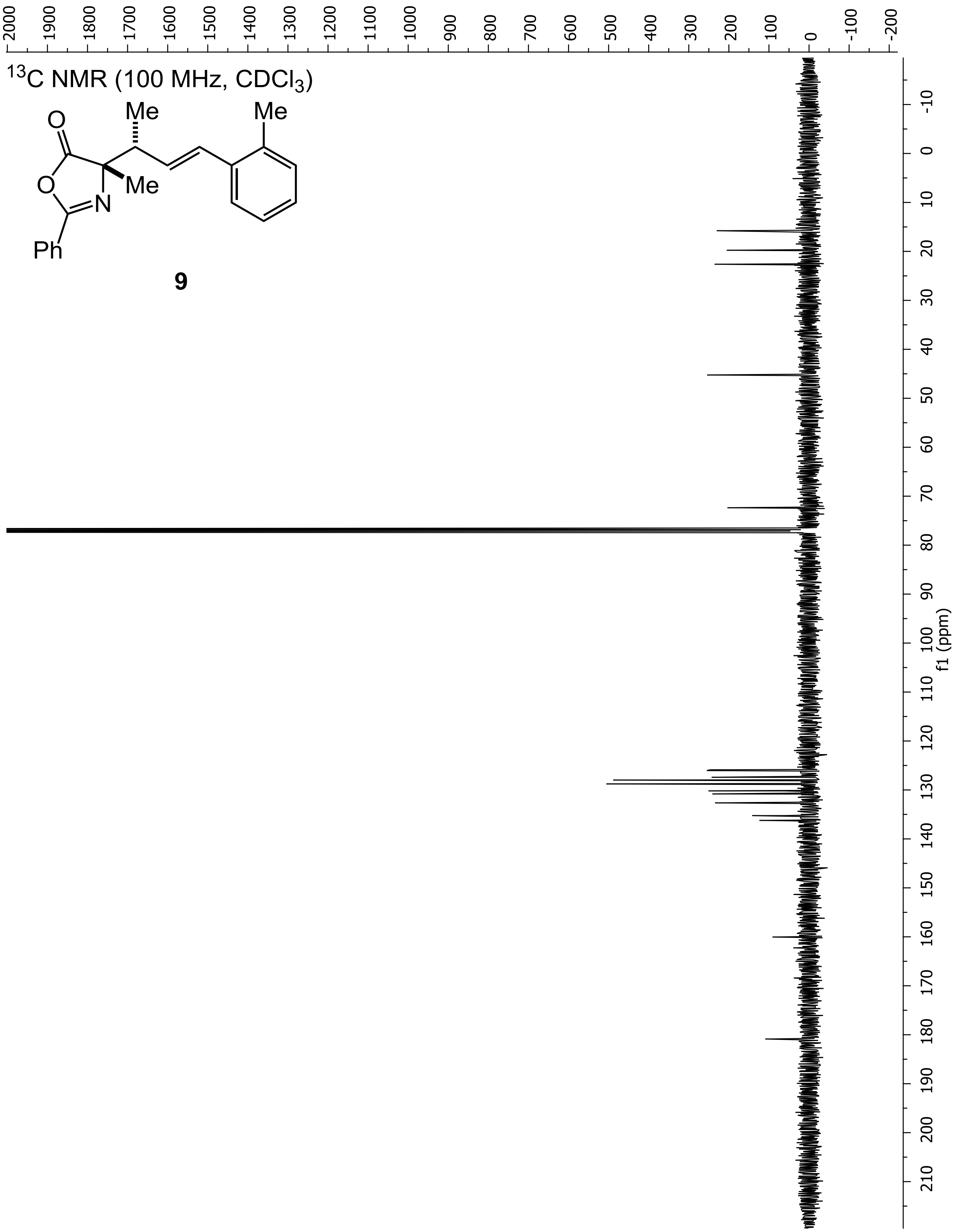

^{13}C NMR (100 MHz, CDCl$_3$)
O
Me
Me
O
Me
N
Ph
9
f1 (ppm)

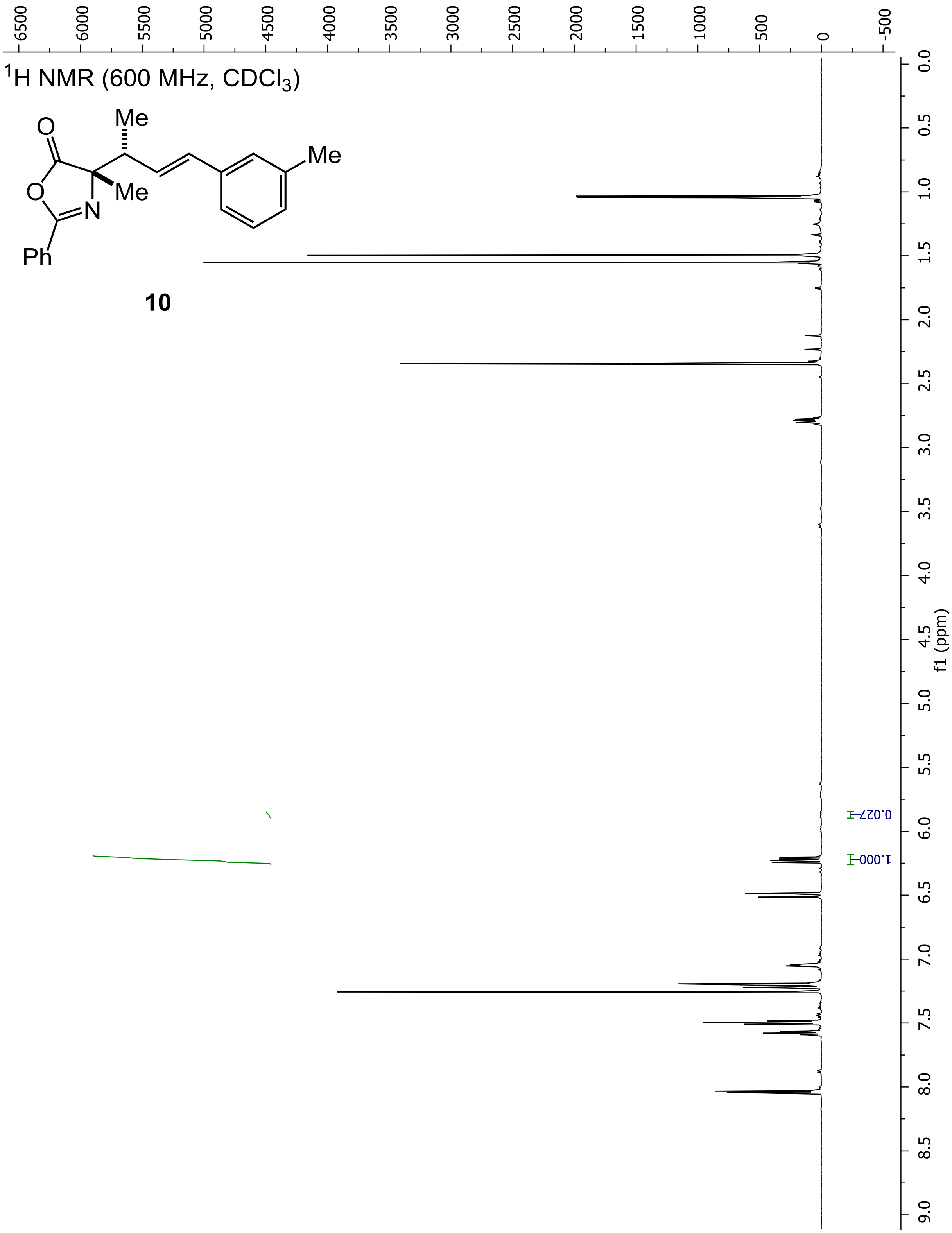

^{1}H NMR (600 MHz, $CDCl_3$)
Me
Me
Me
O
O
N
Ph
10
f1 (ppm)
0.027
1.000

^{13}C NMR (151 MHz, $CDCl_3$)

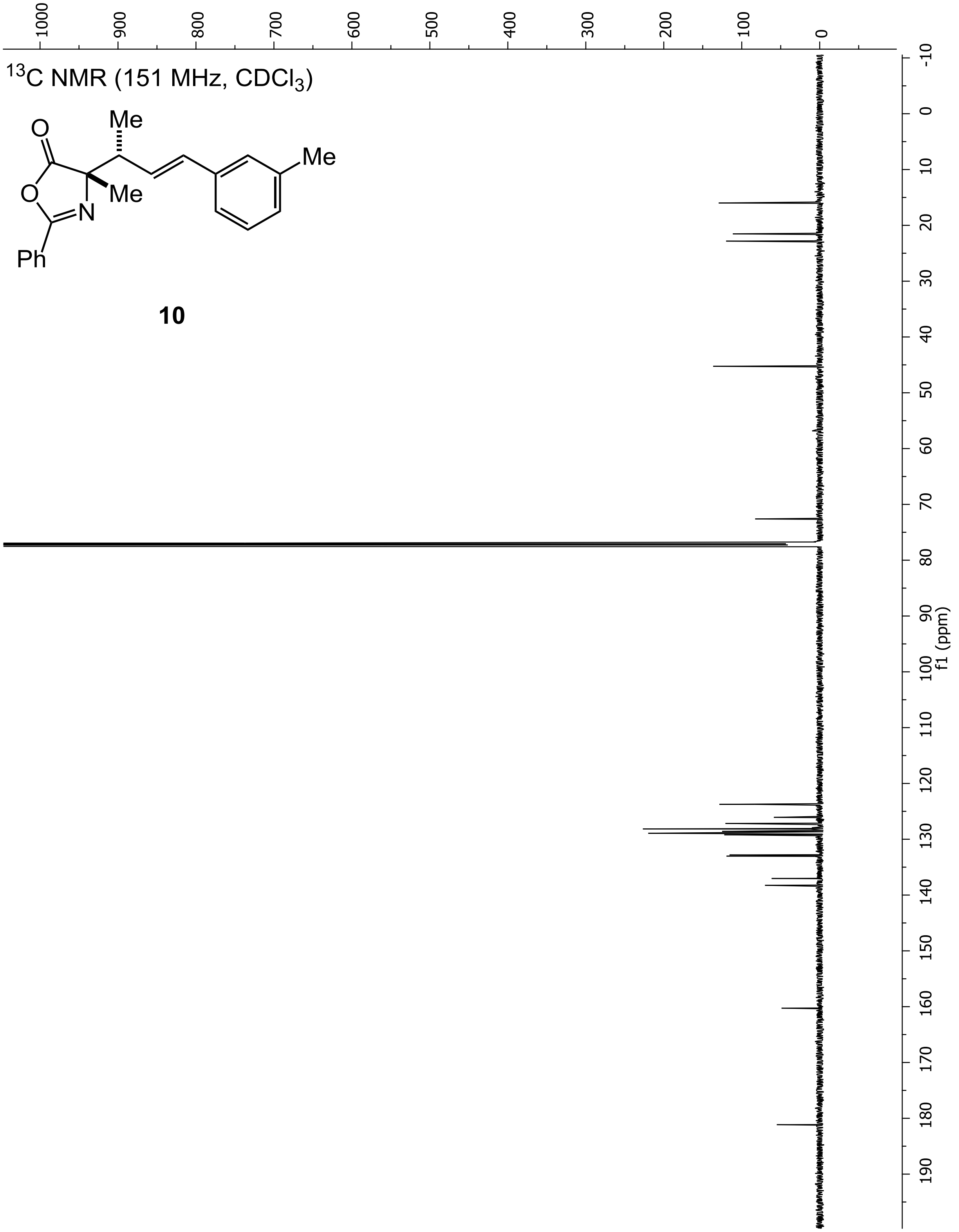

10

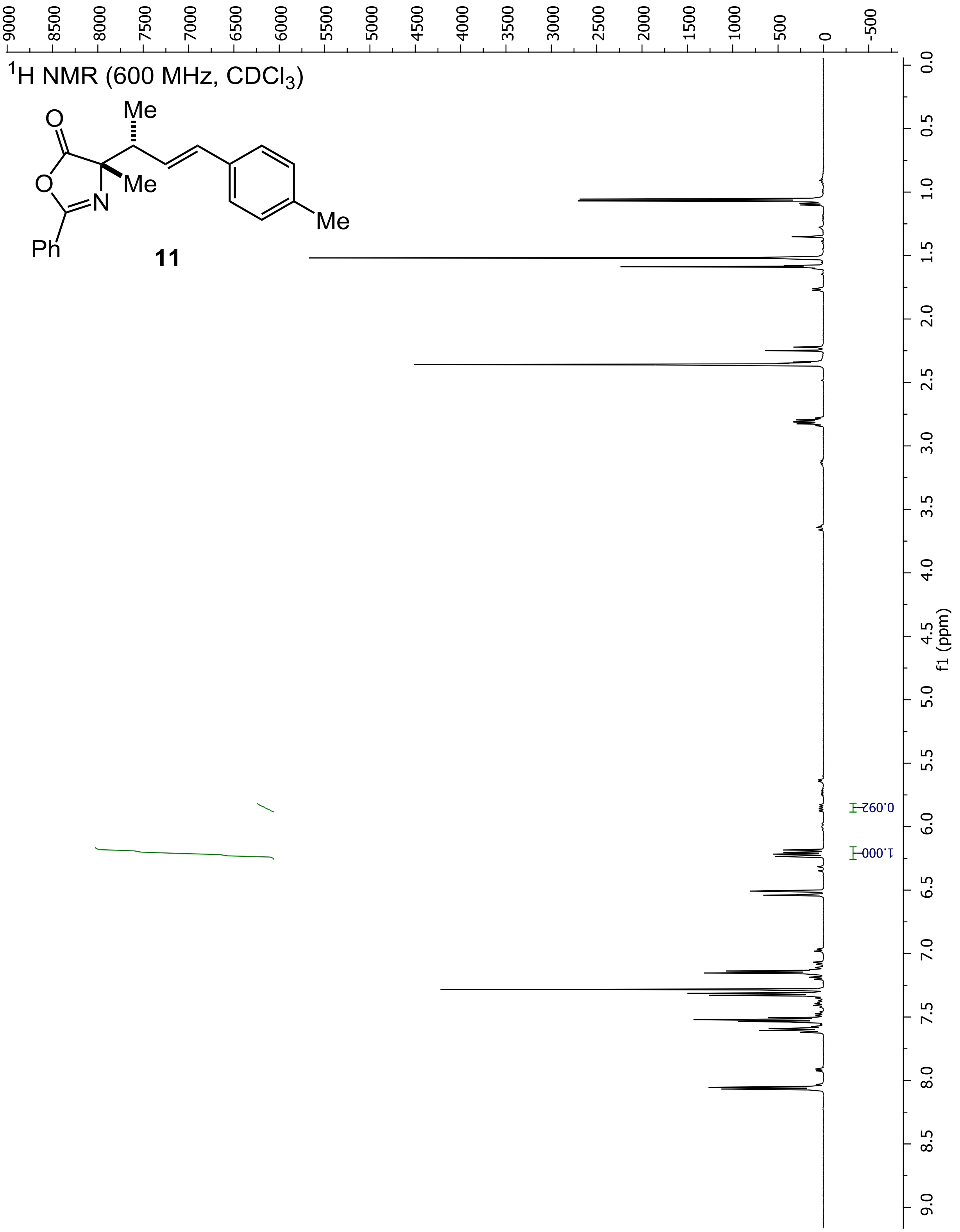

^1H NMR (600 MHz, $CDCl_3$)
Me
O
O
Me
N
Ph
Me
11
f1 (ppm)
0.092
1.000

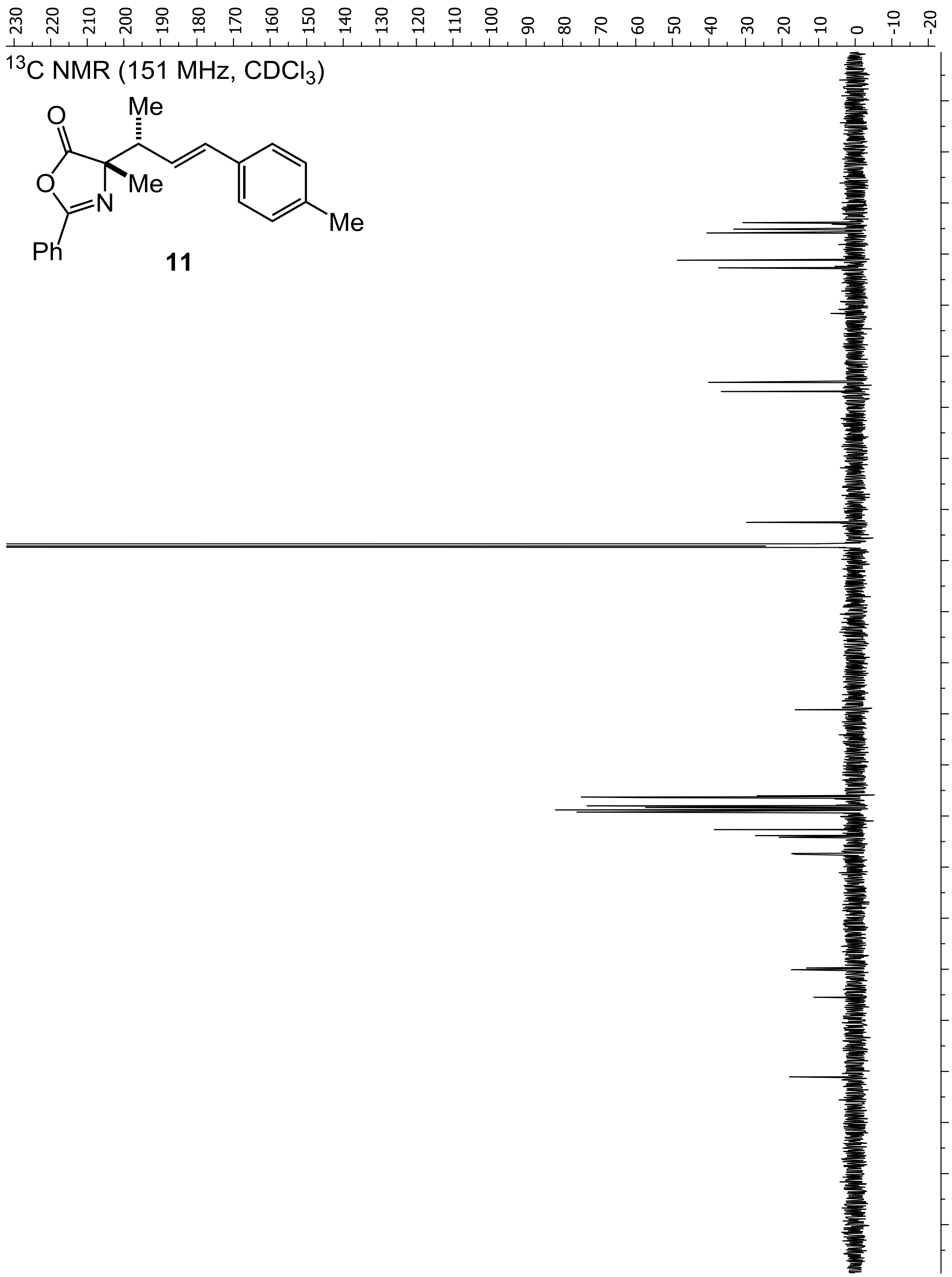

^{13}C NMR (151 MHz, CDCl$_3$)
Me
O
O
Me
N
Ph
Me
11
f1 (ppm)

^{1}H NMR (600 MHz, $CDCl_3$)

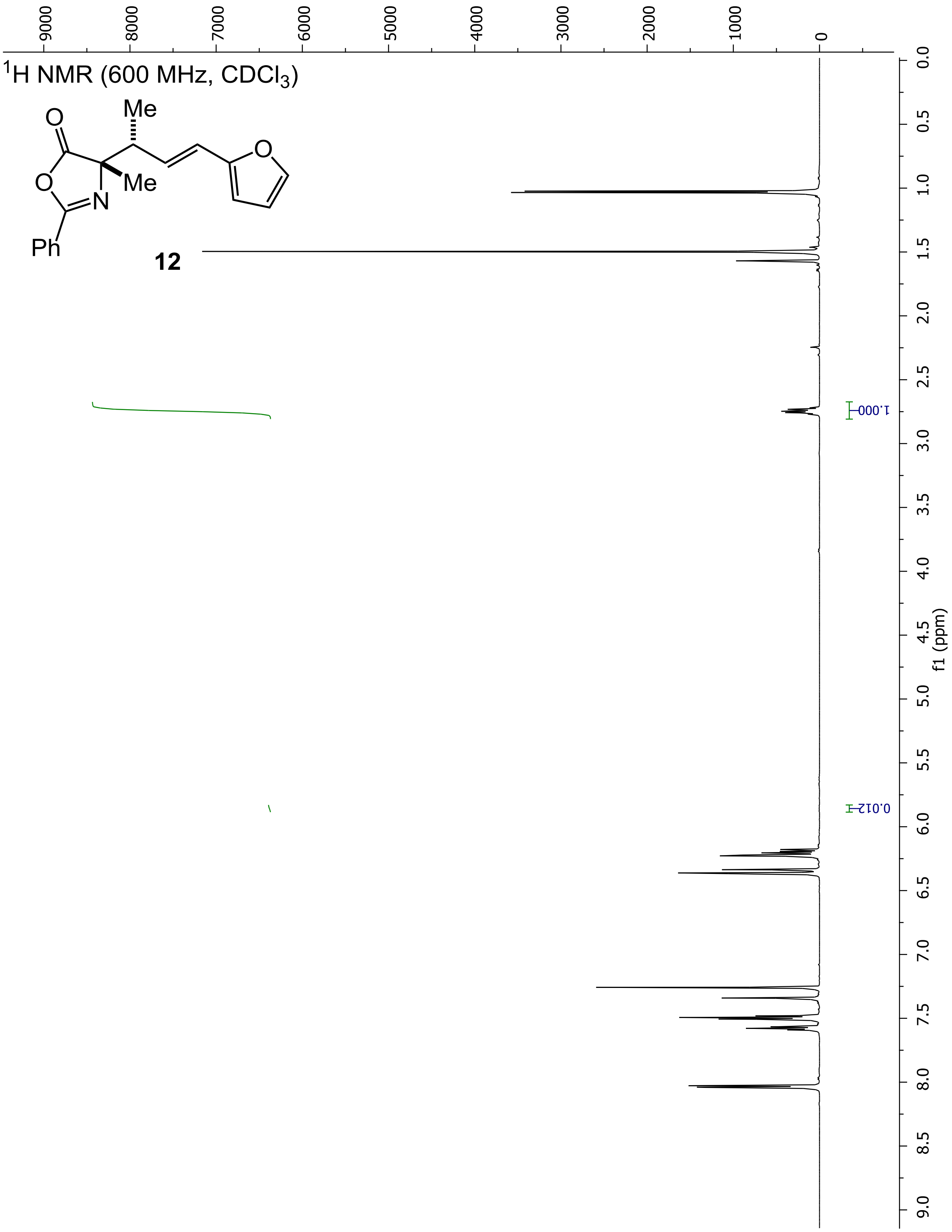

12

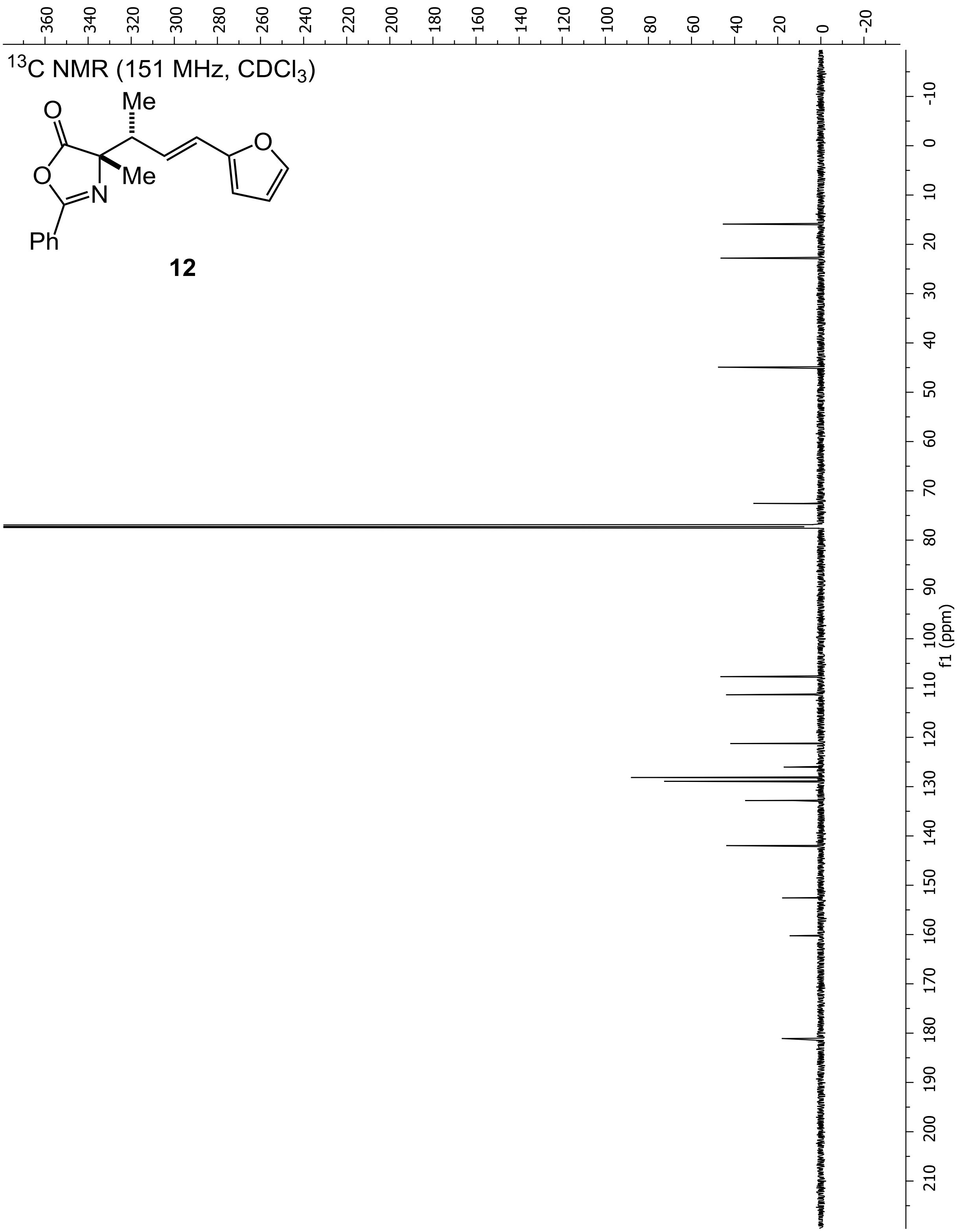

13C NMR (151 MHz, CDCl3)
Me
O
O
Me
N
O
Ph
12
f1 (ppm)

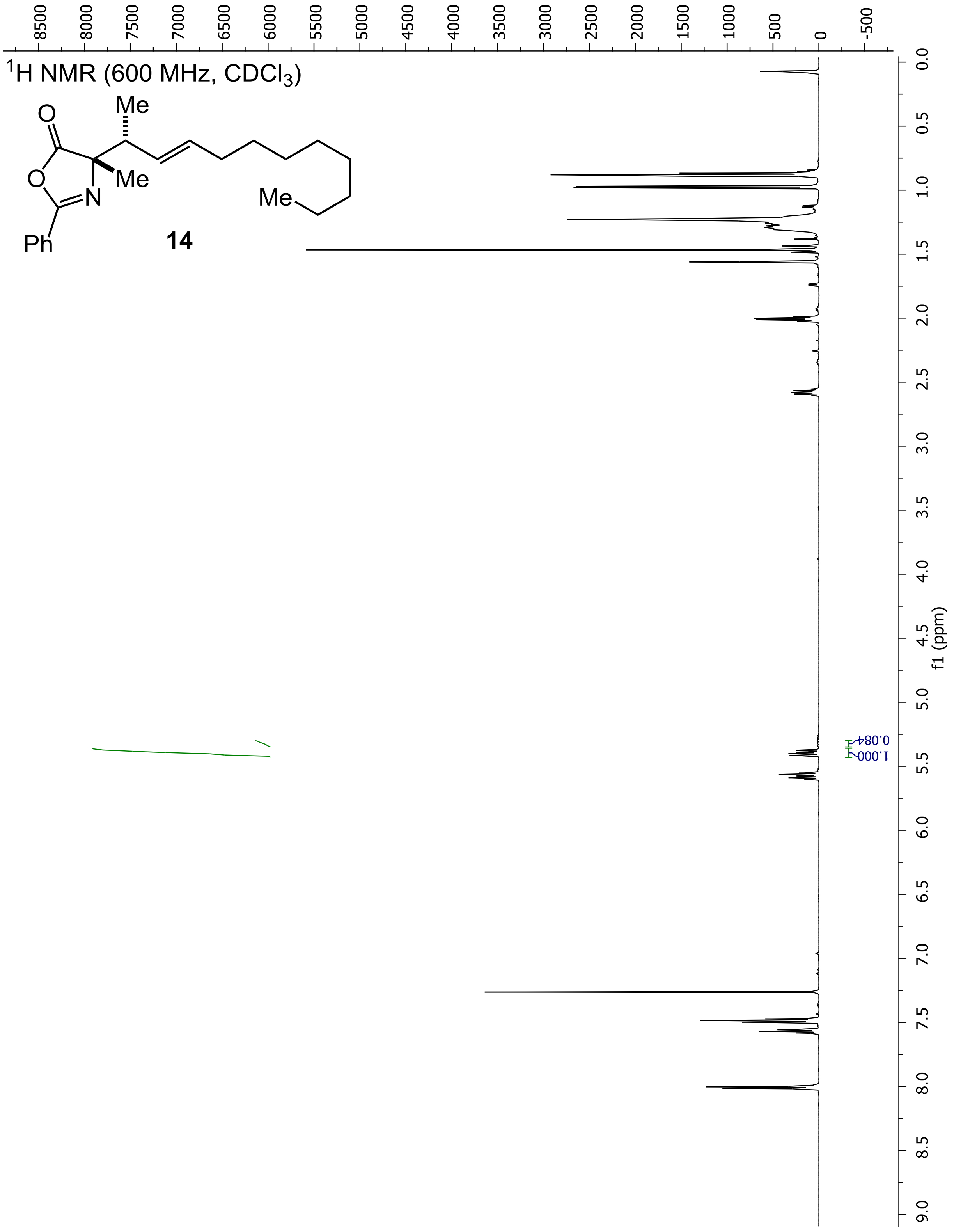

^{1}H NMR (600 MHz, CDCl$_3$)
14
f1 (ppm)

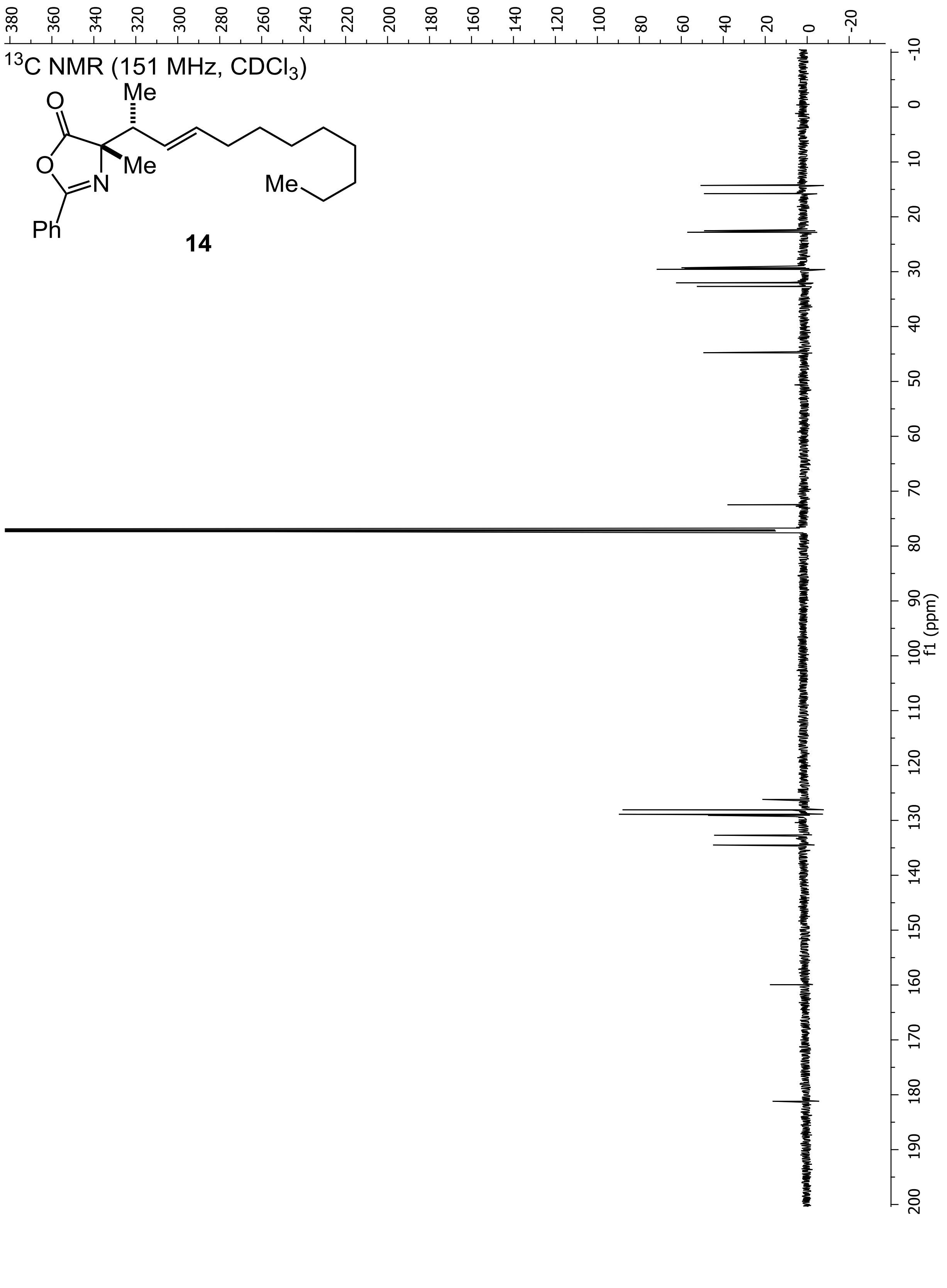

13C NMR (151 MHz, CDCl3)
14
f1 (ppm)

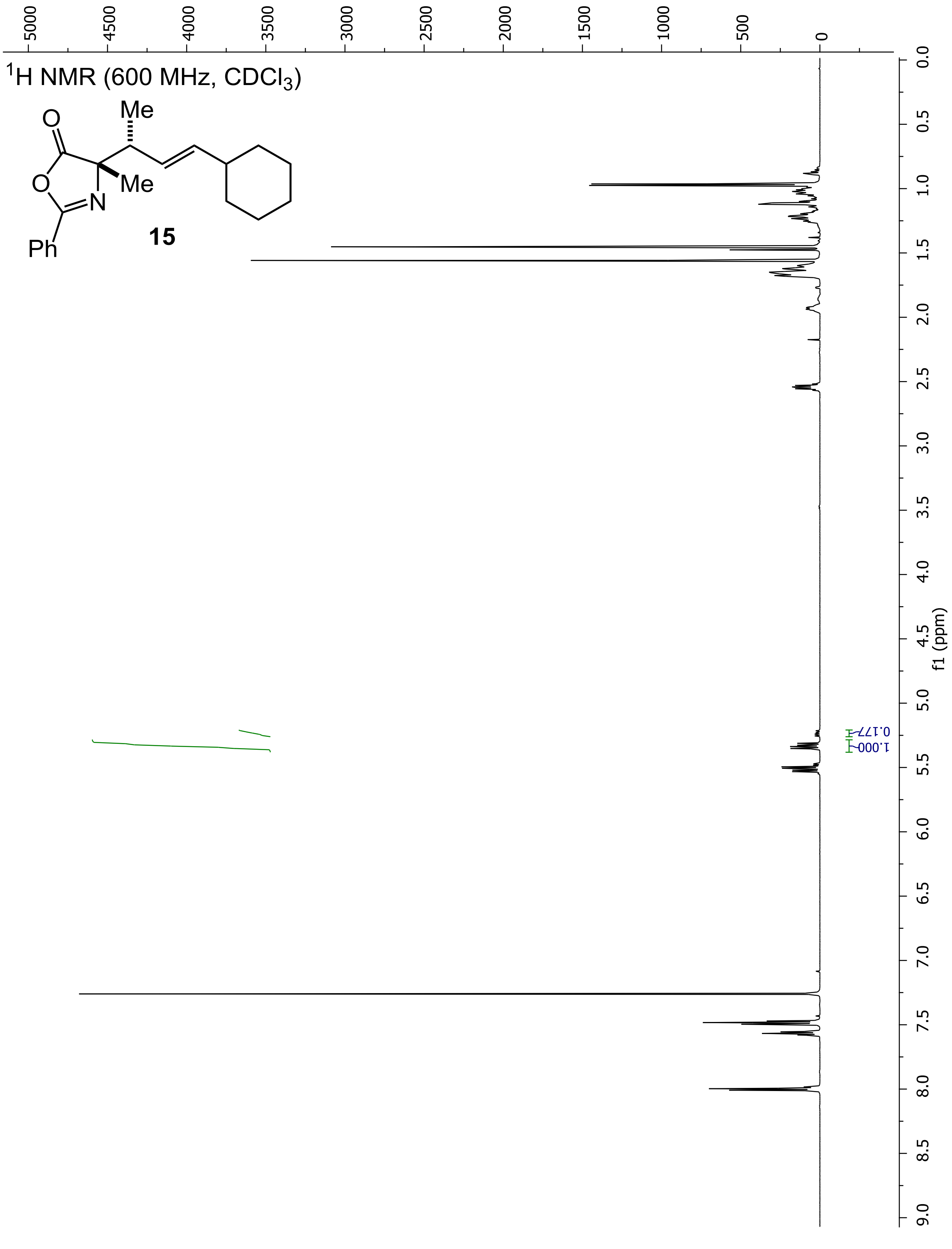

^{1}H NMR (600 MHz, $CDCl_3$)
15
f1 (ppm)

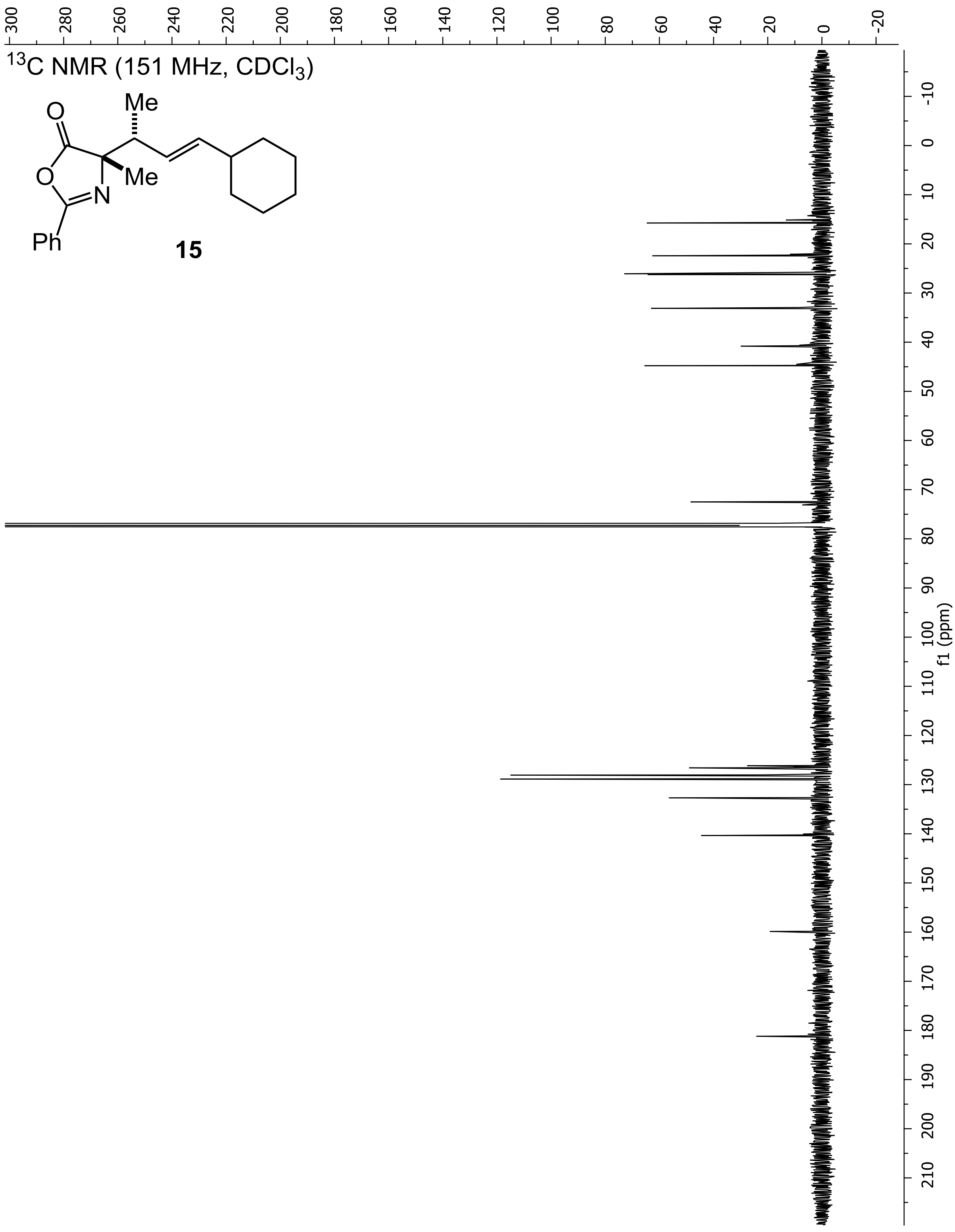

^{13}C NMR (151 MHz, CDCl$_3$)
O
Me
O
N
Me
Ph
15
f1 (ppm)

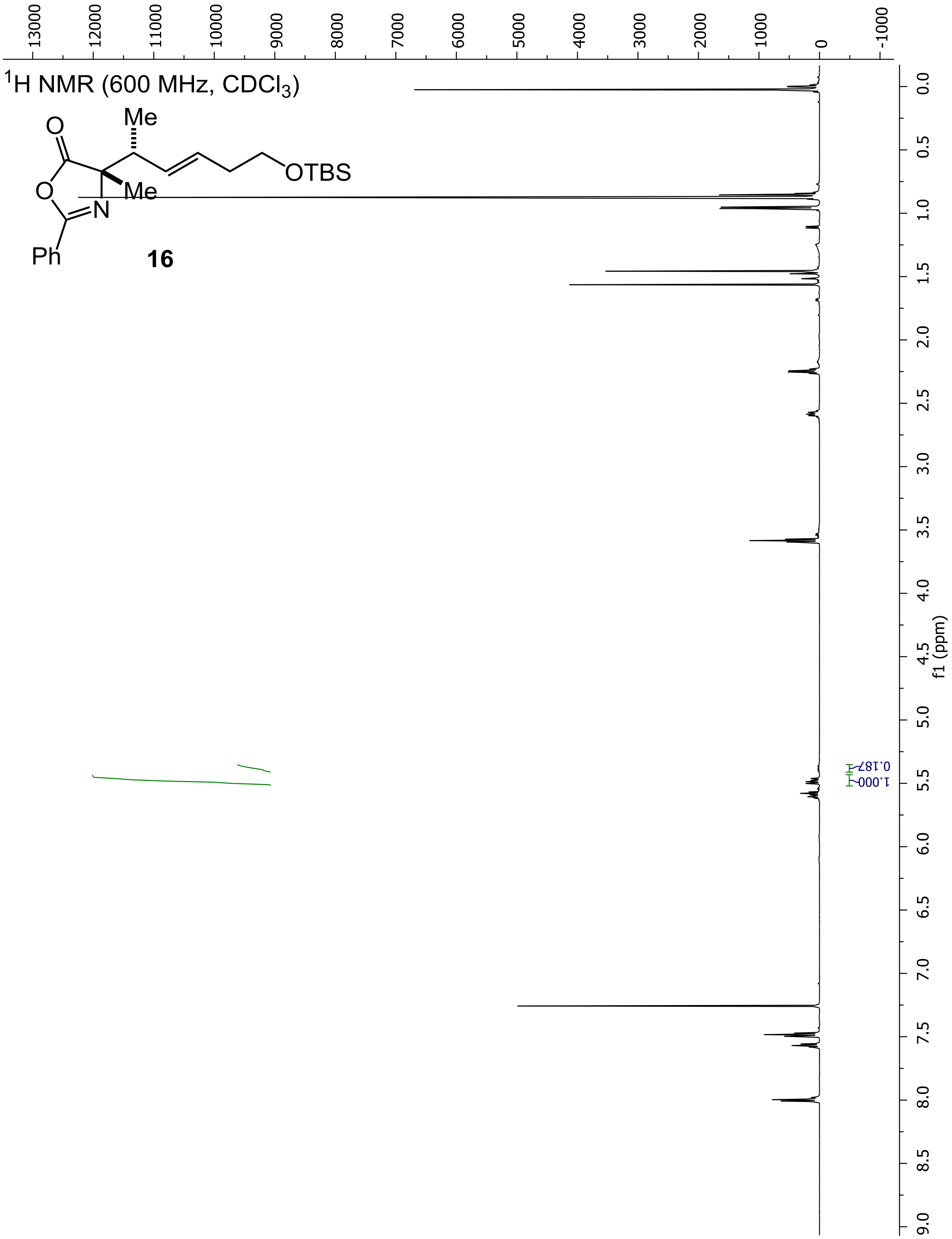

^1H NMR (600 MHz, $CDCl_3$)
Me
OTBS
Me
Ph
16
f1 (ppm)

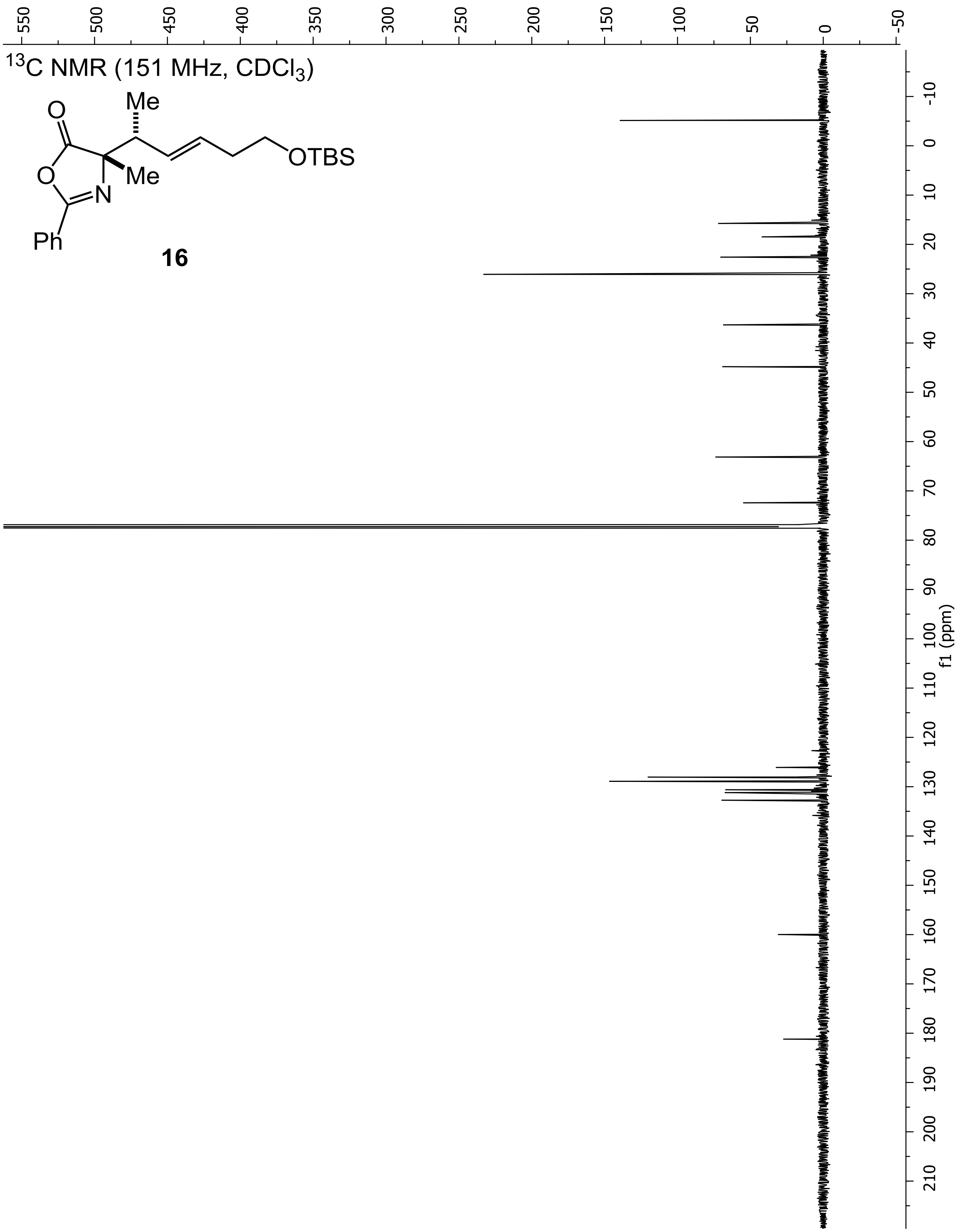

13C NMR (151 MHz, CDCl3)
Me
O
O
Me
N
OTBS
Ph
16
f1 (ppm)

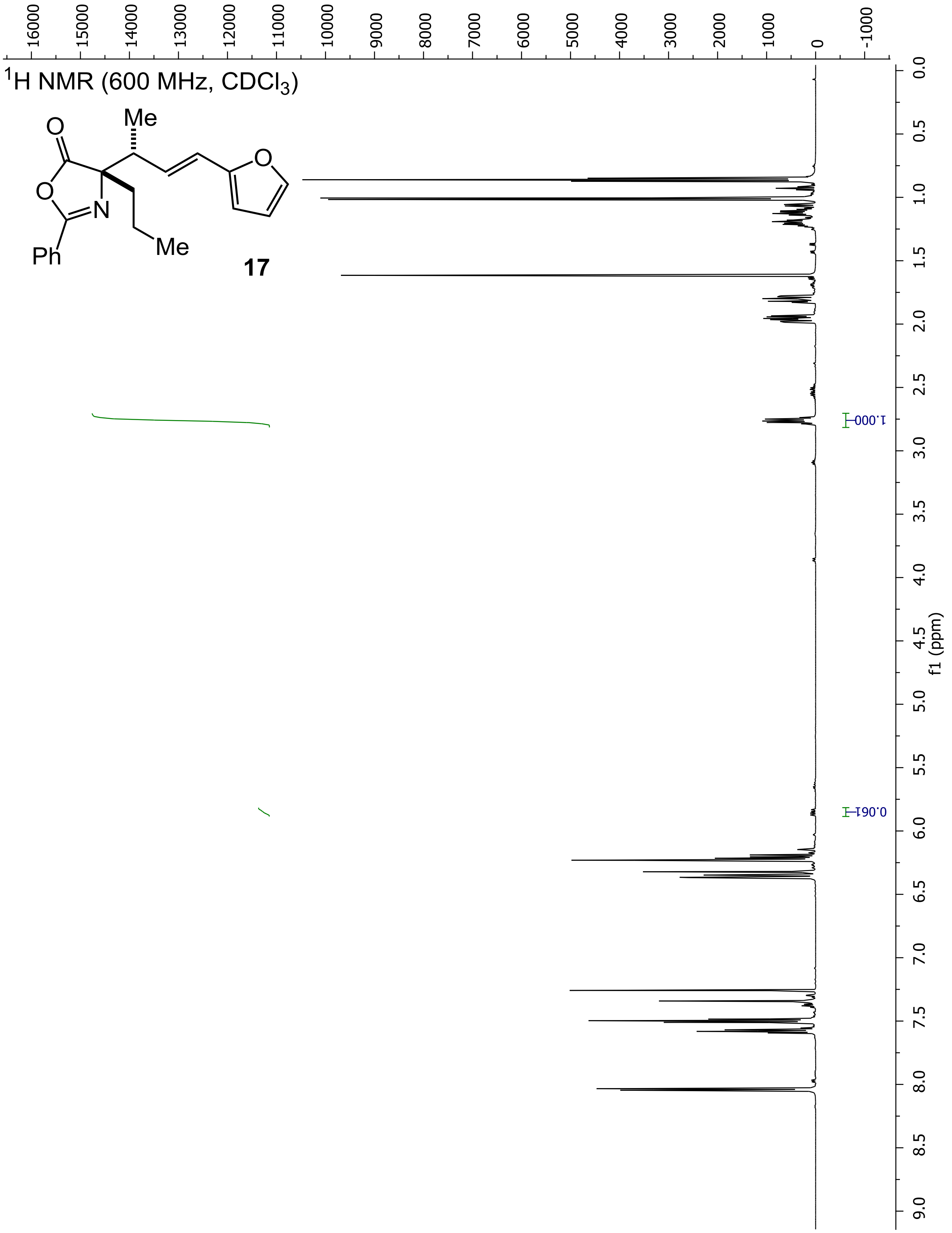

^{1}H NMR (600 MHz, $CDCl_3$)
17
f1 (ppm)
1.000
0.061

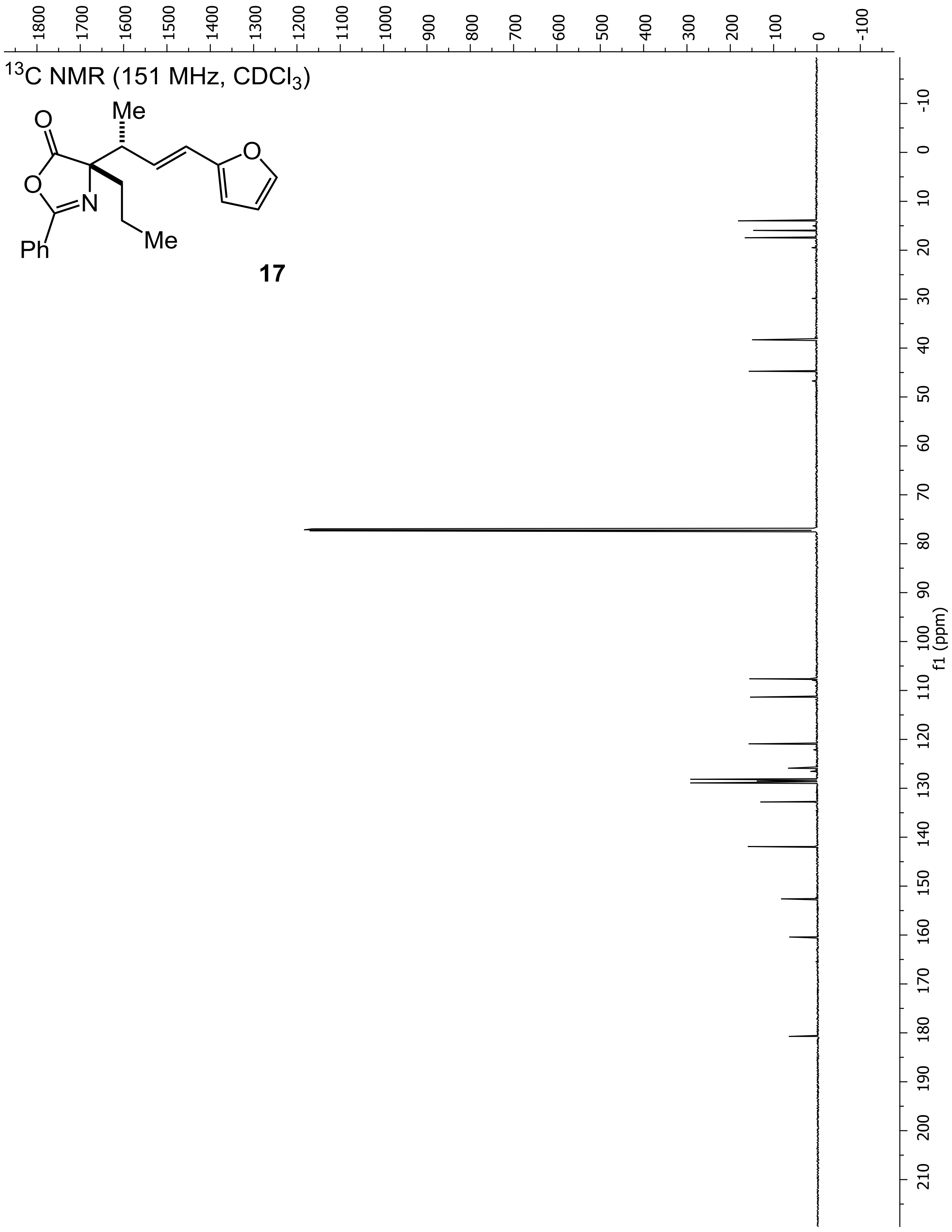
^{13}C NMR (151 MHz, $CDCl_3$)
O
Me
O
O
N
Ph
Me
17
f1 (ppm)

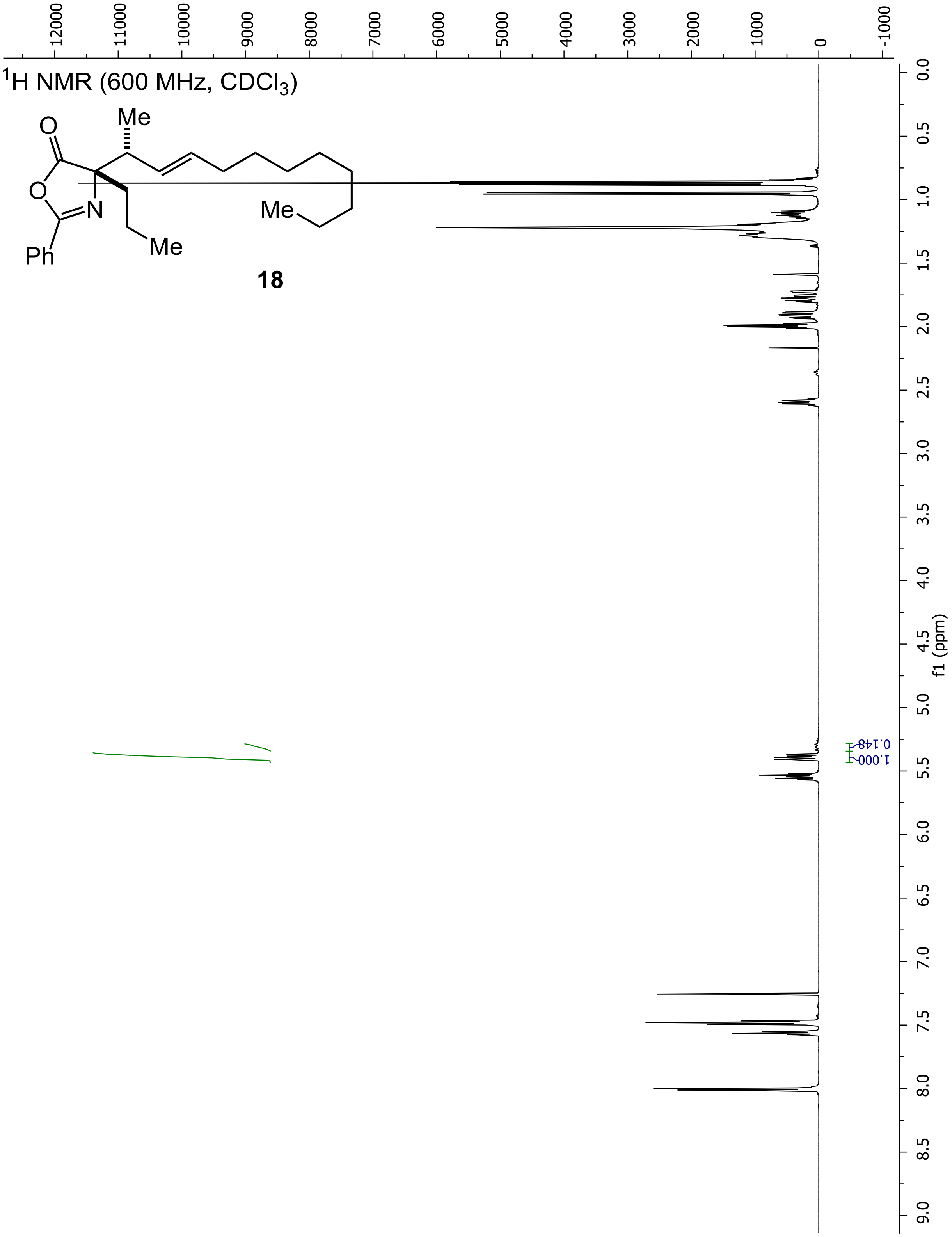

^{1}H NMR (600 MHz, $CDCl_3$)
O
Me
O
N
Me
Ph
Me
18
f1 (ppm)
1.000
0.148

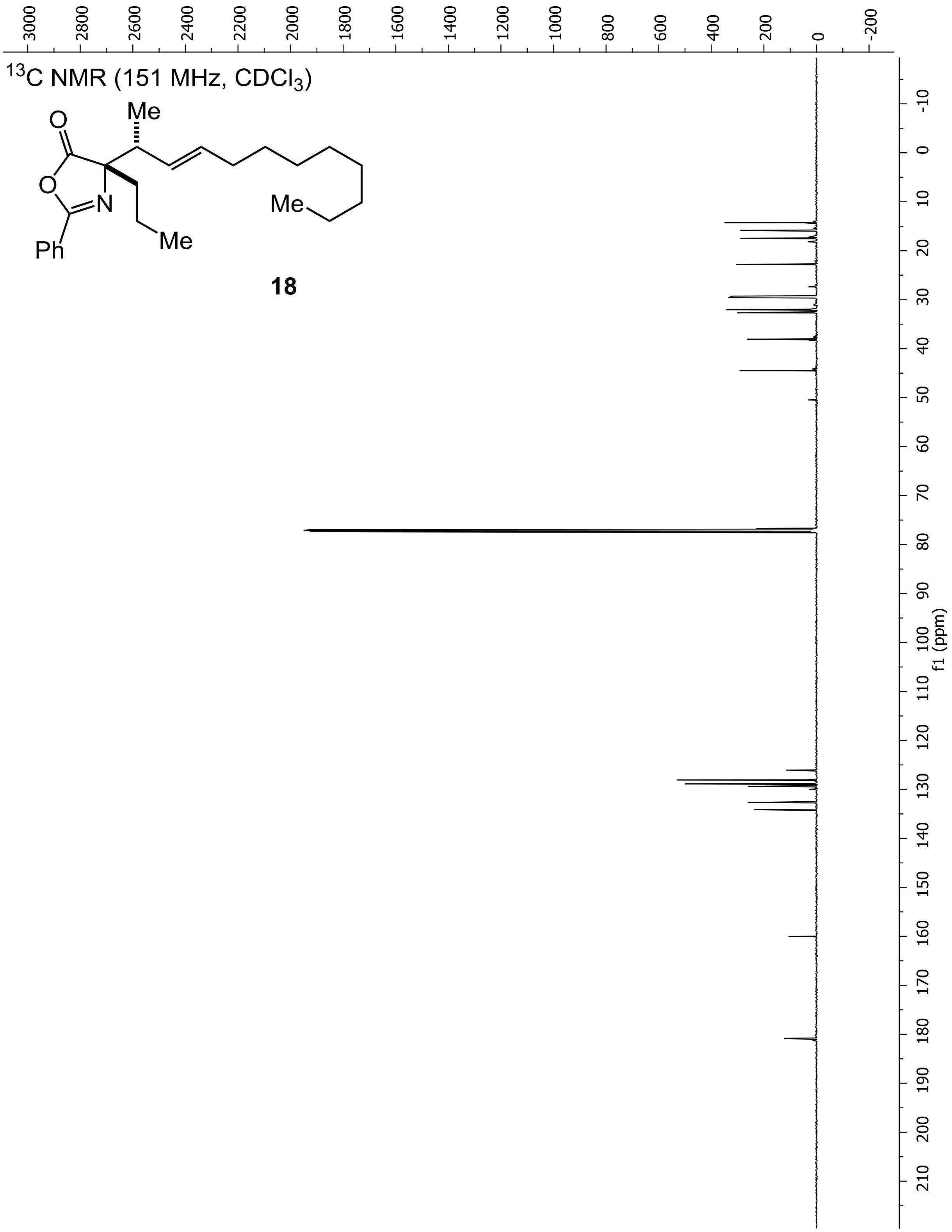
^{13}C NMR (151 MHz, $CDCl_3$)
O
Me
O
N
Me
Me
Ph
18
f1 (ppm)

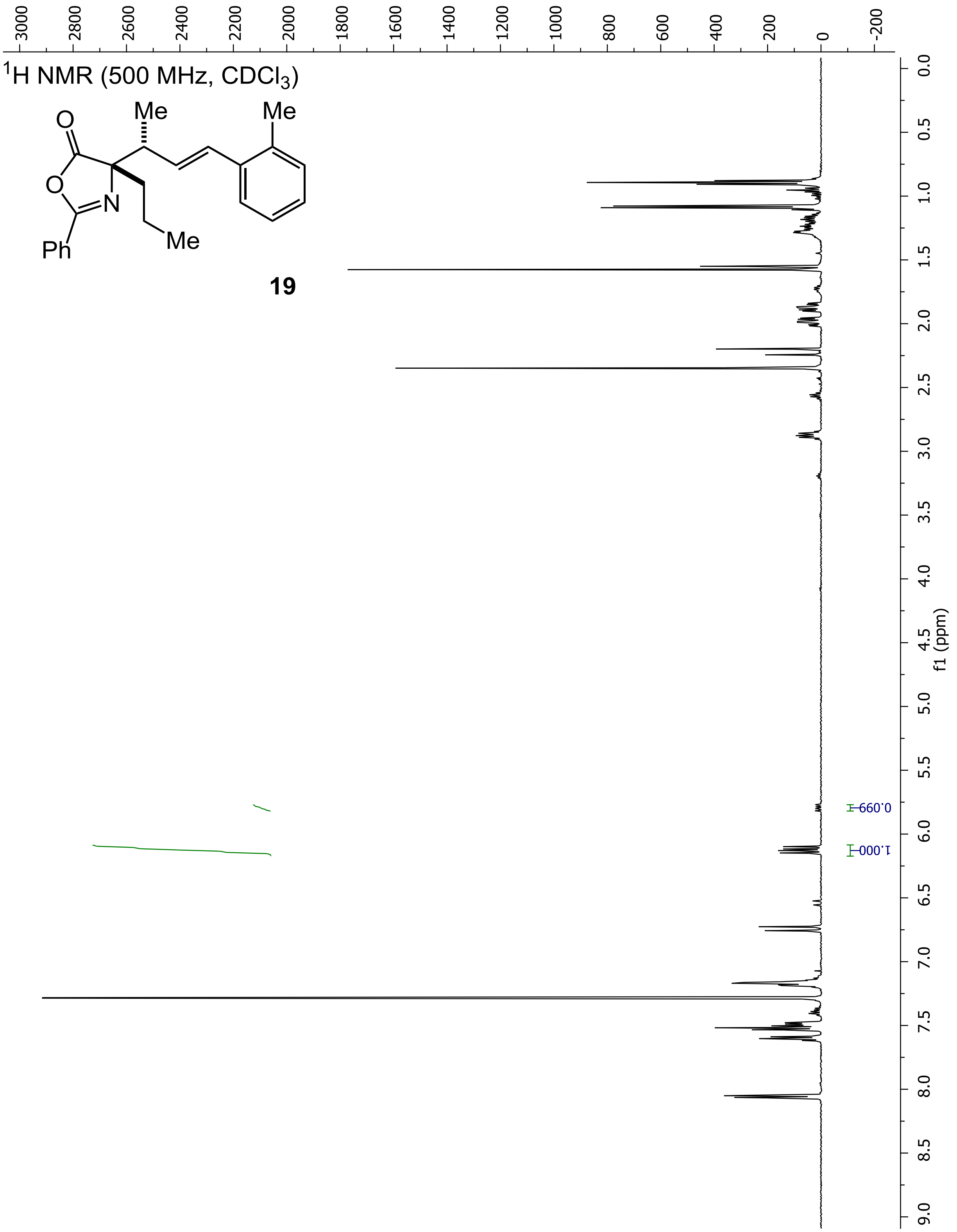

^{1}H NMR (500 MHz, CDCl$_3$)
Me
Me
O
O
N
Ph
Me
19
f1 (ppm)
0.099
1.000

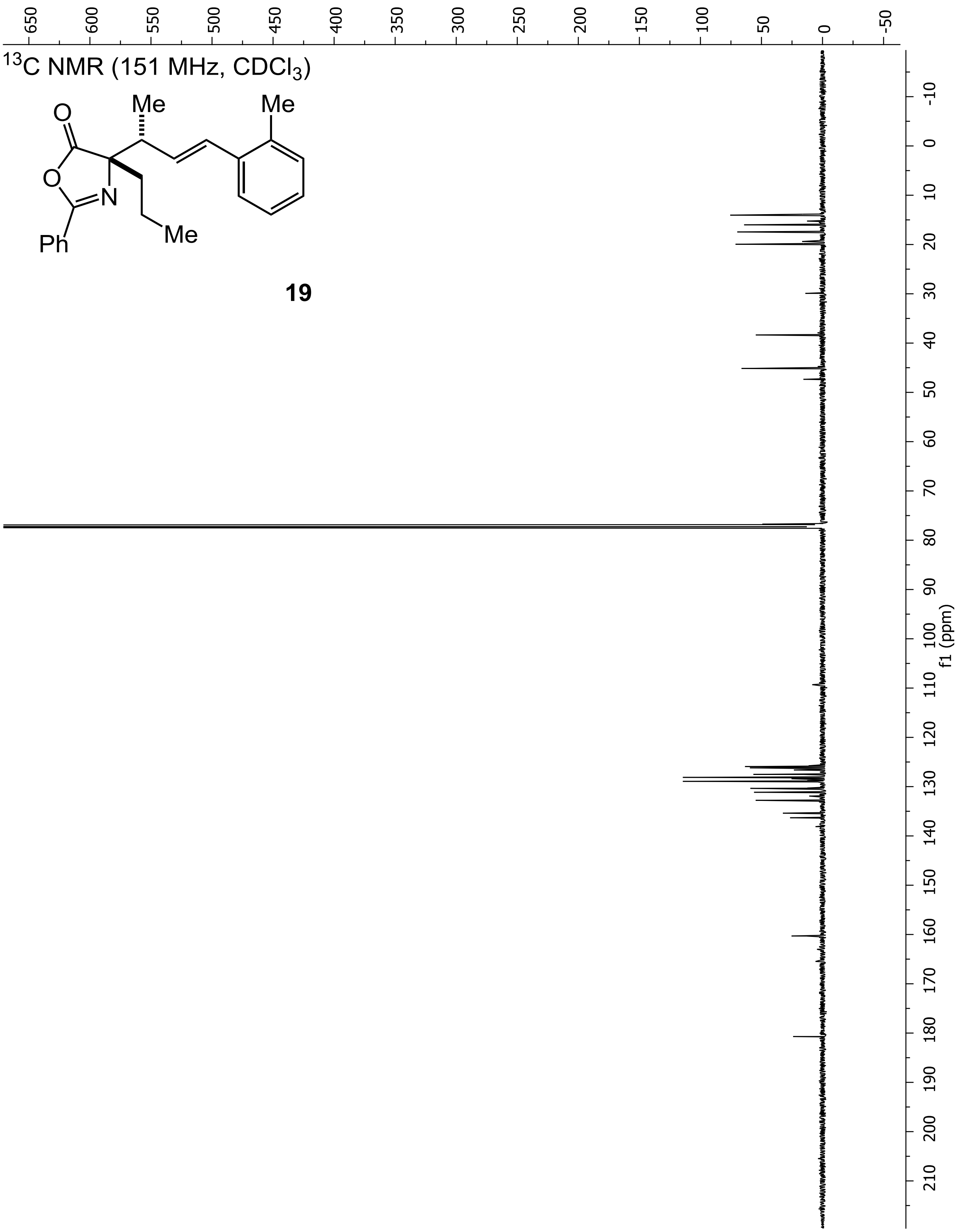

^{13}C NMR (151 MHz, CDCl$_3$)
19
f1 (ppm)

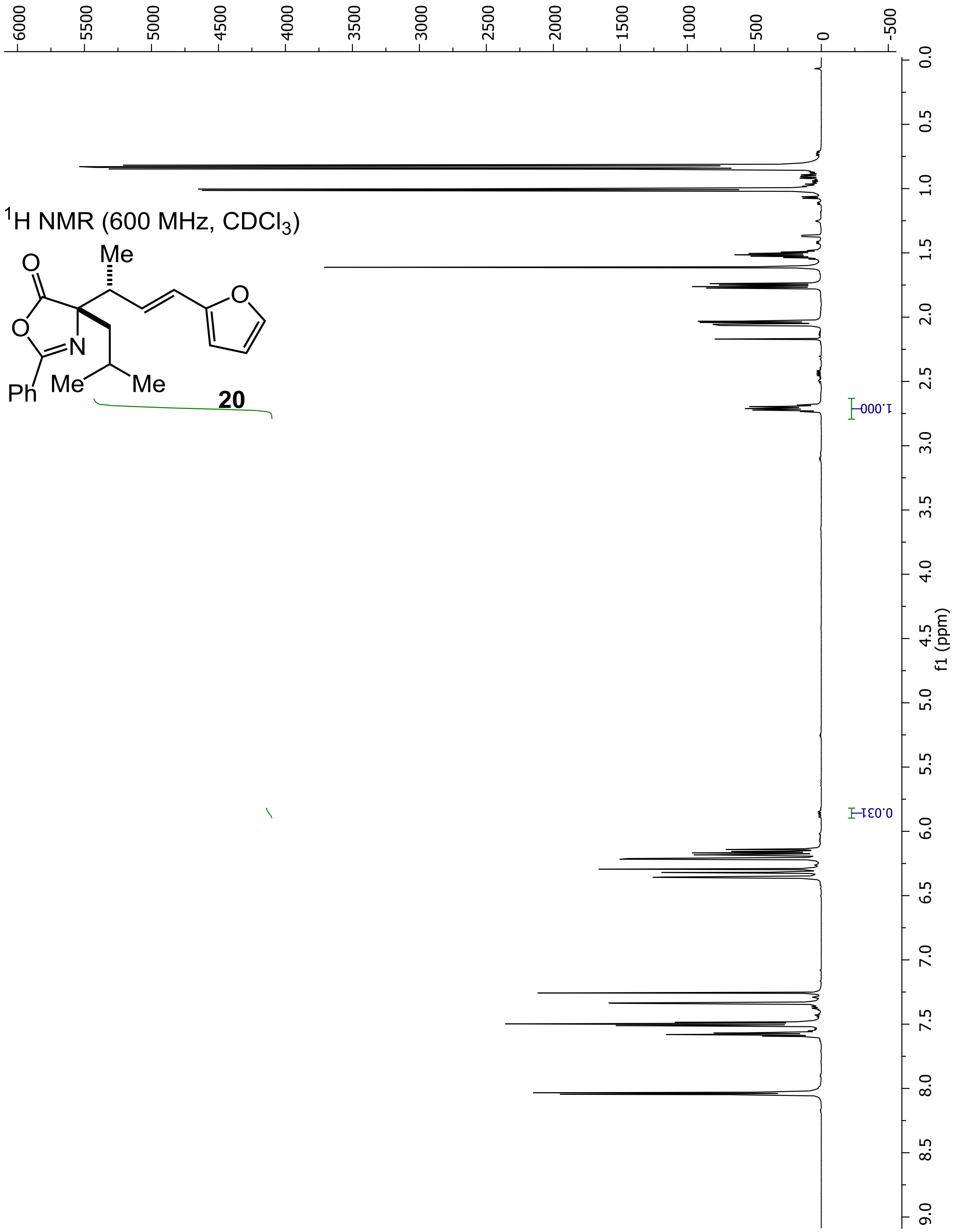

^{1}H NMR (600 MHz, CDCl$_3$)
20
f1 (ppm)
1.000
0.031

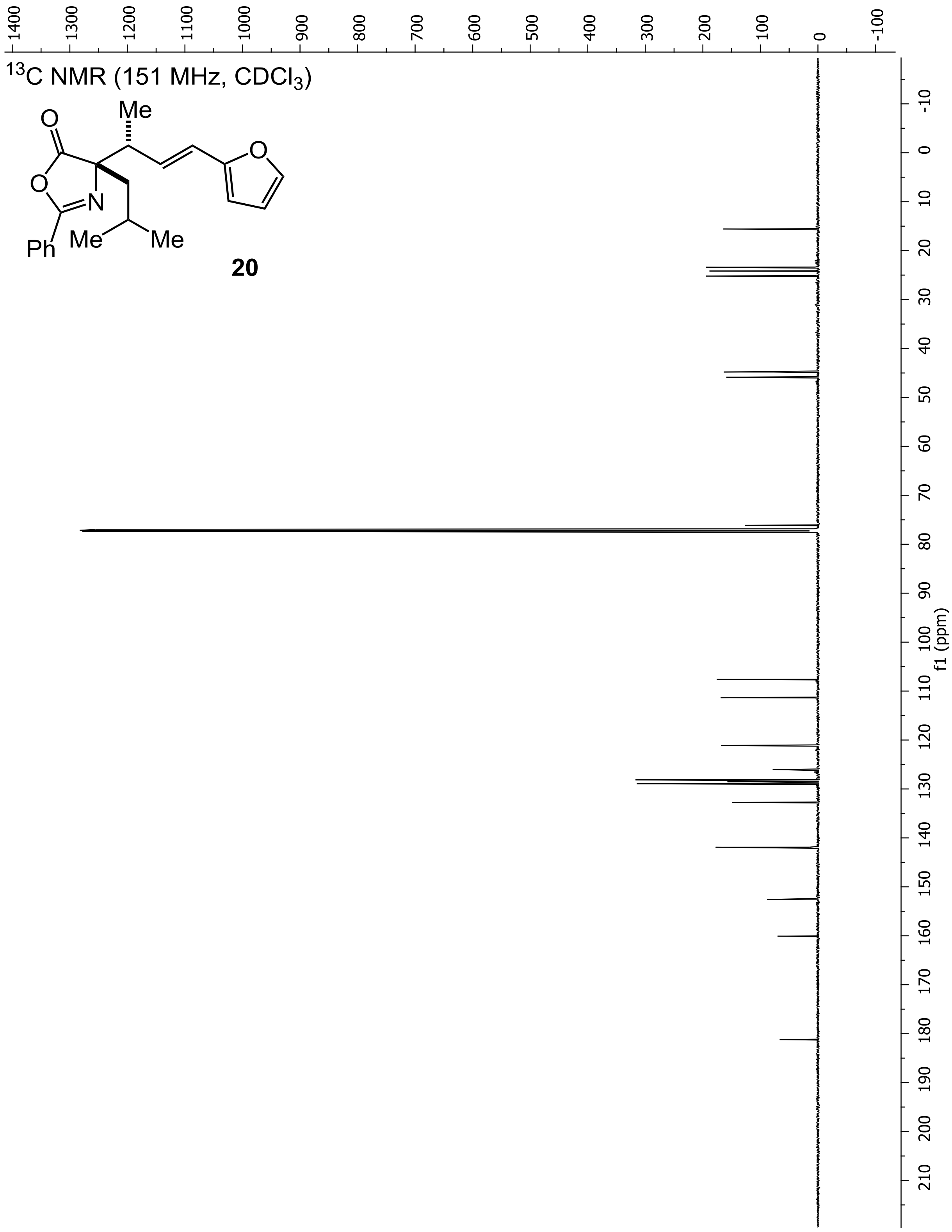

^{13}C NMR (151 MHz, CDCl$_3$)
Me
O
O
N
Ph
Me
Me
20
f1 (ppm)

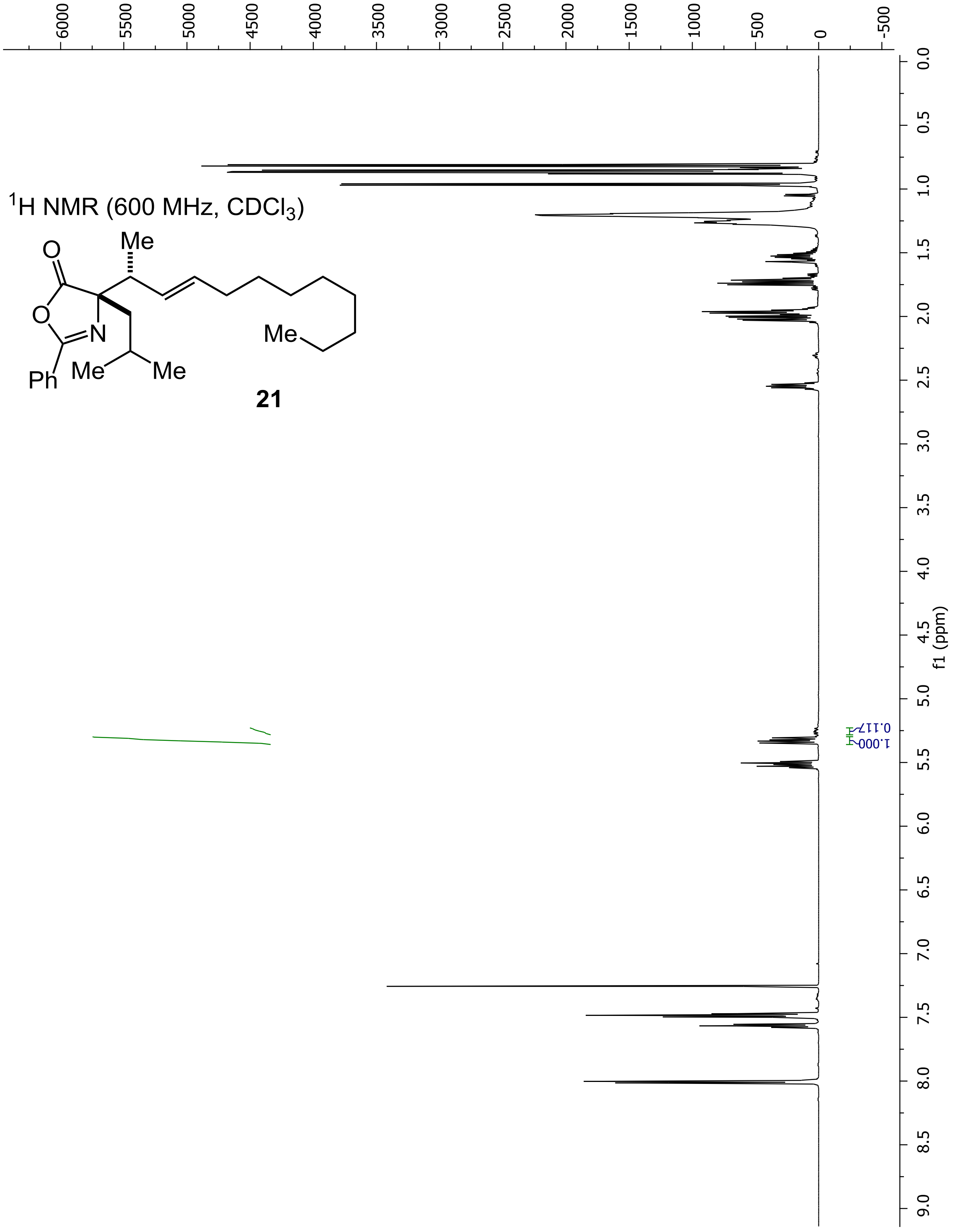

^{1}H NMR (600 MHz, $CDCl_3$)

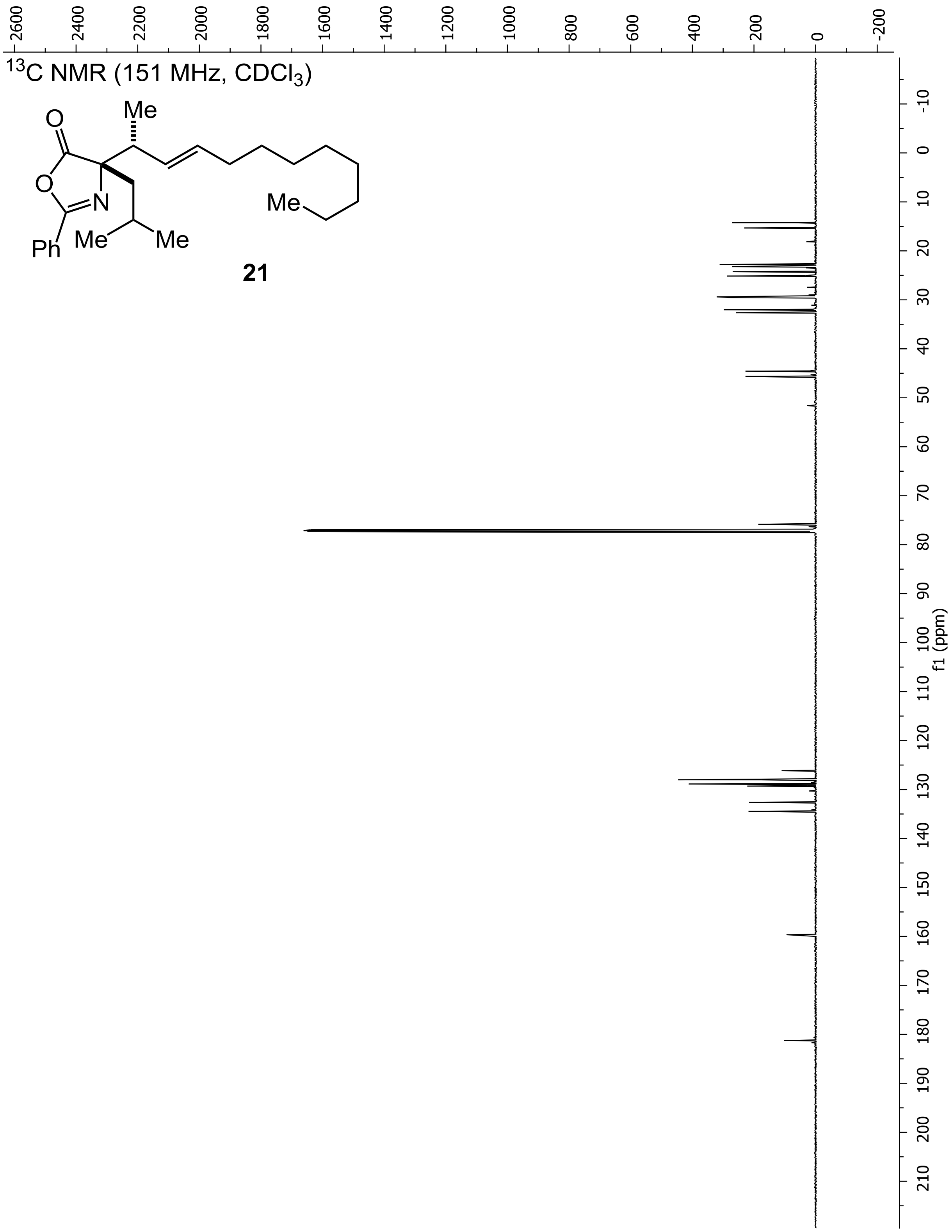

^{13}C NMR (151 MHz, CDCl$_3$)
O
Me
O
N
Me
Ph
Me
Me
21
f1 (ppm)

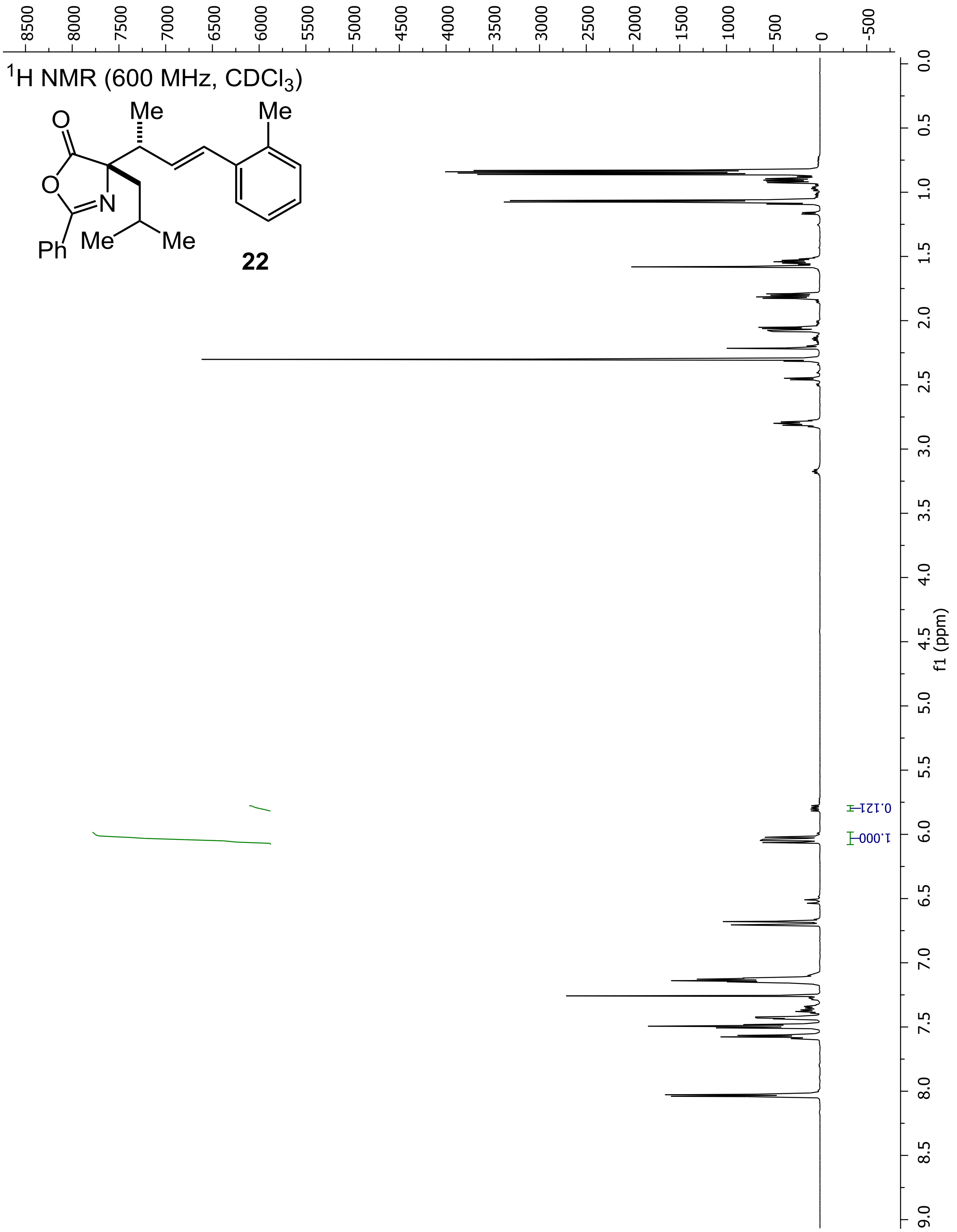
^{1}H NMR (600 MHz, CDCl$_3$)
22
f1 (ppm)
0.121
1.000

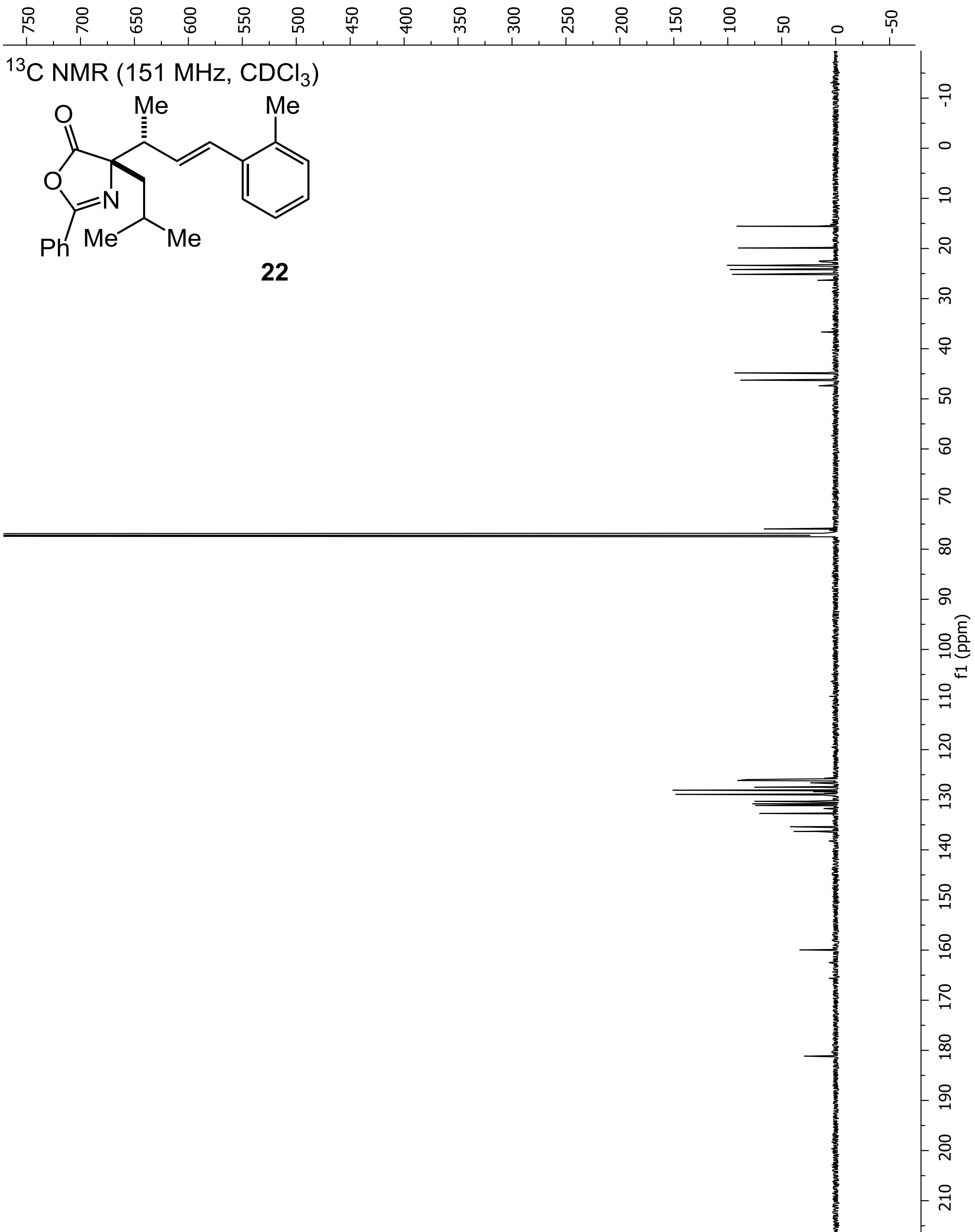
13C NMR (151 MHz, CDCl3)
Me
Me
O
O
N
Ph
Me
Me
22
f1 (ppm)

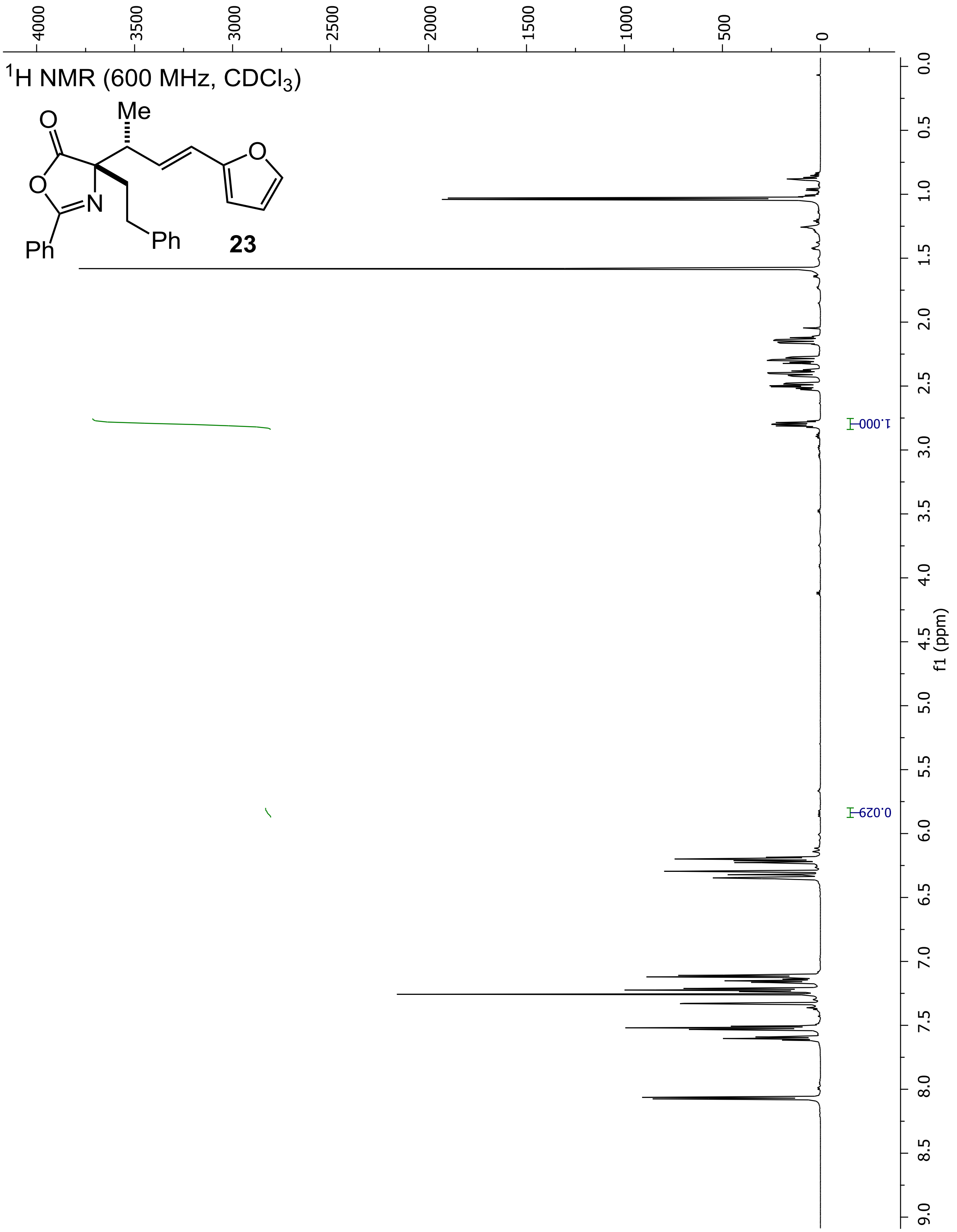

^{1}H NMR (600 MHz, $CDCl_3$)
Me
O
O
N
Ph
Ph
O
23
1.000
0.029
f1 (ppm)

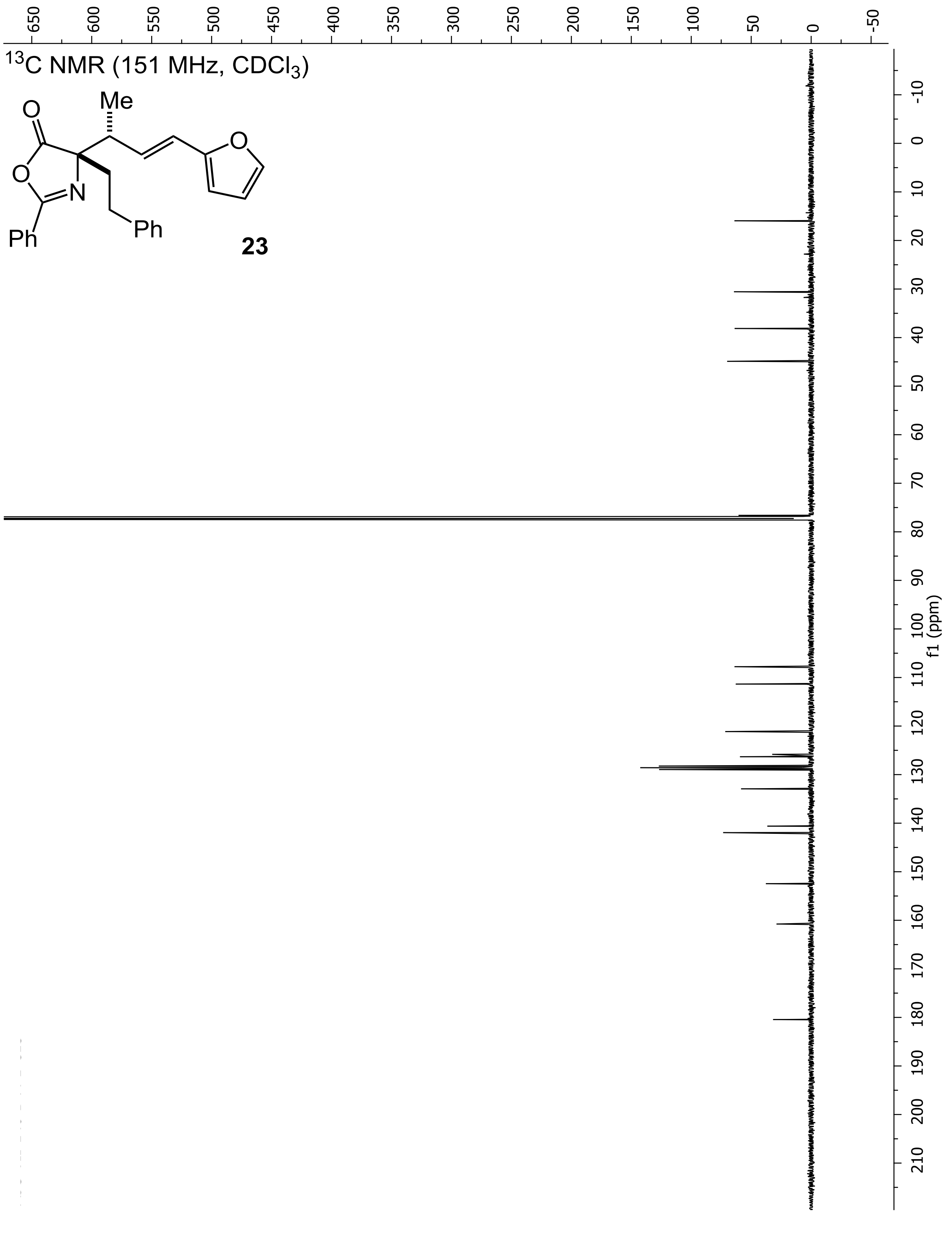

^{13}C NMR (151 MHz, CDCl$_3$)
Me
O
O
N
O
Ph
Ph
23
f1 (ppm)

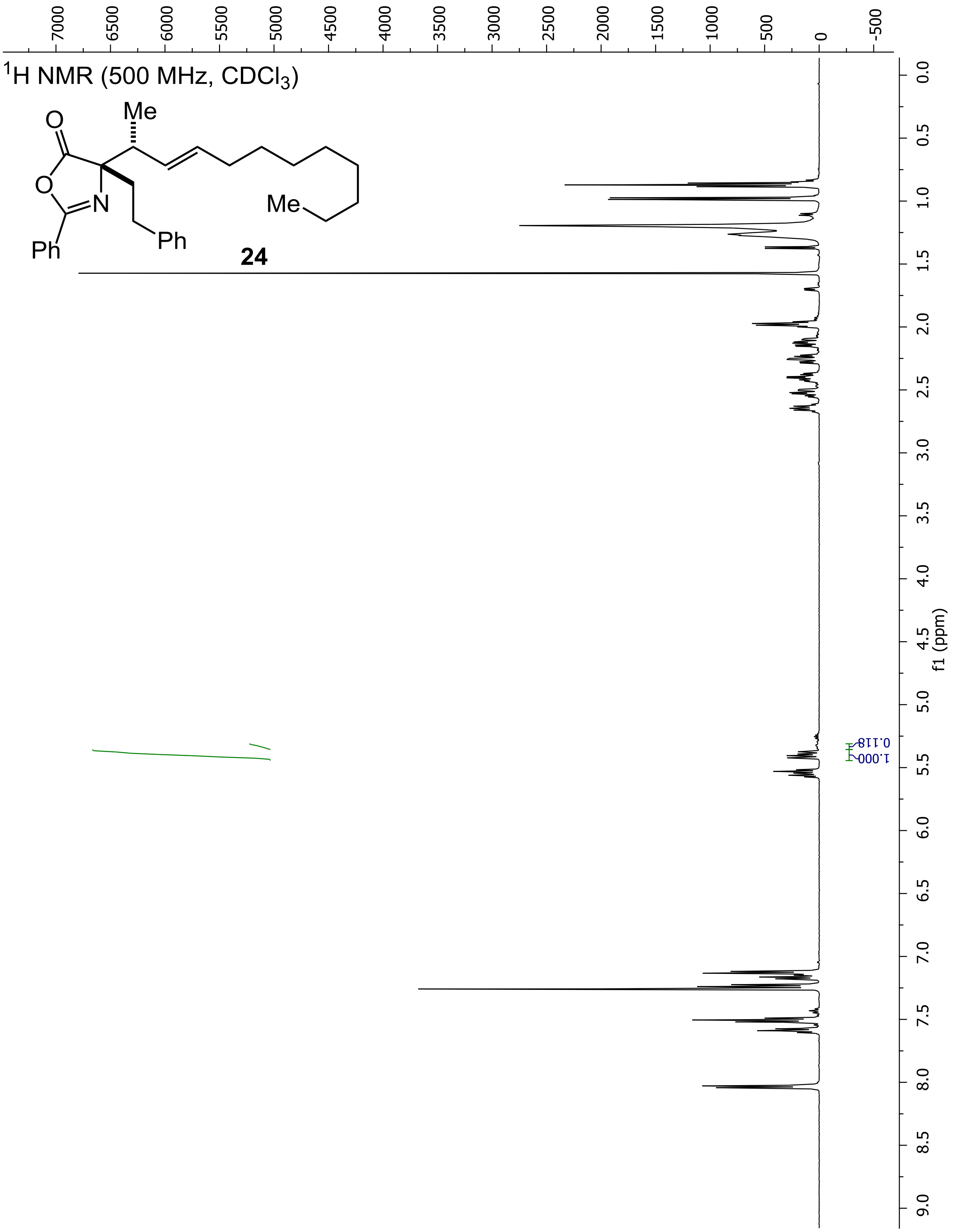

^{1}H NMR (500 MHz, $CDCl_3$)
Me
O
O
N
Ph
Ph
Me
24
f1 (ppm)
0.118
1.000

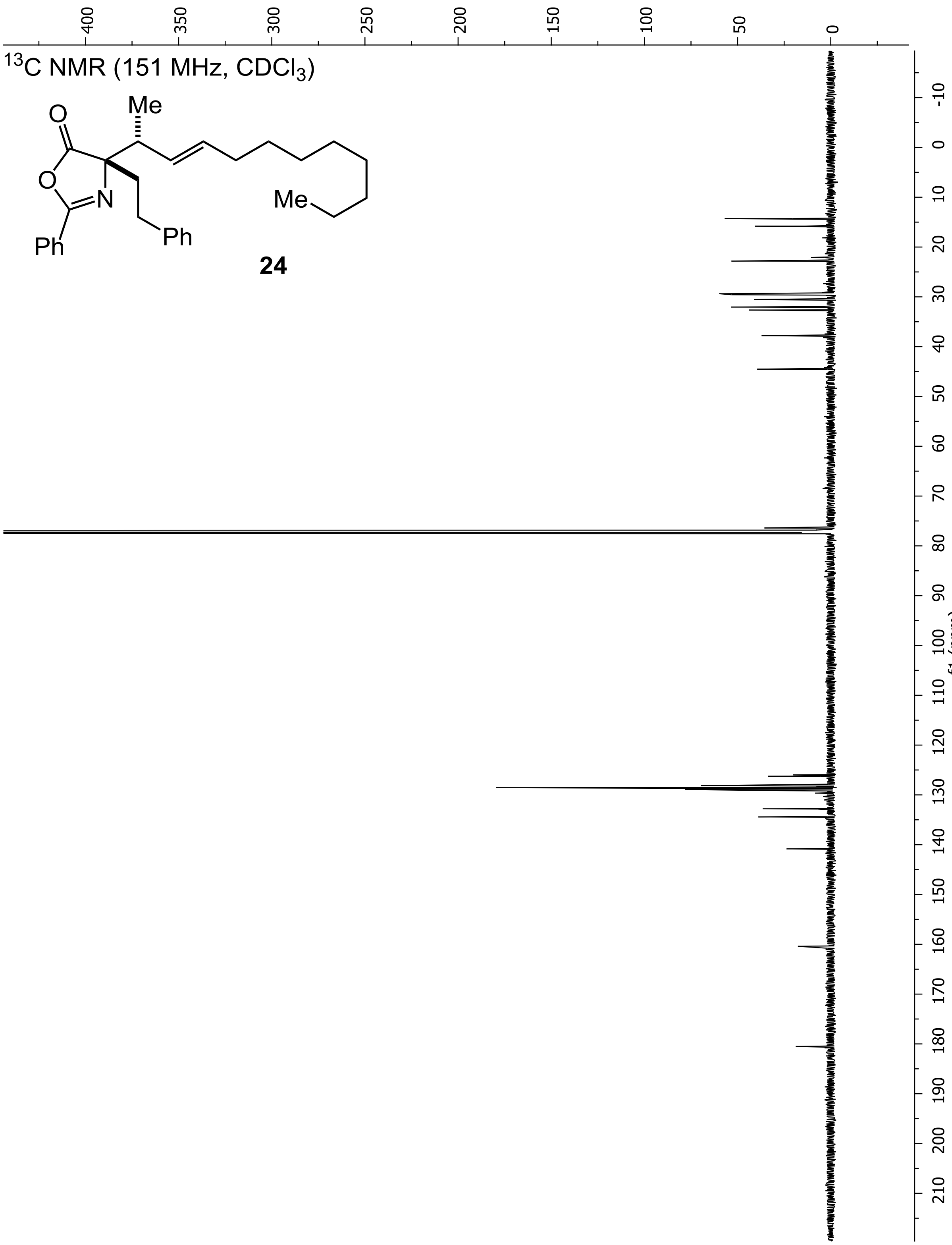
^{13}C NMR (151 MHz, CDCl$_3$)
O
Me
O
N
Me
Ph
Ph
24
f1 (ppm)

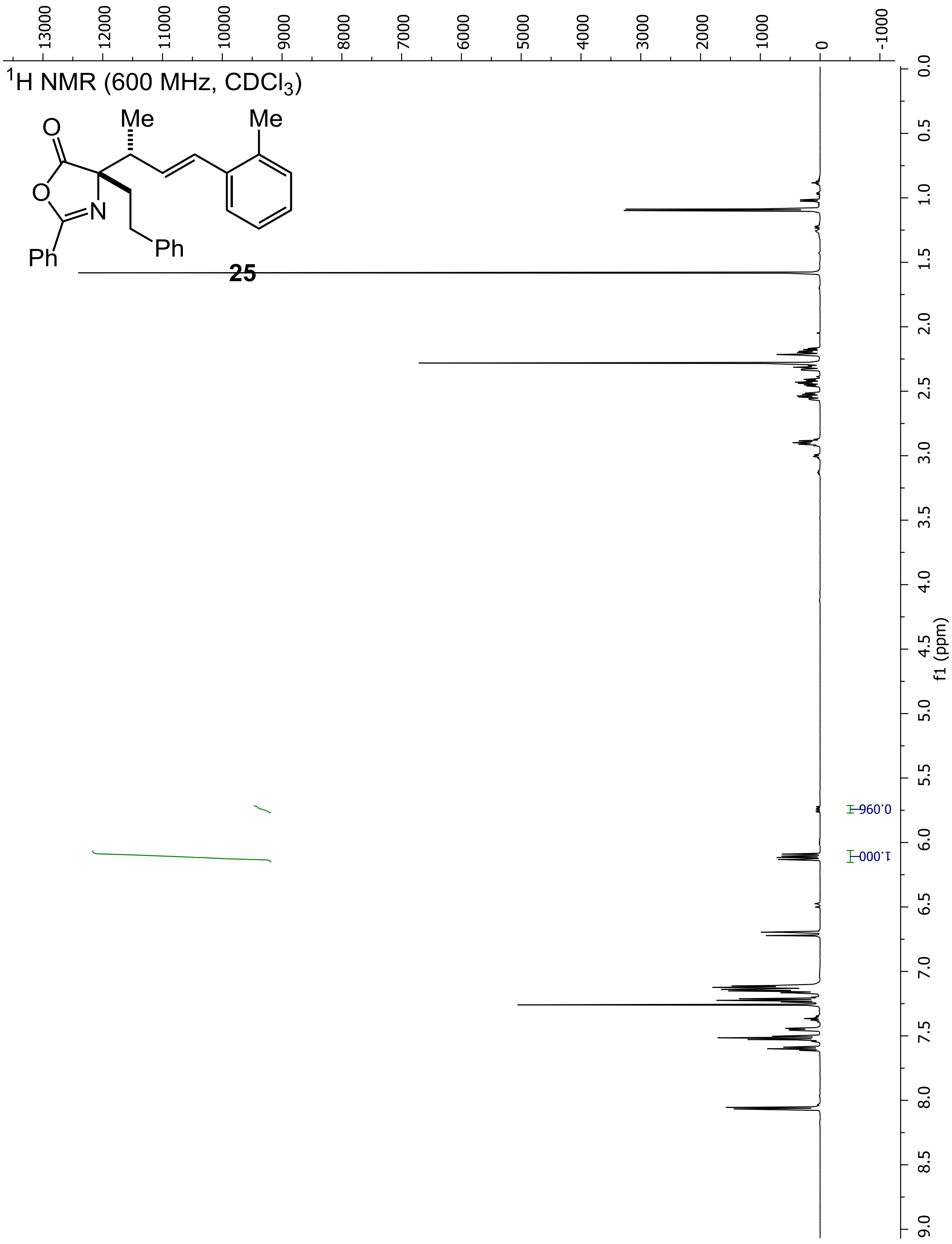

¹H NMR (600 MHz, CDCl₃)
Me
Me
O
O
N
Ph
Ph
25
f1 (ppm)
0.096
1.000

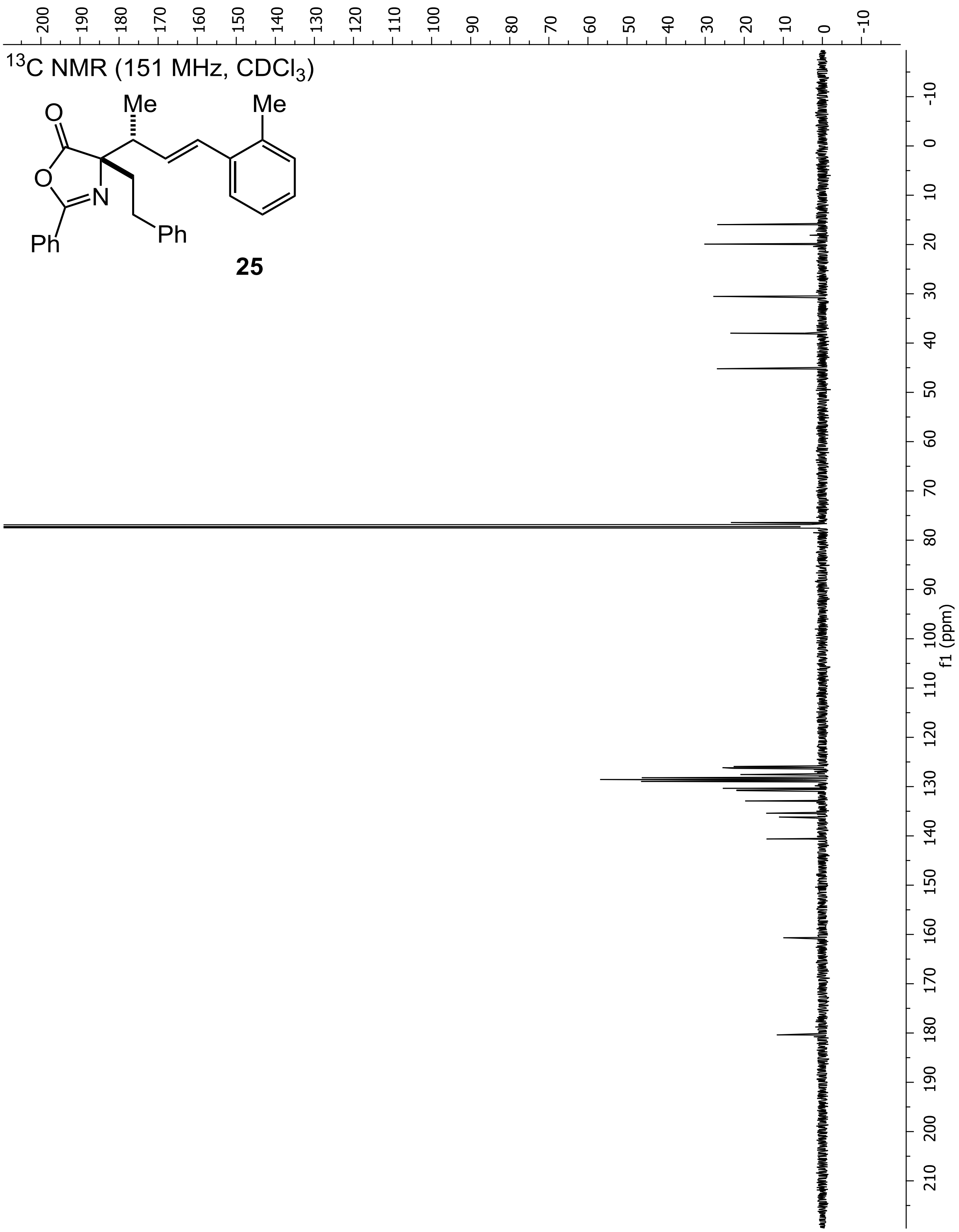

^{13}C NMR (151 MHz, CDCl$_3$)
Me
Me
O
O
N
Ph
Ph
25
f1 (ppm)

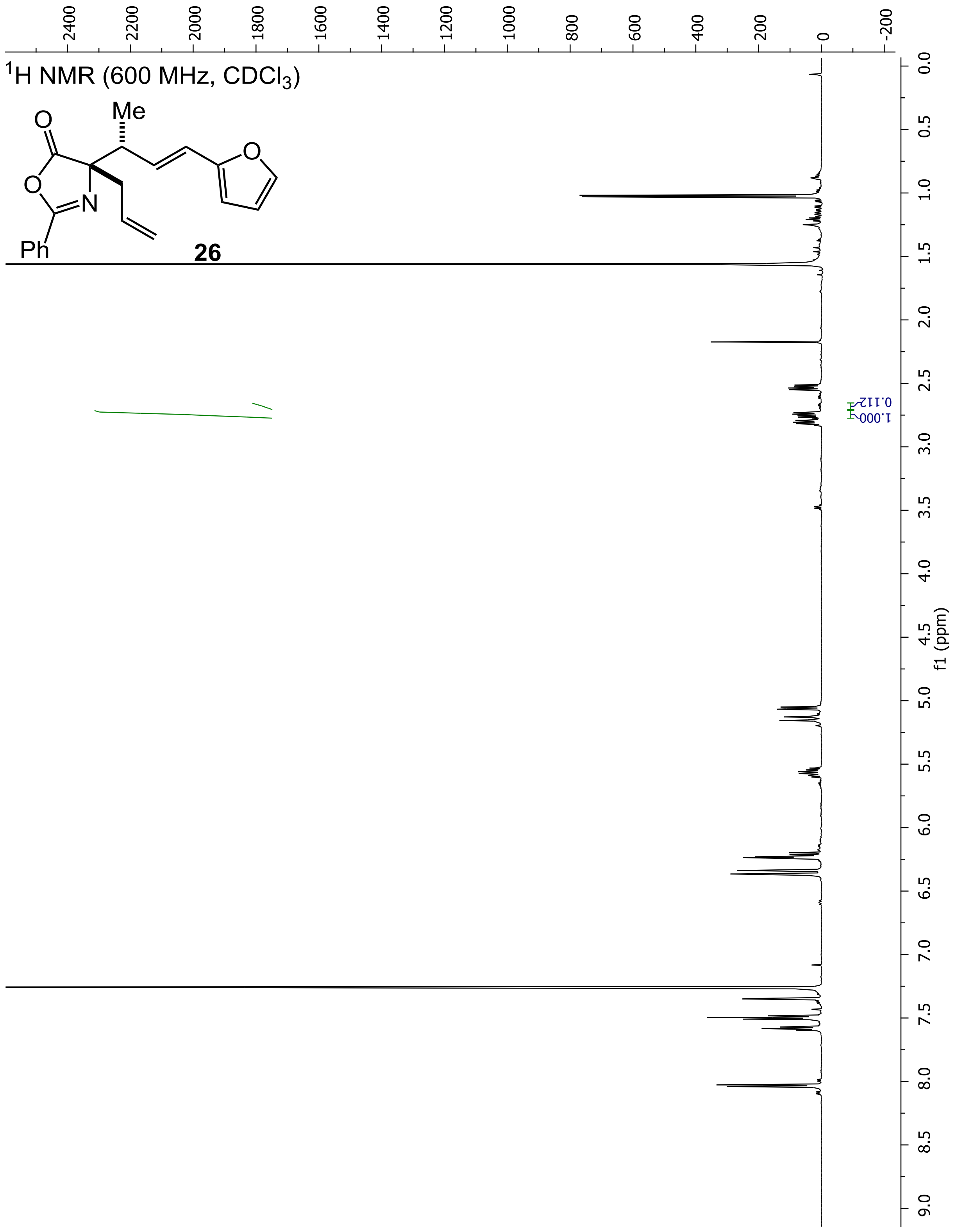

^1H NMR (600 MHz, $CDCl_3$)
Me
O
O
N
Ph
26
0.112
1.000
f1 (ppm)

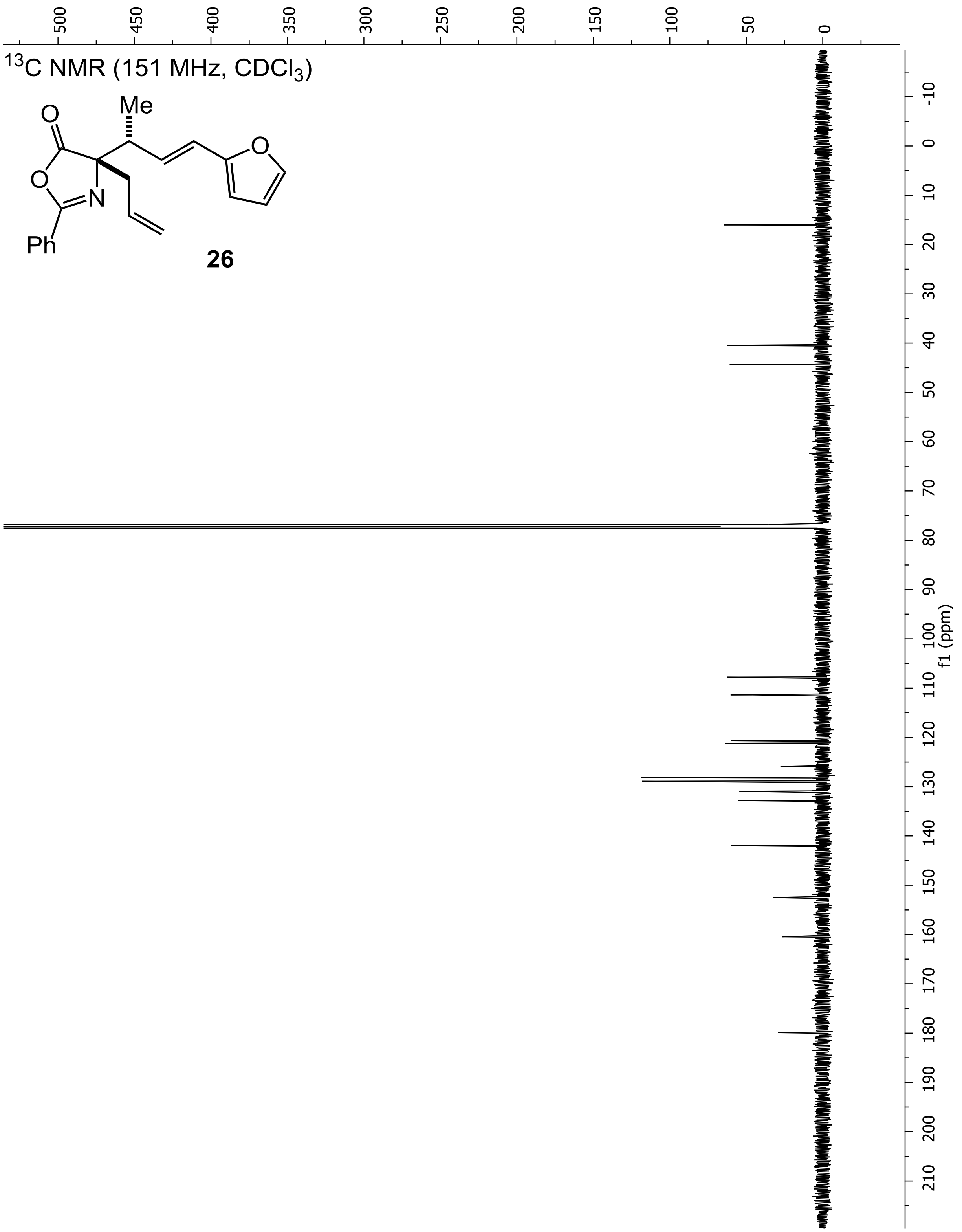
^{13}C NMR (151 MHz, $CDCl_3$)
Me
O
O
N
Ph
26
f1 (ppm)

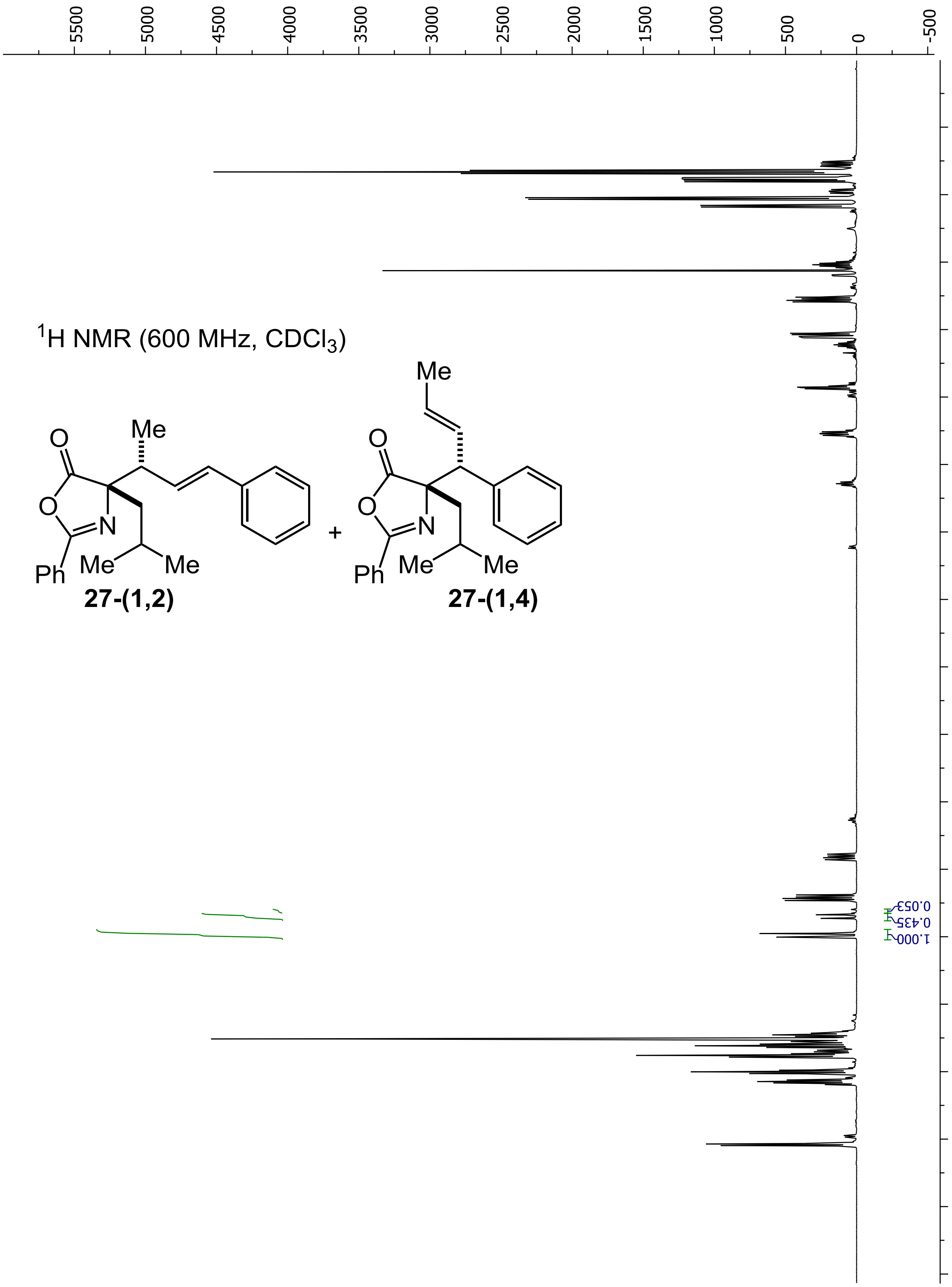

^{1}H NMR (600 MHz, CDCl$_3$)
27-(1,2)
27-(1,4)
f1 (ppm)

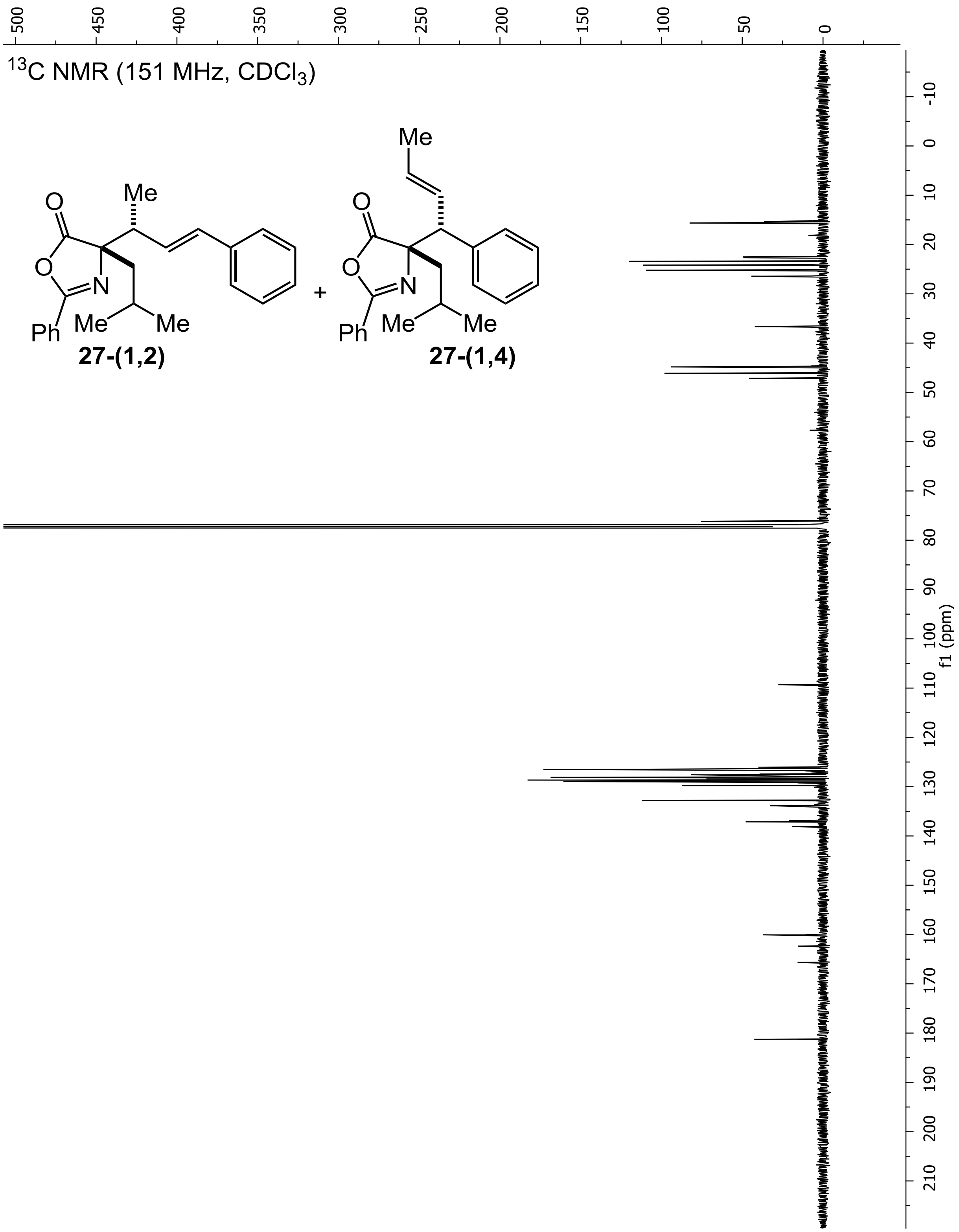

^{13}C NMR (151 MHz, CDCl$_3$)
27-(1,2)
27-(1,4)
f1 (ppm)

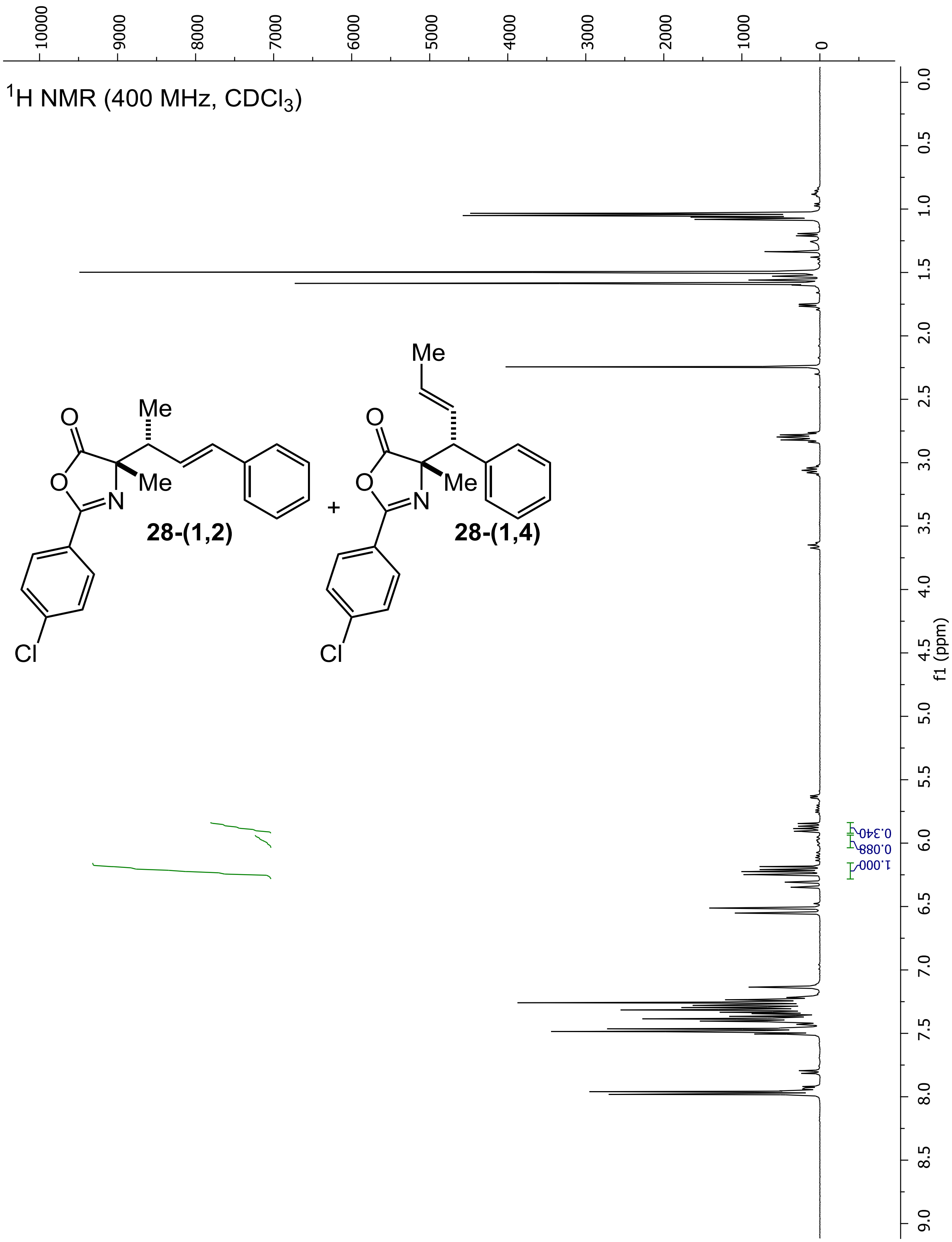

^{1}H NMR (400 MHz, $CDCl_3$)
28-(1,2)
28-(1,4)
f1 (ppm)
0.340
0.088
1.000

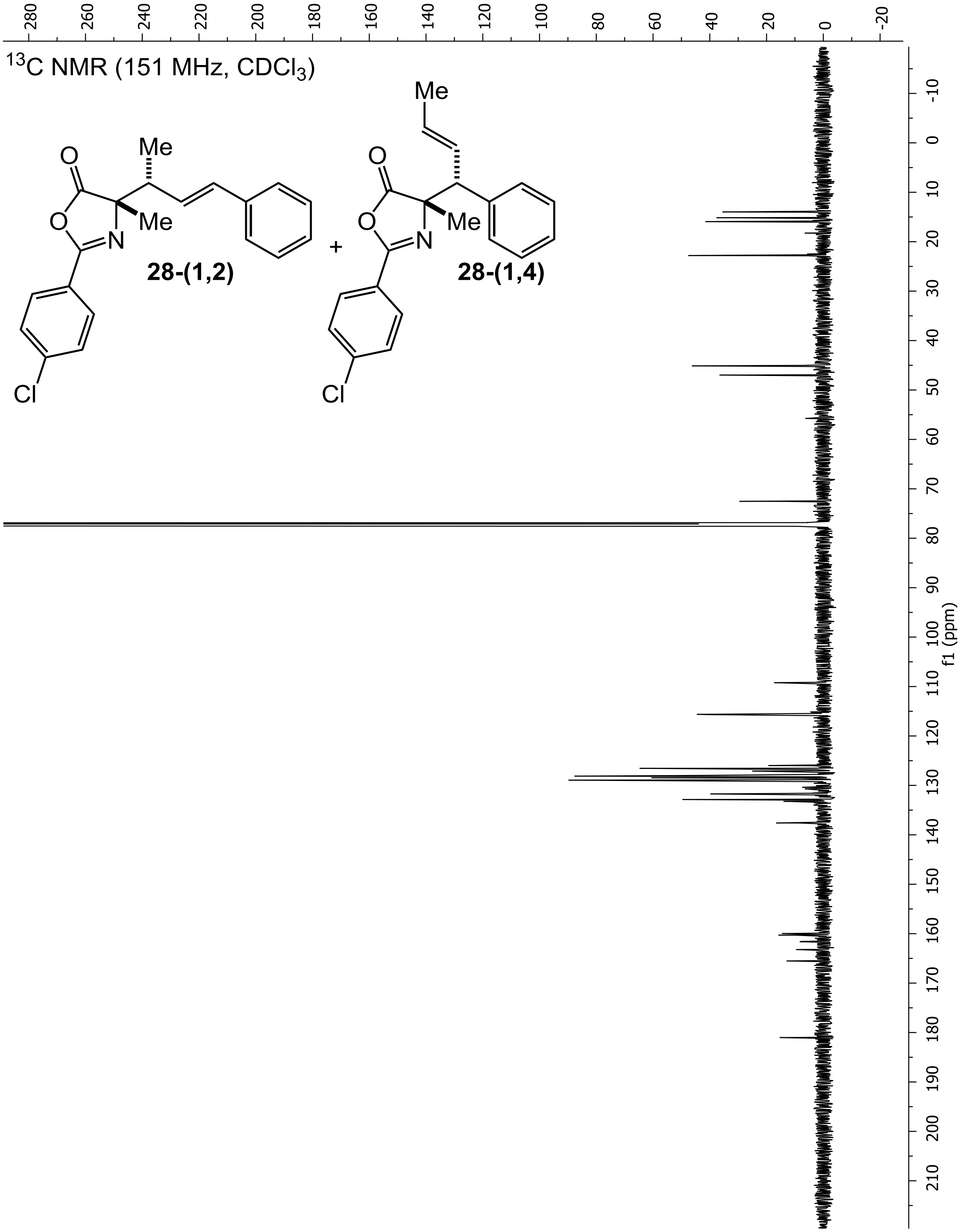

^{13}C NMR (151 MHz, CDCl$_3$)
28-(1,2)
28-(1,4)
f1 (ppm)

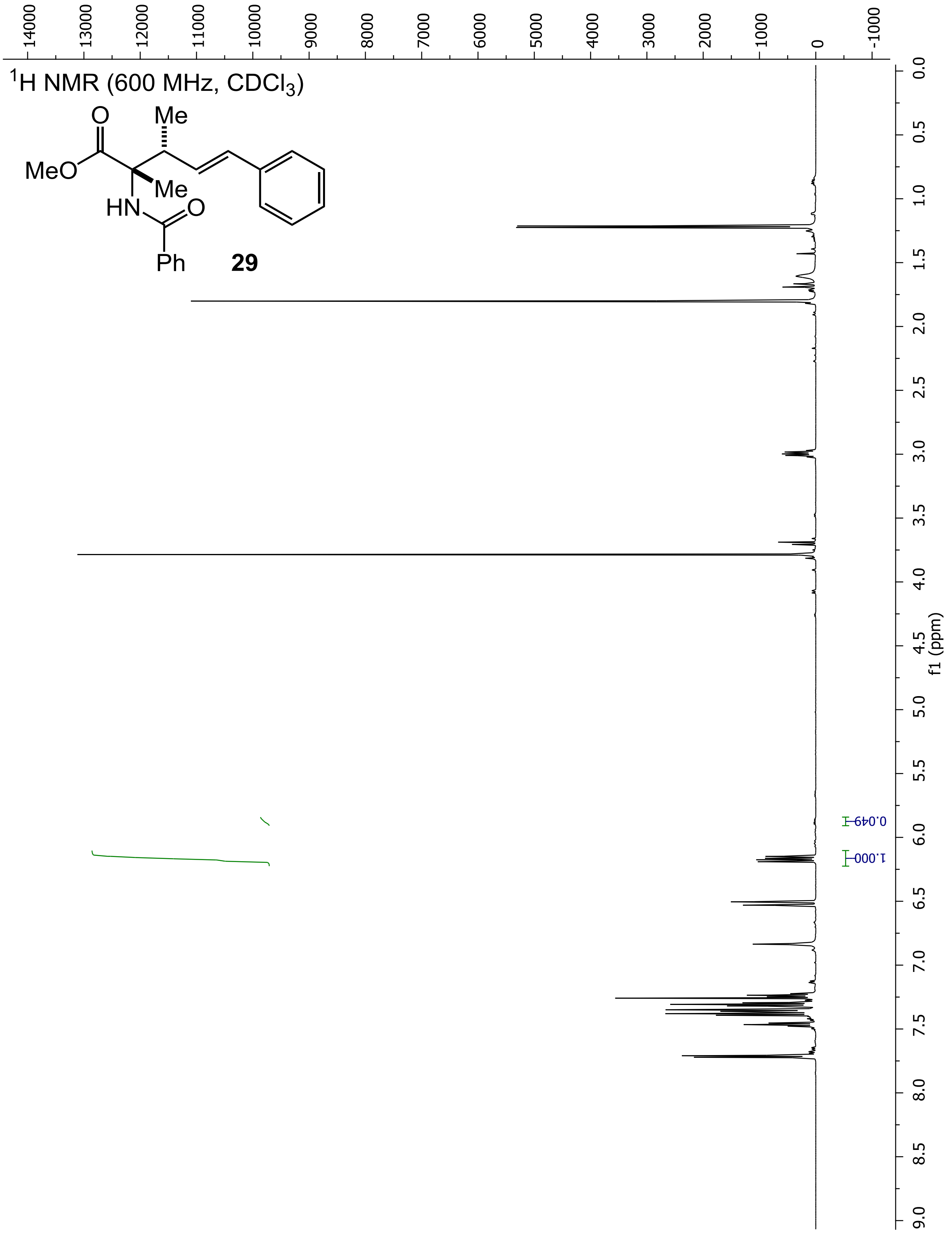

^{1}H NMR (600 MHz, CDCl$_3$)
MeO
O
Me
Me
HN
O
Ph
29
f1 (ppm)
0.049
1.000

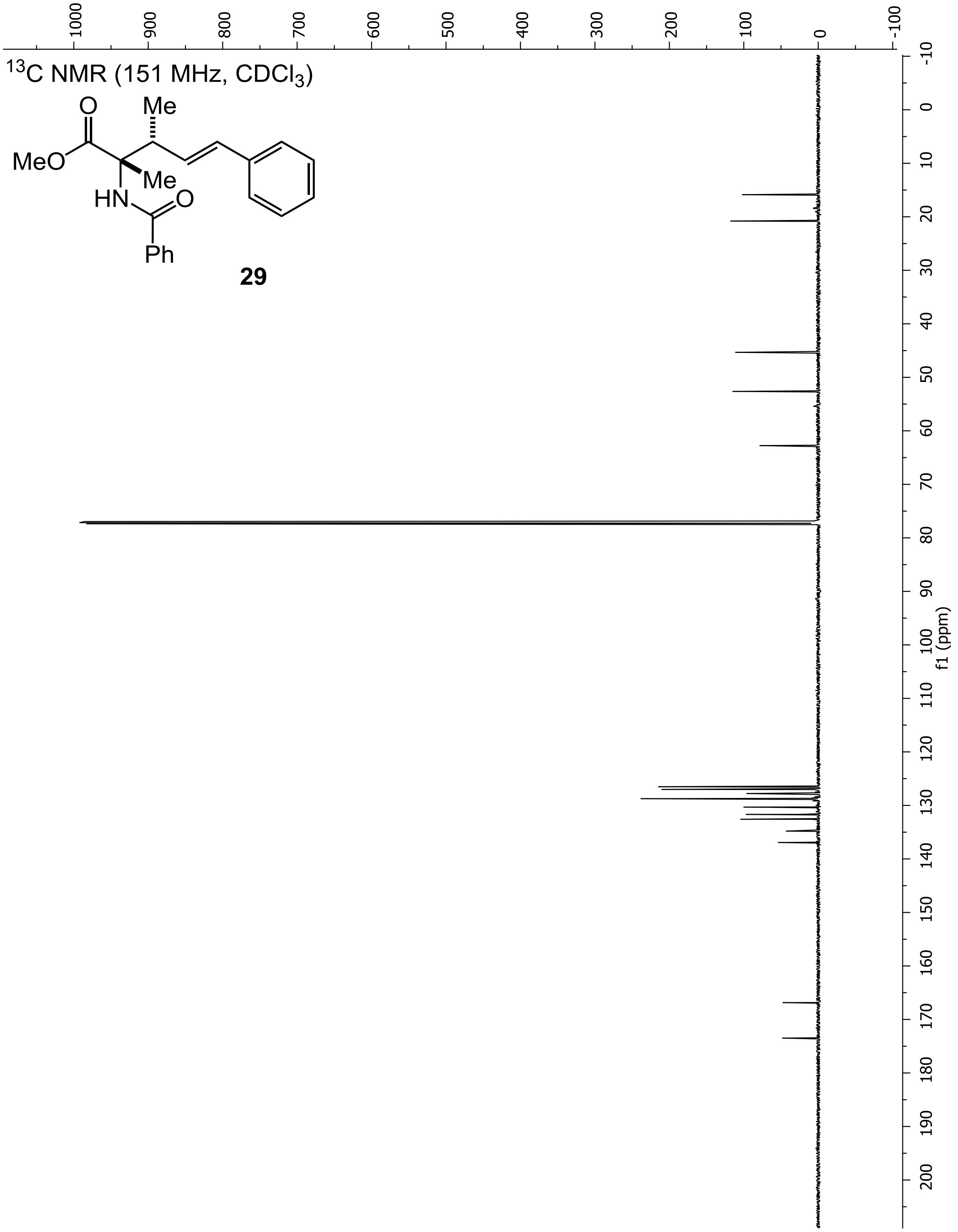

^{13}C NMR (151 MHz, $CDCl_3$)
MeO
O
Me
Me
HN
O
Ph
29
f1 (ppm)

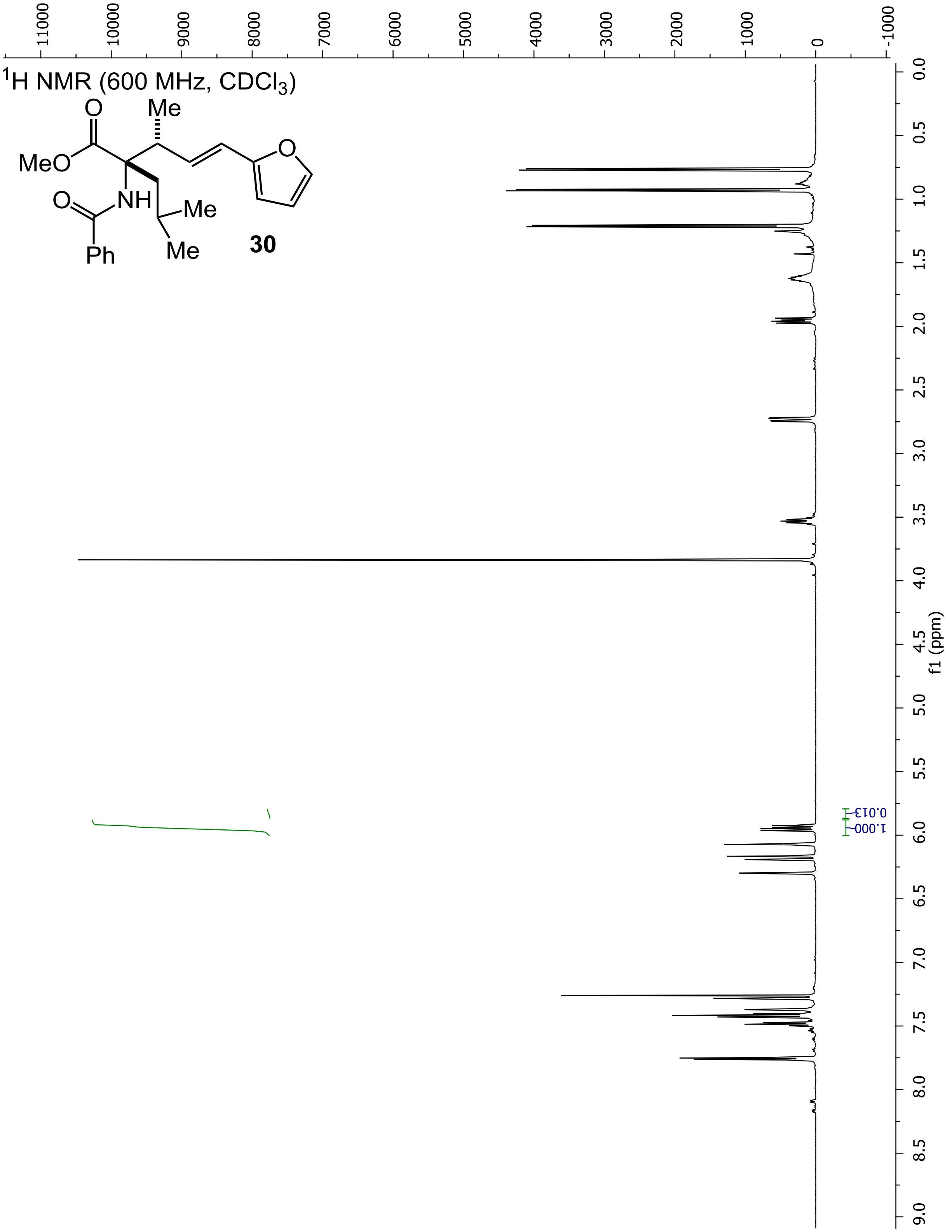
1H NMR (600 MHz, CDCl3)
MeO
O
Me
NH
Me
Me
Ph
30
f1 (ppm)
1.000
0.013

^{13}C NMR (151 MHz, $CDCl_3$)

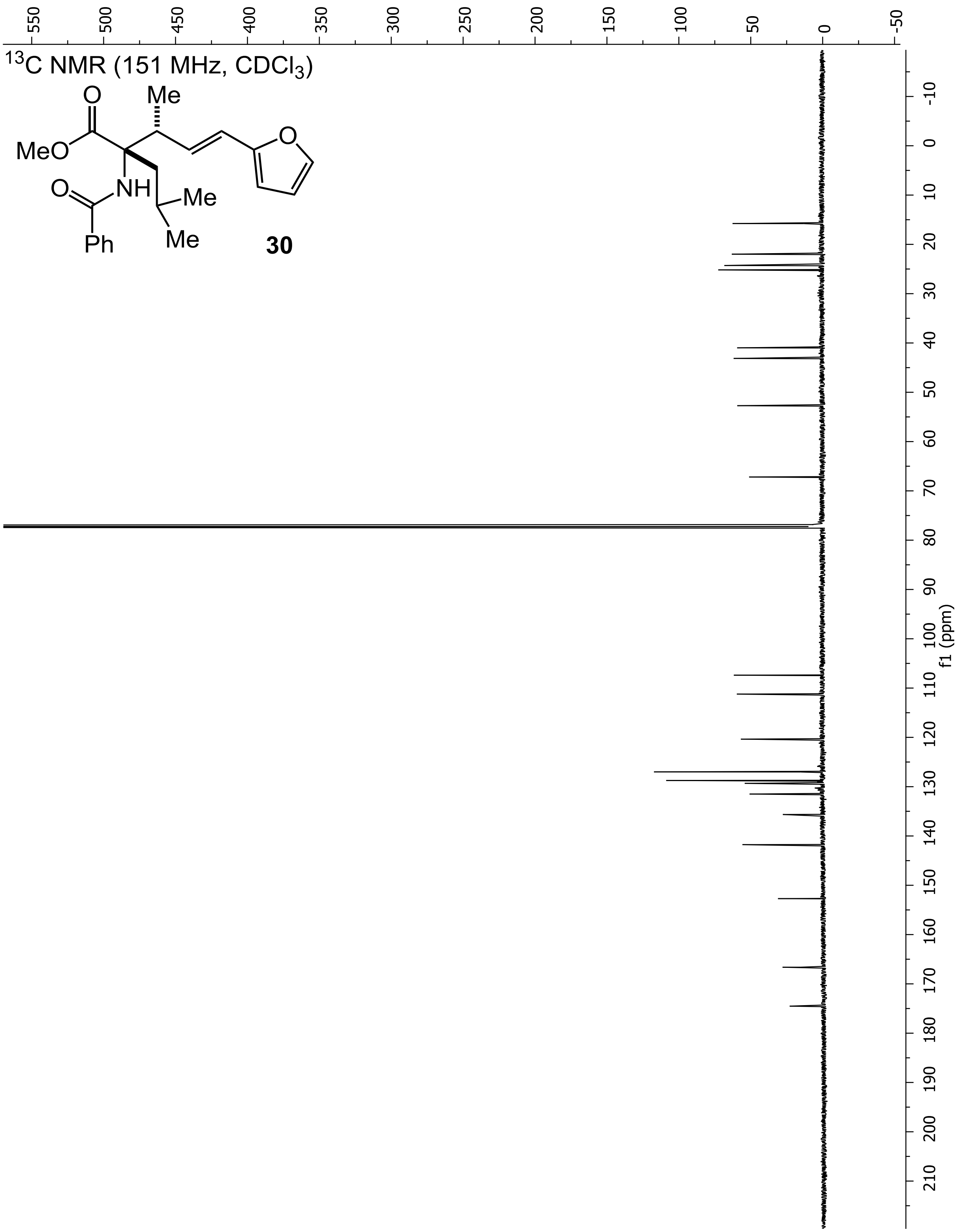

30

^1H NMR (500 MHz, $CDCl_3$)

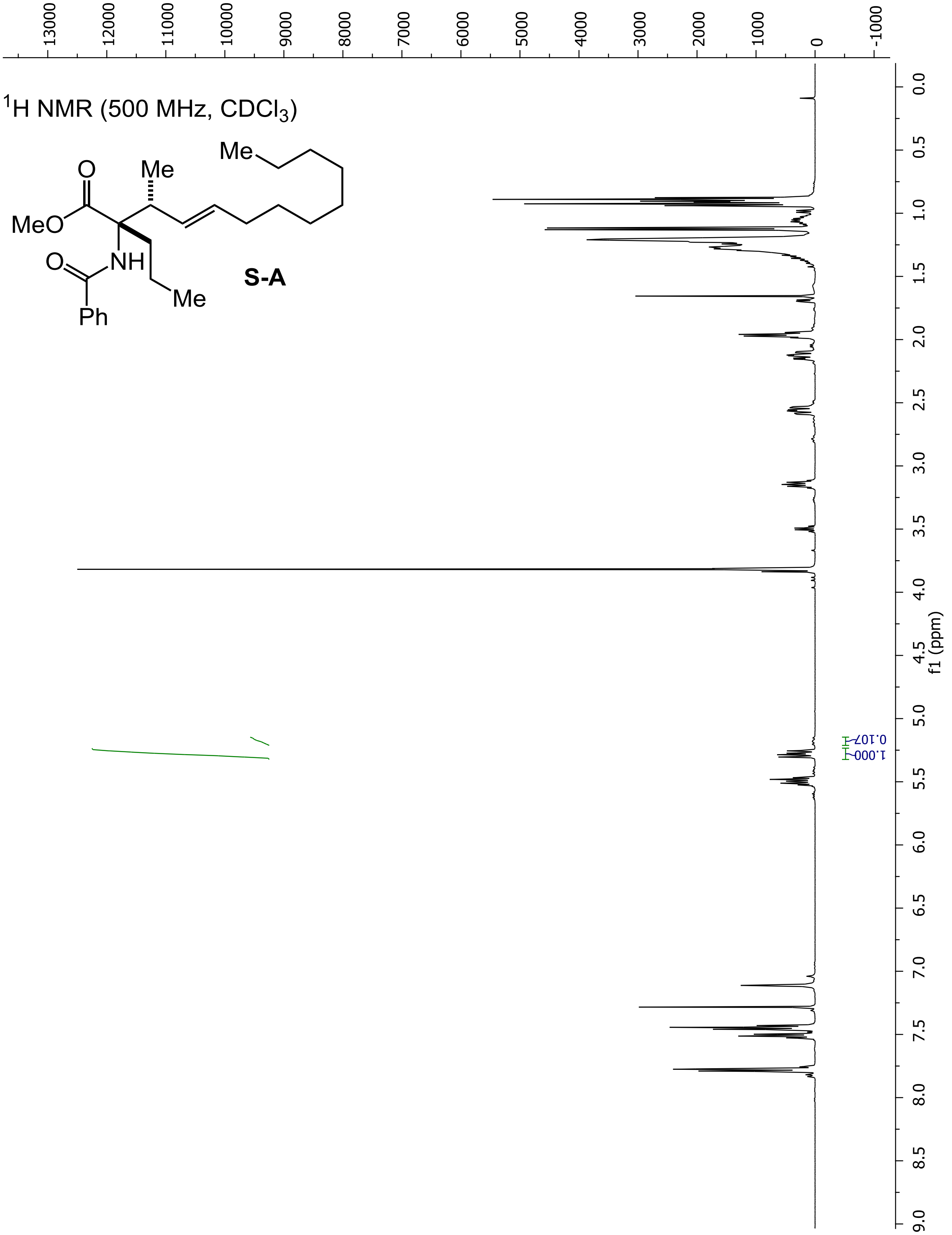

S-A

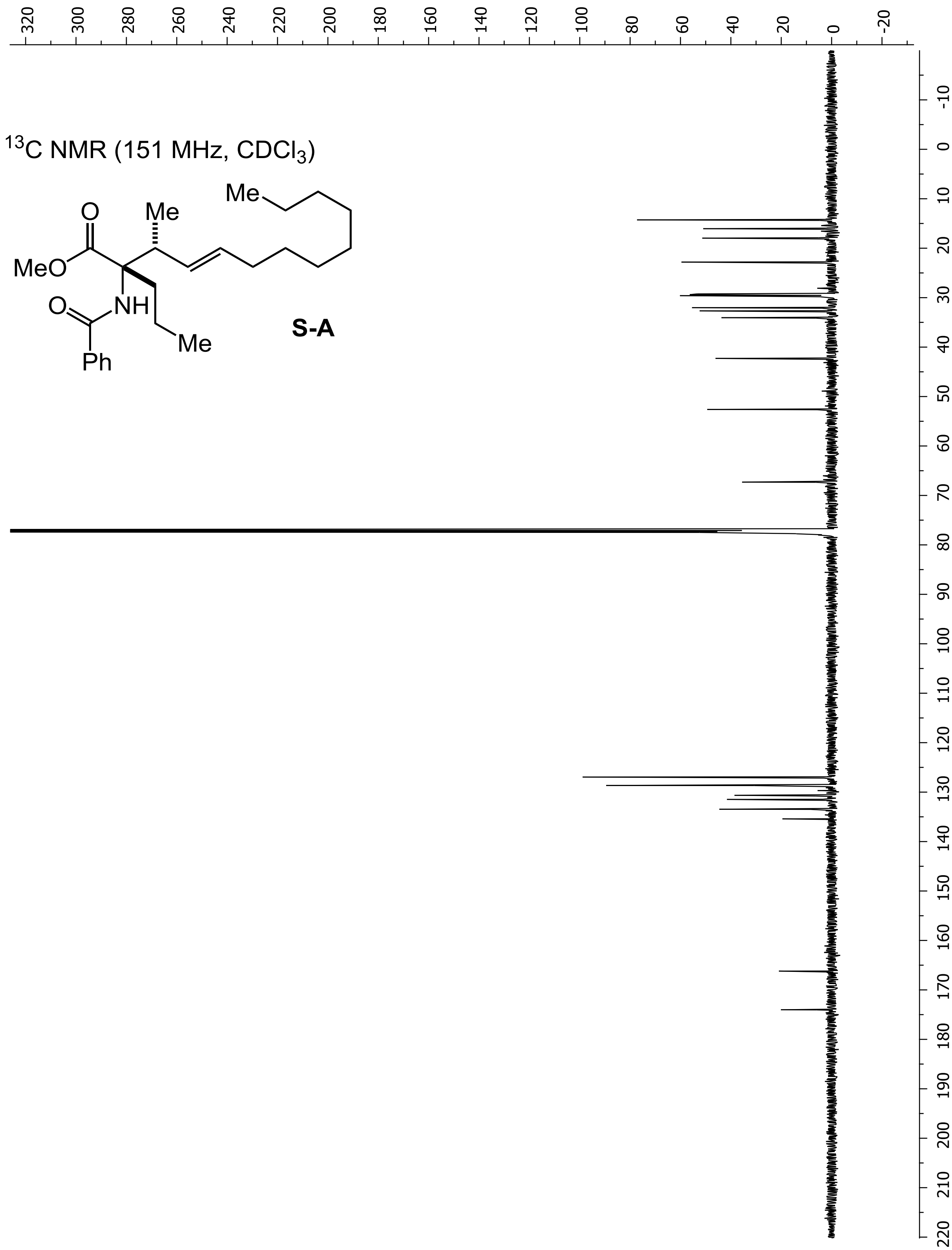

^{13}C NMR (151 MHz, $CDCl_3$)
Me
O
Me
MeO
O
NH
Me
Ph
S-A
f1 (ppm)

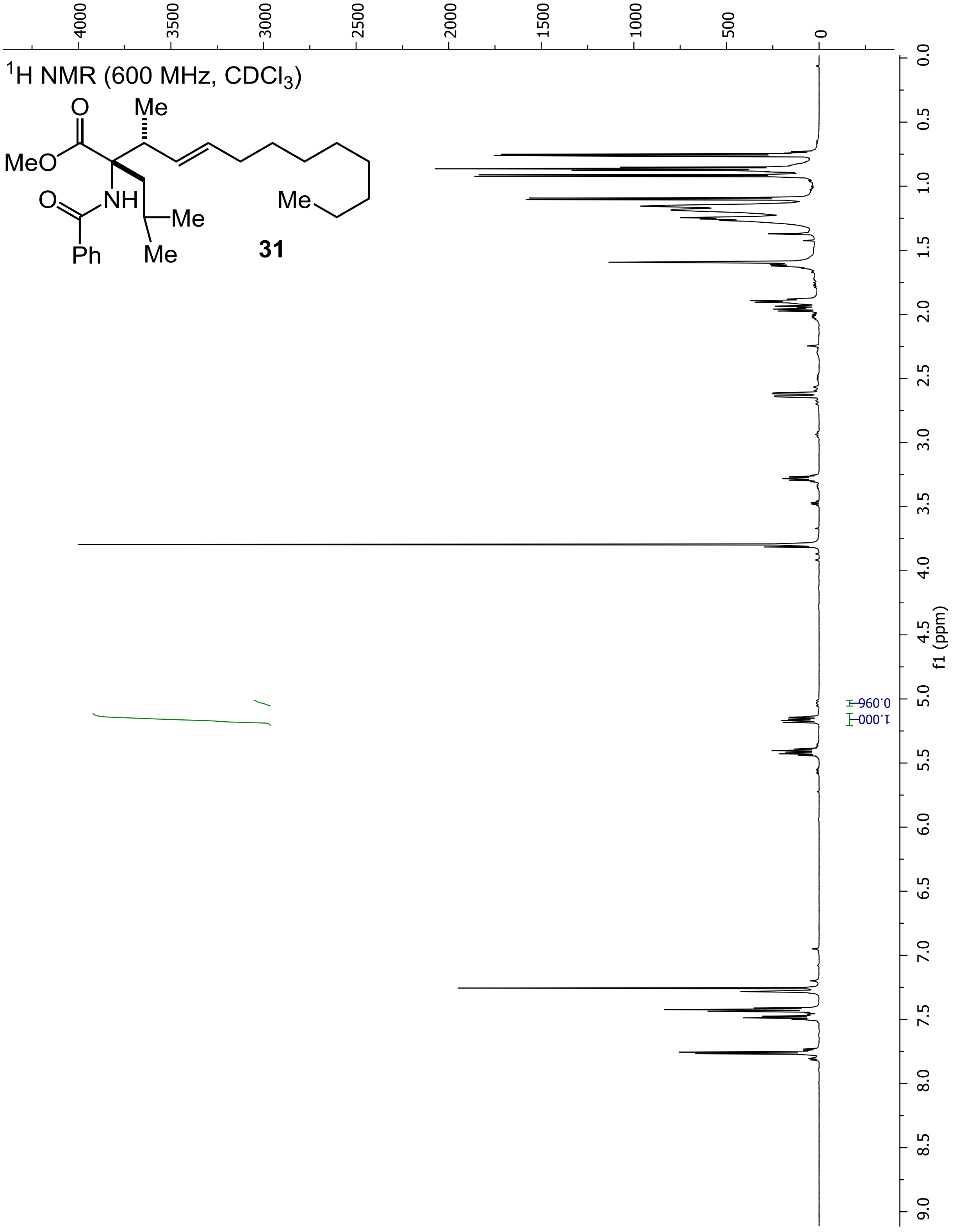

^{1}H NMR (600 MHz, CDCl$_3$)
MeO
O
Me
NH
O
Ph
Me
Me
Me
31
f1 (ppm)
0.096
1.000

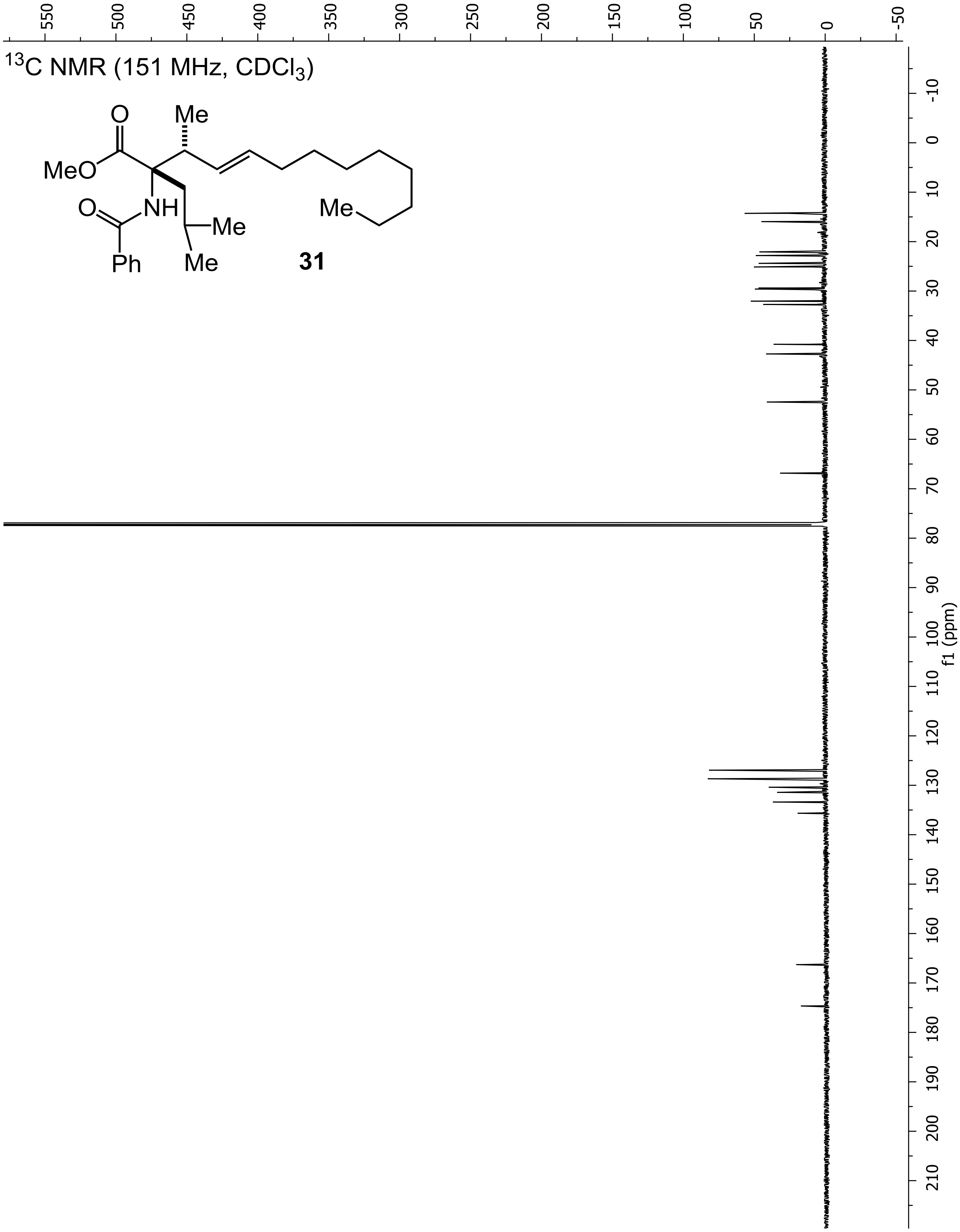

^{13}C NMR (151 MHz, CDCl$_3$)
MeO
O
NH
Me
Ph
Me
Me
Me
31
f1 (ppm)

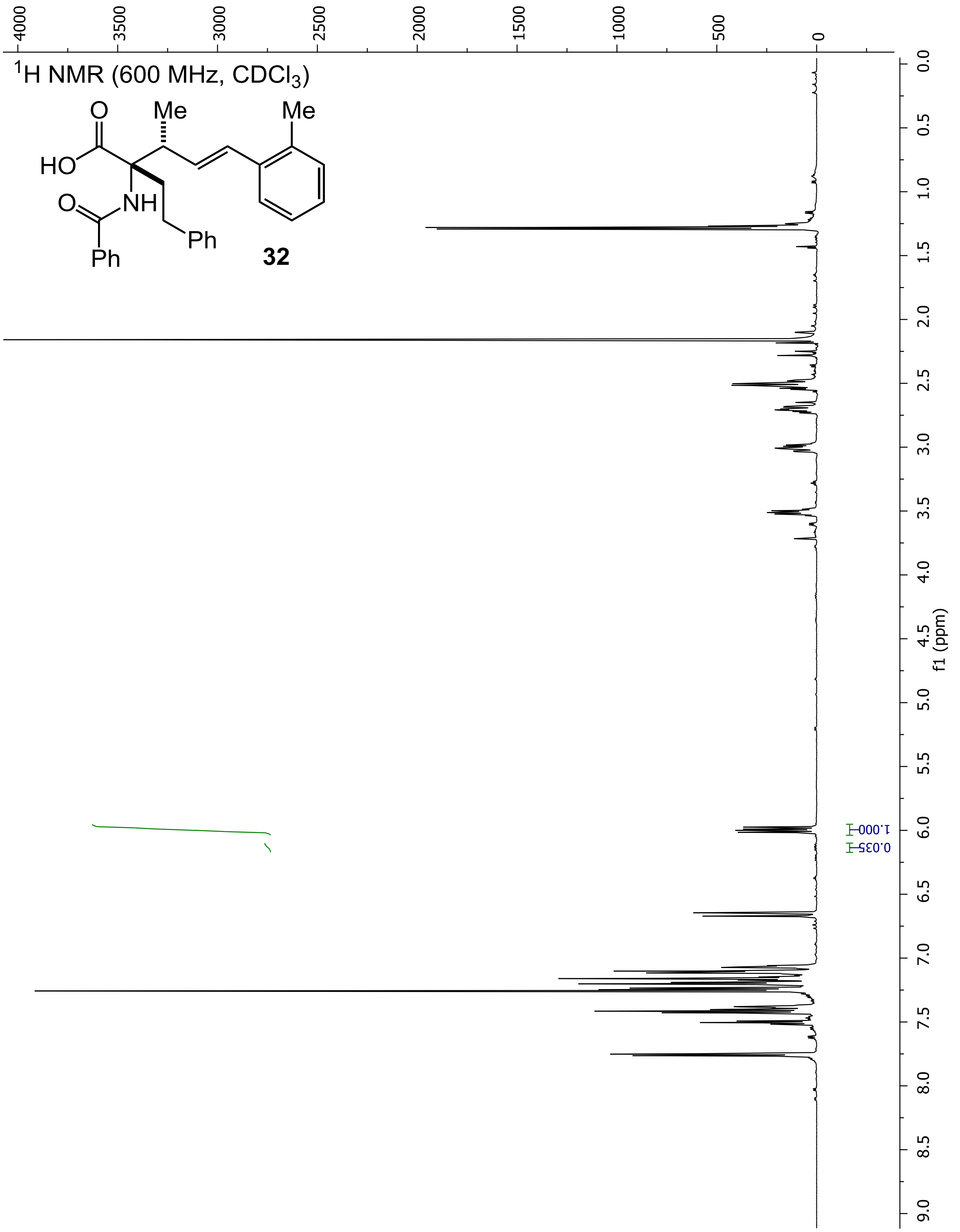

1H NMR (600 MHz, CDCl3)
HO
O
Me
Me
NH
O
Ph
Ph
32
f1 (ppm)
1.000
0.035

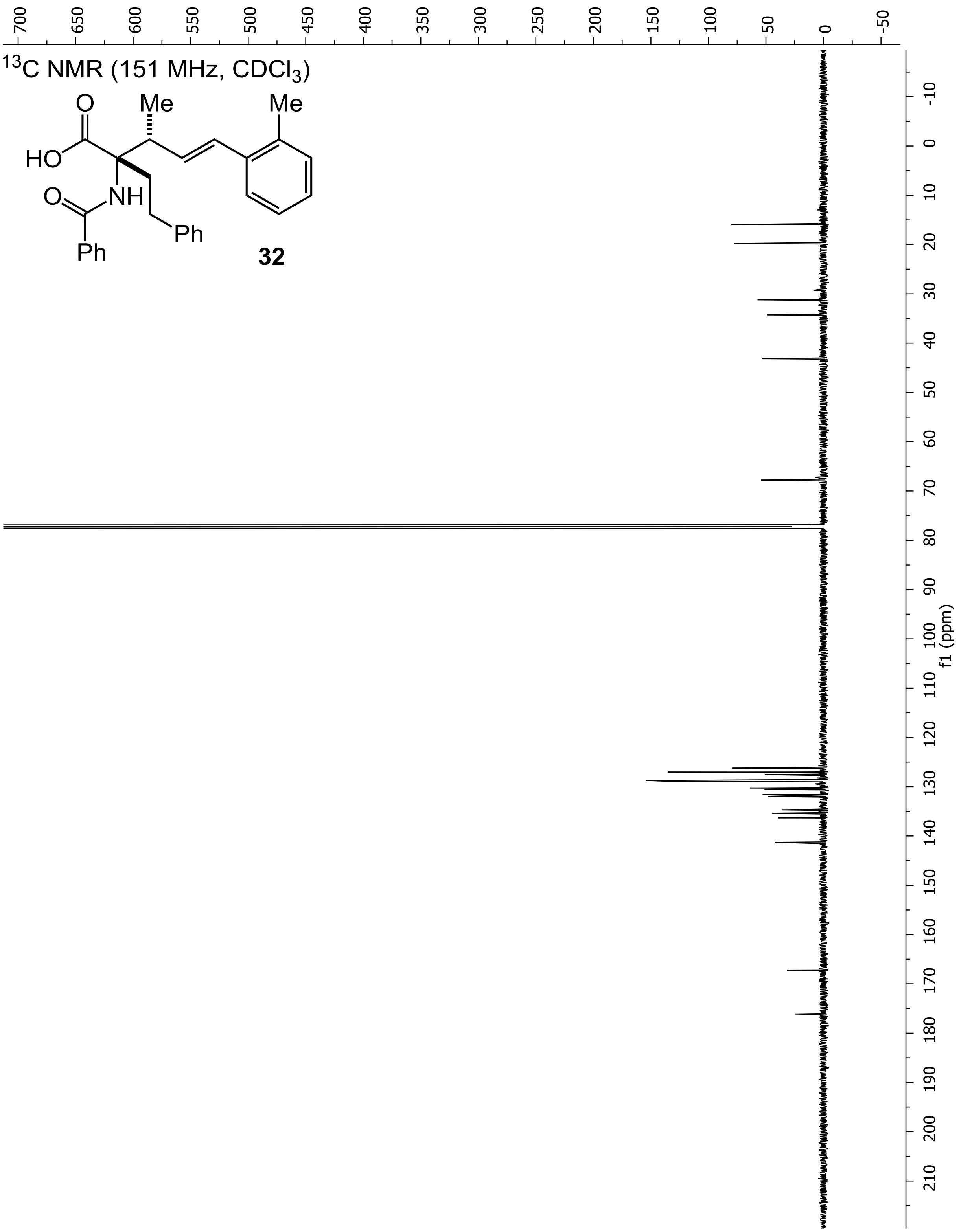

^{13}C NMR (151 MHz, CDCl$_3$)
O
Me
Me
HO
O
NH
Ph
Ph
32
f1 (ppm)

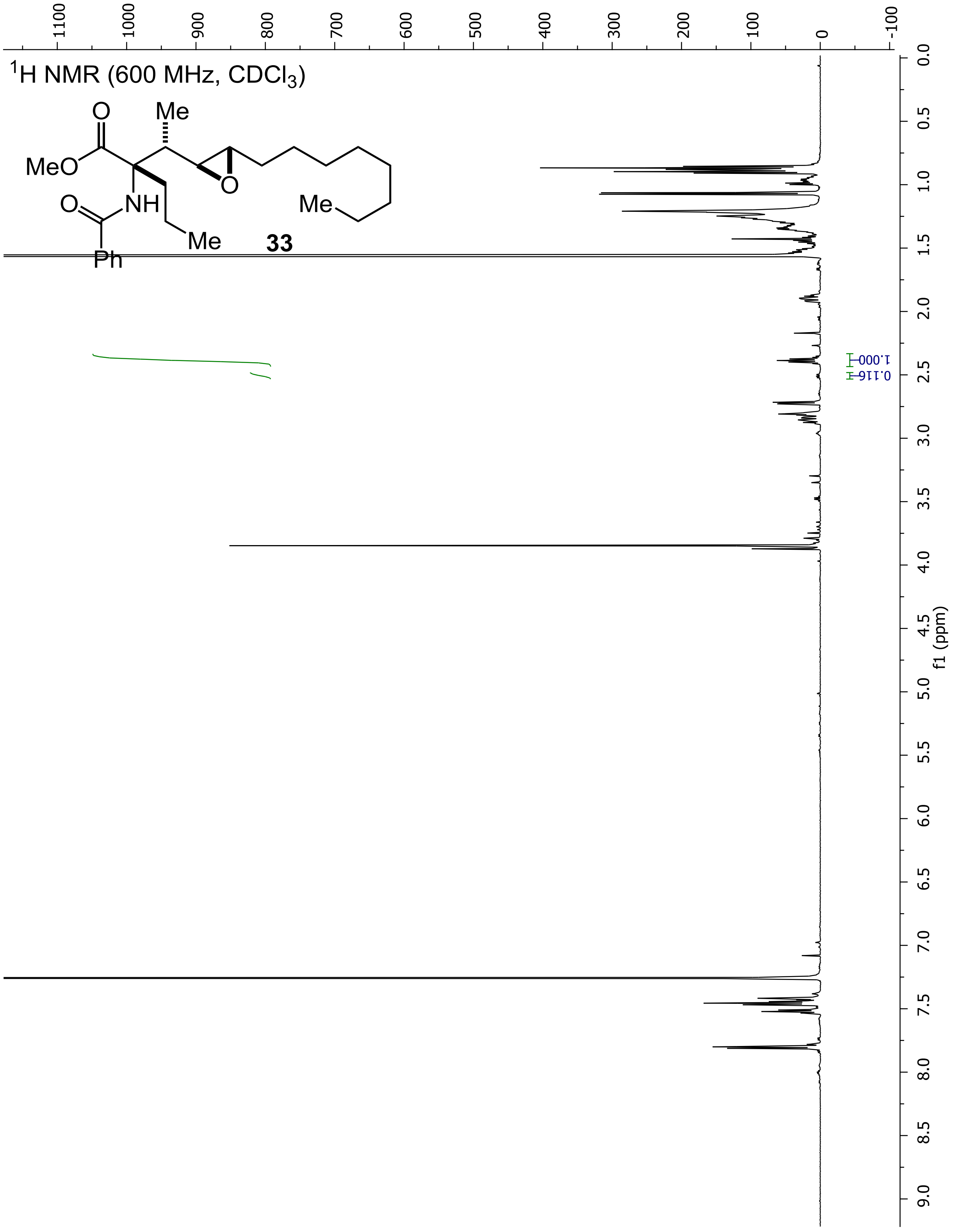
1H NMR (600 MHz, CDCl3)
MeO
O
Me
NH
O
Me
Me
Ph
33
f1 (ppm)
1.000
0.116

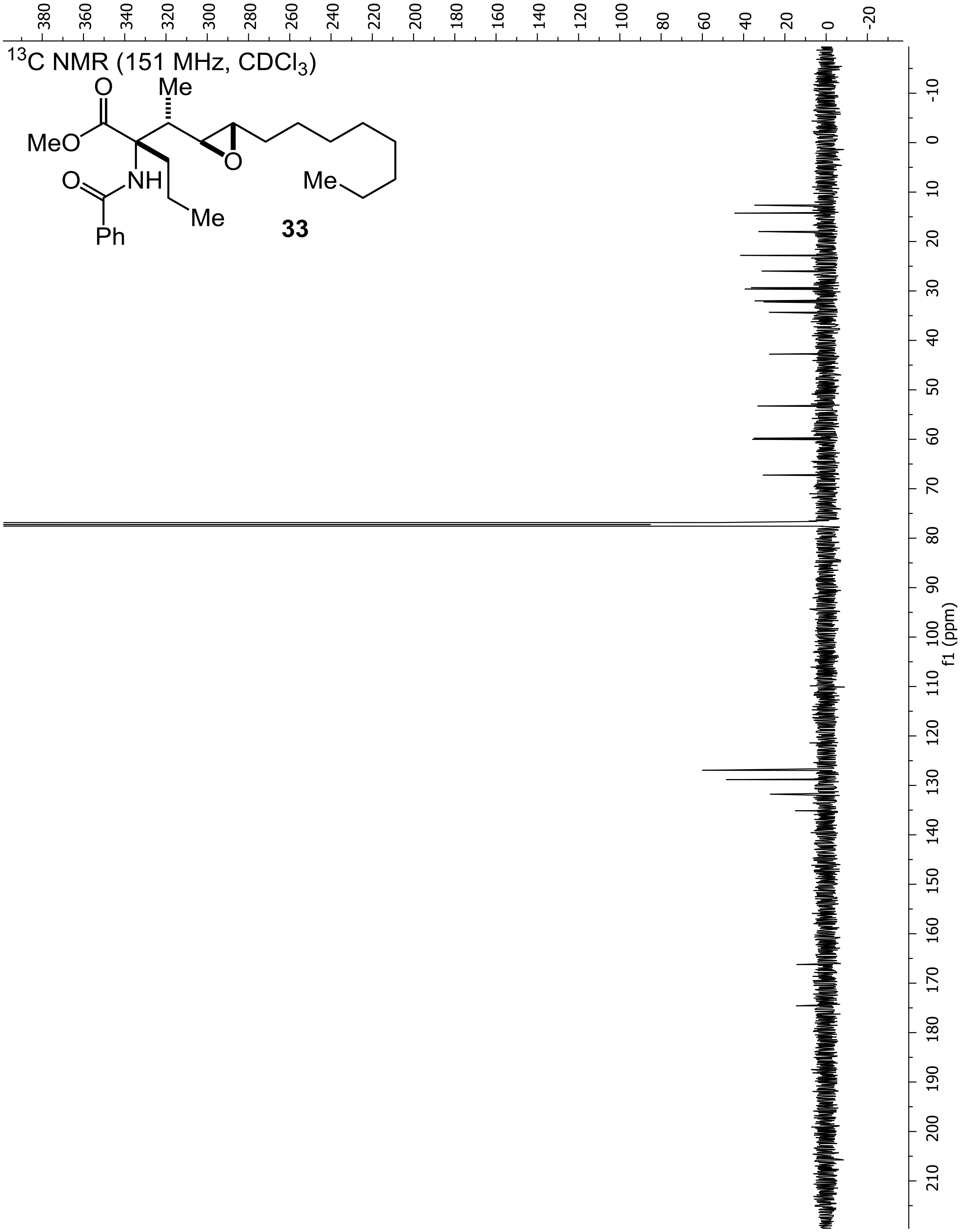

^{13}C NMR (151 MHz, CDCl$_3$)
33
f1 (ppm)
